# Perhalogenated Anilines as Bifunctional Donors of Hydrogen and Halogen Bonds in Cocrystals with Ditopic Nitrogen-Containing Acceptors

**DOI:** 10.1021/acs.cgd.4c00315

**Published:** 2024-06-06

**Authors:** Nea Baus Topić, Sibananda G. Dash, Edi Topić, Mihails Arhangelskis, Dominik Cinčić

**Affiliations:** † Department of Chemistry, Faculty of Science, University of Zagreb, Horvatovac 102a, 10000 Zagreb, Croatia; ‡ Faculty of Chemistry, University of Warsaw, 1 Pasteura Street, Warsaw 02-093, Poland

## Abstract

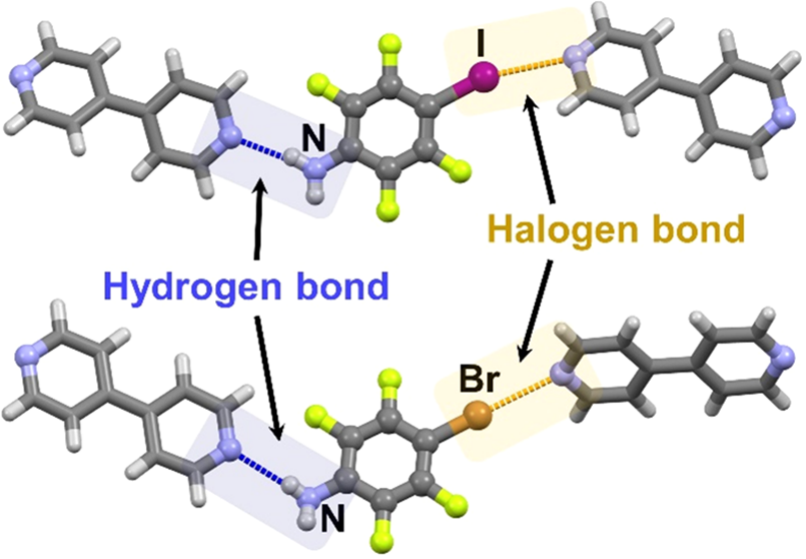

In this study, we examine the experimental and theoretical capabilities of two perhalogenated anilines, 2,3,5,6-tetrafluoro-4-bromoaniline (**btfa**) and 2,3,5,6-tetrafluoro-4-iodoaniline (**itfa**) as hydrogen and halogen bond donors. A series of 11 cocrystals derived from the two anilines and selected ditopic nitrogen-containing acceptors (4,4′-bipyridine, 1,2-bis(4-pyridyl)ethane, and 1,4-diazabicyclo[2.2.2]octane) in 1:1 and 2:1 stoichiometries were prepared by liquid-assisted grinding and crystallization from solution. Crystallographic analysis revealed bifunctional donor properties in both anilines. The dominant supramolecular interaction in four cocrystals of **btfa** is the N–H···N_acceptor_ hydrogen bond between **btfa** and acceptor molecules, while in the one remaining cocrystal, donor and acceptor molecules are connected via the N–H···N_acceptor_ hydrogen bond and the Br···N_acceptor_ halogen bond. In two cocrystals of **itfa**, the dominant supramolecular interaction is the I···N_acceptor_ halogen bond between **itfa** and acceptor molecules, while in the remaining four cocrystals, donor and acceptor molecules are additionally connected by the N–H···N_acceptor_ hydrogen bond. Periodic density-functional theory (DFT) calculations have been conducted to assess the formation energies of these cocrystals and the strengths of the established halogen and hydrogen bonds. Molecular DFT calculations on **btfa** and **itfa** indicate that the differences in electrostatic potential between the competing sites on the molecules are 261.6 and 157.0 kJ mol^–1^ e^–1^, respectively. The findings suggest that **itfa**, with a smaller electrostatic potential difference between donor sites, is more predisposed to act as a bifunctional donor.

## Abstract 2

The experimental and theoretical capability of two perhalogenated anilines, 2,3,5,6-tetrafluoro-4-bromoaniline and 2,3,5,6-tetrafluoro-4-iodoaniline, as hydrogen and halogen bond donors have been explored by cocrystallization with 4,4′-bipyridine, 1,2-bis(4-pyridyl)ethane, and 1,4-diazabicyclo[2.2.2]octane.

## Introduction

Intermolecular interactions represent the main tool in synthetic crystal engineering, given that they are the basis of molecular recognition processes as well as the organization and assembly of molecular building units into more complex supramolecular structures.^1−3^ In general, intermolecular interactions include a wide range of attractive and repulsive forces acting between molecules and can be categorized into strong and weak noncovalent interactions.^4^ While weak interactions such as dispersion forces predominantly contribute to the overall crystal energy, strong noncovalent interactions like hydrogen (HB) and halogen bonds (XB) determine how molecules will connect in the crystal structure.^5−7^ Studying such interactions enables a reliable prediction of how specific functional groups will connect. By applying this knowledge, it is possible to design and prepare crystals with desired structures and properties.^8^ Hydrogen and halogen bonds, being quite similar in their predictability and directionality, are the most extensively studied intermolecular interactions.^9,10^^11,12^ Applications of this knowledge are found in crystal engineering of single-component as well as multicomponent crystals of organic^13−17^ and metal–organic compounds.^18−22^ Strengths of HB and XB vary from weak (around 10 kJ mol^–1^) to very strong (>40 kJ mol^–1^).^23,24^ However, there are significant differences between halogen and hydrogen bonds, particularly from a material design standpoint. Due to the localization of the σ-hole, the halogen bonds are more directional than hydrogen bonds and can be easily tuned by exchanging the halogen atom, with halogen bond strengths increasing with the increasing atomic size of halogens.^25−28^ Moreover, halogen bond strengths strongly depend on the electron-withdrawing ability of the moieties bound to halogen atoms^29,30^ and the basicity of halogen bond acceptors.^25,31−33^

Most research so far has been focused on multicomponent systems where either hydrogen bonds or halogen bonds are dominant.^8−10^ In contrast, competition or cooperativity between these two interactions is seldom explored.^34−36^ Specifically, there is a limited number of studies investigating cocrystals of HB/XB bifunctional donors, compounds that have both a hydrogen bond donor group and a halogen bond donor group within the same molecular backbone.^37−44^ Regarding the first case, there is a solid amount of data in the Cambridge Structural Database (CSD) for multicomponent systems containing bromo- and iodoperfluorinated benzenes with various acceptors containing nitrogen, oxygen, sulfur, etc. (1617 data sets).^45^ Constraining the set to the second case, i.e., perhalogenated phenols, benzoic acids, and *N*-(benzylidene)hydroxylamines as simple HB/XB bifunctional donors with nitrogen-containing acceptors, only 50 data sets were found, which represent the majority of systems in which HB/XB bifunctional donors have been investigated. In one of the first pivotal works on studying potential bifunctional donors to establish a relative ranking of competing hydrogen and halogen bonds, Aakeröy and co-workers prepared a family of cocrystals of *N*-perhalogenated *N*-(benzylidene)hydroxylamines with a ditopic asymmetric acceptor (containing pyridyl and benzimidazole nitrogen atoms).^37^ Furthermore, they later expanded their research to systems with perhalogenated phenols and benzoic acids.^38−41^ In their work, they used both monotopic and ditopic nitrogen-containing acceptors and established that donor molecules containing bromine are considerably weaker halogen bond donors than donors containing iodine and consequently do not form halogen bonds that are competitive to hydrogen bonds. Furthermore, they established that the orientation of the binding site in the acceptor can influence the supramolecular assembly. In cocrystals of 3,3′-azobipyridine, hydrogen bonding is the primary driving force, while in cocrystals of 4,4′-azobipyridine, both hydrogen and halogen bonds equally contribute to the intermolecular connectivity.^39^ Also, they have established that there is no distinction between three types of hydrogen bond donors (COOH, OH, and CN(R)OH) in terms of binding to acceptors in the presence of halogens. Additionally, computational methods have shown that predicting of the supramolecular outcome of cocrystallization can be achieved by determining the relative difference in molecular electrostatic potentials between two nitrogen atoms of acceptor molecules, as well as between hydrogen and halogen bond donor groups. Halogen- and hydrogen-bonded cocrystals involving 4-iodotetrafluorobenzoic acid, 4-iodotetrafluorophenol, and 4-bromotetrafluorophenol were also the subject of Bruce and co-workers.^43^ They demonstrated that by choosing building units carefully, halogen bonding between unlike components can be favored over hydrogen bonding and that such results are consistent with both the iodine basicity scale and HSAB concept. Lu and co-workers theoretically investigated cocrystals of already mentioned HB/XB bifunctional donors and two isomeric azobipyridine acceptors.^46^ They established that the introduction of a simple hydrogen bond donor moiety into the backbone of a halogen bond donor molecule should lead to cocrystals in which hydrogen bonds behave as the primary driving force, accompanied by halogen bonds and secondary weak hydrogen bonds.

**Figure 1:**
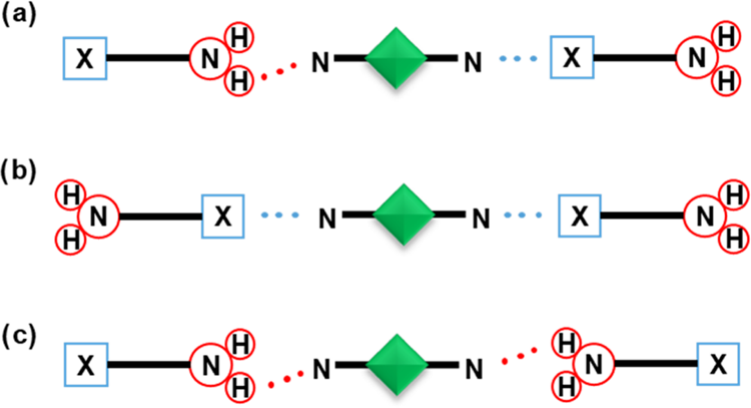
Possible outcomes of cocrystallization of **btfa**/**itfa** with symmetric ditopic acceptors (X = I or Br): (a) both halogen and hydrogen bonds are formed, (b) only halogen bonds are formed, and (c) only hydrogen bonds are formed.

**Scheme 1:**
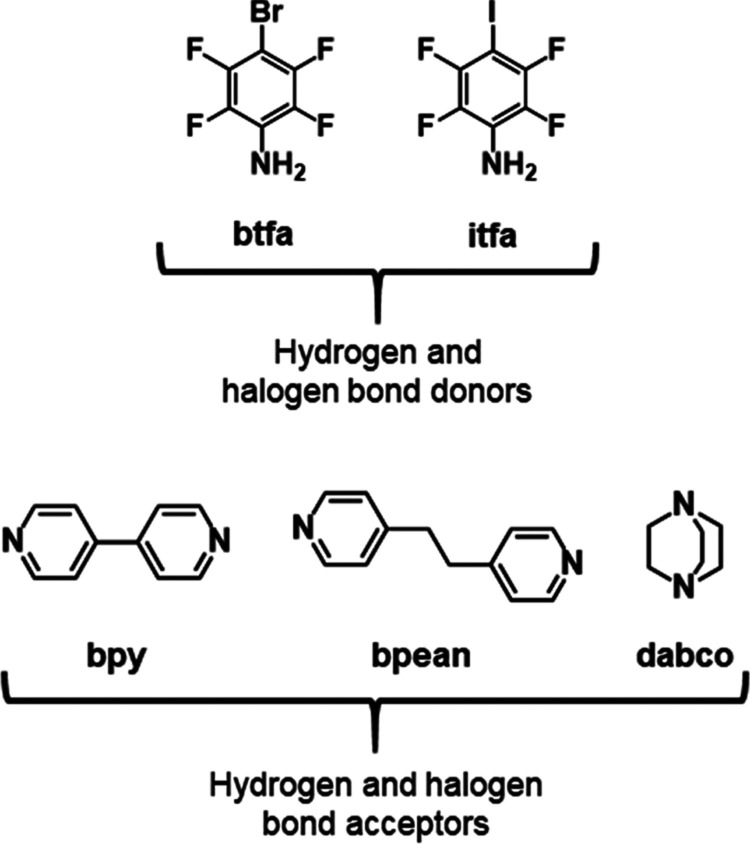
Molecular Schemes of Donors and Acceptors Used in This Study

As a step towards further exploration of the competition/cooperativity between halogen and hydrogen bonding, in this research, we have selected bifunctional donors that have both a halogen atom (Br or I) and an amine group, i.e., 2,3,5,6-tetrafluoro-4-bromoaniline (**btfa**) and 2,3,5,6-tetrafluoro-4-iodoaniline (**itfa**) (Scheme 1). Since HB/XB bifunctional donors containing an amino group as a hydrogen bond donor have not been studied to date, perfluorinated anilines were chosen considering that, in addition to the halogen bond donor group, they contain a hydrogen bond donor group that has two hydrogen atoms available to participate in the formation of hydrogen bonds. For cocrystal formation, as both halogen and hydrogen bond acceptors, we selected three nitrogen-containing ditopic symmetric molecules: 4,4-bipyridine (**bpy**), 1,2-bis(4-pyridyl)ethane (**bpean**), and 1,4-diazabicyclo[2.2.2]octane (**dabco**) (Scheme 1). In order to explore the stoichiometric ratio in the cocrystallization of donor and acceptor pairs, we first performed mechanochemical syntheses by liquid-assisted grinding (LAG)^47,48^ of the reactants in 1:1 and 2:1 donor: acceptor stoichiometric ratios. As previously reported by Aaakeröy and co-workers,^41^ the possible supramolecular outcomes of cocrystallization of donor and acceptors in 1:1 and 2:1 ratios are shown in Figure 1. To facilitate the characterization of the new cocrystals by single-crystal X-ray diffraction, mechanochemical experiments were accompanied by crystallization from the solution. In our previous work, we have demonstrated that periodic density-functional theory (DFT) calculations provide a quantitative evaluation of halogen-bonded cocrystals.^32,49−51^ The obtained cocrystals in this work enabled a unique opportunity for detailed DFT computational studies of HB/XB bifunctional donor molecules, including determination of the formation energies of cocrystals and comparison of energies of relevant interactions.

## Experimental and Computational Methods

### Synthesis

All reagents and solvents, except **itfa**, were purchased from commercial sources. **itfa** was synthesized according to the procedure described by Politanskaya et al.^52^ but was recrystallized from a mixture of water and ethanol.

### Mechanochemical Cocrystal Screening

Initial cocrystal screening was performed by liquid-assisted grinding (LAG) of mixtures of **itfa** or **btfa** with **bpy**, **bpean** and **dabco** in 1:1 and 2:1 stoichiometric ratios. The reaction mixture (80 mg) was placed in a 5 mL stainless steel jar along with 20 μL of acetone and two stainless steel balls 5 mm in diameter, then milled for 30 min in a Retsch MM200 Shaker Mill operating at 25 Hz milling frequency. For grinding **btfa** with **bpean** and **itfa** with **bpy** in 2:1 stoichiometric ratio, 20 μL of acetonitrile, ethanol, and nitromethane were used besides acetone. The resulting powders were characterized by powder X-ray diffraction (PXRD), thermogravimetric analysis (TGA), and differential scanning calorimetry (DSC). Details on mechanochemical experiments are given in SI, Table S1.

### Single-Crystal Preparation

Cocrystals suitable for single-crystal X-ray diffraction experiments were prepared by crystallization from solution. Around 30 mg of a mixture of **itfa** or **btfa** with **bpy**, **bpean**, and **dabco** in either 1:1 or 2:1 stoichiometric ratio was dissolved in 2.0 mL of a corresponding hot solvent, if necessary with the addition of a small amount of product obtained by LAG (seeding). The crystals were obtained by slow evaporation of the solvent at room temperature for a few days. Cocrystals (**btfa**)(**bpy**), (**btfa**)(**bpean**), (**btfa**)_2_(**dabco**), (**itfa**)(**bpy**), (**itfa**)(**bpean**), and (**itfa**)(**dabco**) were prepared from acetone; (**btfa**)_2_(**bpy**) and (**itfa**)_2_(**bpean**) were prepared from acetone (seeding); (**btfa**)(**dabco**) was crystallized from nitromethane, and (**itfa**)_2_(**dabco**) was crystallized from ethanol (seeding). Details on crystallization experiments are given in the SI, Table S2.

### Powder X-ray Diffraction (PXRD)

PXRD experiments were performed on a Malvern Panalytical Aeris Research Edition X-ray diffractometer with Cu Kα_1_ (1.54056 Å) radiation at 15 mA and 40 kV. The scattered intensities were measured with a PixCEL1D HPC detector. The angular range was from 5 to 40° (2θ) with steps of 0.00543° (2θ), and the measuring time was 10.2 s per step. Data analysis was performed using the program package HighScore 4.8.^53^ PXRD patterns are given in SI, Figures S16–S32.

For the determination of the crystal structure model of (**itfa**)_2_(**bpy**), the diffraction data were collected on a Panalytical Empyrean diffractometer equipped with a Mo Kα source with an elliptical W/Si focusing mirror and the GaliPIX3D HPC detector in capillary transmission mode using 1.0 mm thin-walled borosilicate glass capillaries. The data were collected at 298 K. Data collection parameters were: start 2θ angle 2.0°, end 2θ angle 55.0°, step size 0.000719°, and total collection time 5 h. Indexing and refinement was done in TOPAS Academic software v.5.^54^ Space group symmetry was determined based on expected stoichiometry and permitted Wyckoff position multiplicity (4 for **itfa** and 2 for **bpy**). Background scattering intensities were modeled using a modification of a robust Bayesian analysis.^55^ Instrument-related and sample-related peak convolution parameters were refined using the fundamental parameters approach. Molecular geometries (i.e., bond length, angles, and torsion angles) of **itfa** and the pyridyl fragment of **bpy** molecule were fully constrained to those found in crystal structure models of **itfa** and **bpy**.^56,57^ Orientation and position of molecular fragments, as well as the values of unconstrained parameters, were found using a simulated annealing procedure as implemented in TOPAS. Final refinement was conducted by simultaneously refining all instrument, sample, and structure parameters. Details of data collection and crystal structure refinement are listed in Table S5 and Figures S14 and S15 of SI. Molecular structure showing the atom-labeling scheme is given in SI, Figure S13.

### Single-Crystal X-ray Diffraction (SCXRD)

The crystal and molecular structures of the prepared anilines and cocrystals were determined by single-crystal X-ray diffraction. Diffraction measurements were made on a Rigaku Synergy XtaLAB X-ray diffractometer equipped with a Dualflex source and HyPix HPC detector using Mo Kα radiation, λ = 0.71073 Å. The data sets were collected at 170 K using ω scan mode over the 2θ range up to 64° (Synergy XtaLAB). Programs CrysAlis CCD, CrysAlis RED, and CrysAlisPro were employed for data collection, cell refinement, and data reduction, respectively.^58^ The structures were solved by direct methods and refined using the SHELXS, SHELXT, and SHELXL programs, respectively.^59,60^ Structural refinement was performed on *F*^2^ by using all data. Non-hydrogen atoms were refined anisotropically, and hydrogen atoms were either placed in calculated positions and treated as riding on their parent atoms or were located in the Fourier difference maps (amino group) with temperature factors fixed at 1.2 times *U*_eq_ of the parent atom. In the (**btfa**)(**dabco**) cocrystal, two symmetrically inequivalent **dabco** molecules are present and one **dabco** is additionally disordered over two positions. All calculations were performed using the Olex2 crystallographic suite of programs.^61^ The molecular structures of compounds and their molecular packing projections were prepared by Mercury 2022.3.0.^62^ Details of data collection and crystal structure refinement are listed in Tables S3 and S4 of SI. Molecular structures showing the atom-labeling schemes are given in SI, Figures S1–S12. Further details are available from the Cambridge Crystallographic Center (CCDC 2336067–2336079) containing crystallographic data for this paper.

### Intermolecular Contact Analysis

Intermolecular contacts were analyzed using Mercury 2022.3.0.^62^ Contacts that are shorter than the sum of the van der Waals radii of the involved atoms reduced by 0.05 Å were analyzed. For the hydrogen bond analysis, the default definition from Mercury was used.

### Thermogravimetric Analysis (TGA)

TGA measurements were performed on a Mettler-Toledo TGA/DSC 3+ module. The samples were placed in open 70 μL alumina pans and heated from 30 to 300 °C at a rate of 10 °C min^–1^ under nitrogen flow of 50 mL min^–1^. Data collection and analysis were performed using the program package STARe Software v16.30.^63^ TGA spectra are given in SI, Figures S33–S48.

### Differential Scanning Calorimetry (DSC)

DSC measurements were performed on a TA Instruments Discovery DSC 25. The samples were placed in hermetically sealed 40 μL TA zero aluminum pans and heated from 25 °C to temperatures a few °C above the melting point at a rate of 10 °C min^–1^ under nitrogen flow of 50 mL min^–1^. Data collection and analysis were performed using the program package TRIOS Software v5.1.1.^64^ DSC curves are given in SI, Figures S49–S64.

### Computational Details

The crystallographic information files (CIFs) for **btfa**, **itfa**, and the cocrystals were obtained either from the diffraction measurements performed in the current work or, in the case of other coformers, taken from the Cambridge Structural Database (CSD, version 2022).^45^ Covalent hydrogen bond lengths were normalized to their neutron diffraction values using Mercury^62^ and then the CIFs were converted into the input format of CASTEP using cif2cell utility^65^ prior to geometry optimization. The (**btfa**)(**dabco**) cocrystal was specially treated. The structure of this cocrystal was transferred to the primitive space group *P*1 from *C*2/*c* and then one set of equivalent atoms were removed for each **dabco** molecule, with the aim of resolving the positional disorder. Finally, missing hydrogen atoms were added to the **dabco** molecules prior to the CASTEP input file generation. The unit cell parameters and atomic coordinates were optimized with a constraint on crystal symmetry. The calculations were performed at 700 eV plane-wave basis cutoff with Perdew–Burke–Ernzerhof (PBE) functional^66^ for both Grimme dispersion correction (D3)^67^ and many-body dispersion correction (MBD*)^68−70^ using CASTEP (version 22.11)^71^ code. The convergence parameters for the calculation were set as follows: energy = 2 × 10^–5^ eV atom^–1^, force = 5 × 10^–2^ eV Å^–1^, displacement = 10^–3^ Å, and stress = 10^–2^ GPA. The formation energies of the cocrystals were calculated as the energy of cocrystals reduced by the sum of energy of coformers. Energies of the cocrystals and their constituent components are given in the SI, Table S6. Further, intermolecular interaction energies of the noncovalent bonded molecular dimers were calculated using the PBE/MBD* method starting from the energy of the minimized geometry. Single-point energies of the noncovalently bonded dimers or their respective monomers were calculated by placing the molecules in a 30 × 30 × 30 Å^3^ box. The cell parameters were kept fixed throughout the calculation, and the k-point grid was only with a Γ point in this case. All other parameters were kept the same as those used for the periodic calculations (see SI, Figure S65–S85).

### Molecular Electrostatic Potential Calculations

Molecular geometries of the cocrystal formers were taken from the hydrogen bond normalized CIFs, and the energy was minimized using the PBE functional with Def2TZVP basis set.^72^ The calculations were performed using Gaussian16^73^ DFT code. Check point files obtained from the calculation were converted into formatted checkpoint files and were used to generate cube files for both electron density and electrostatic potential with the help of the *cubegen* utility. The cube files were used to plot the electrostatic potential plots with the help of *MolecoolQT* software^74^ package. The electrostatic potential was plotted on the electron density isosurface of 0.01 au. A seven-colored rainbow scheme was adopted for the plotting, and the scale was adjusted between min: – 0.02 au and max: 0.2 au.

## Results and Discussion

**Figure 2:**
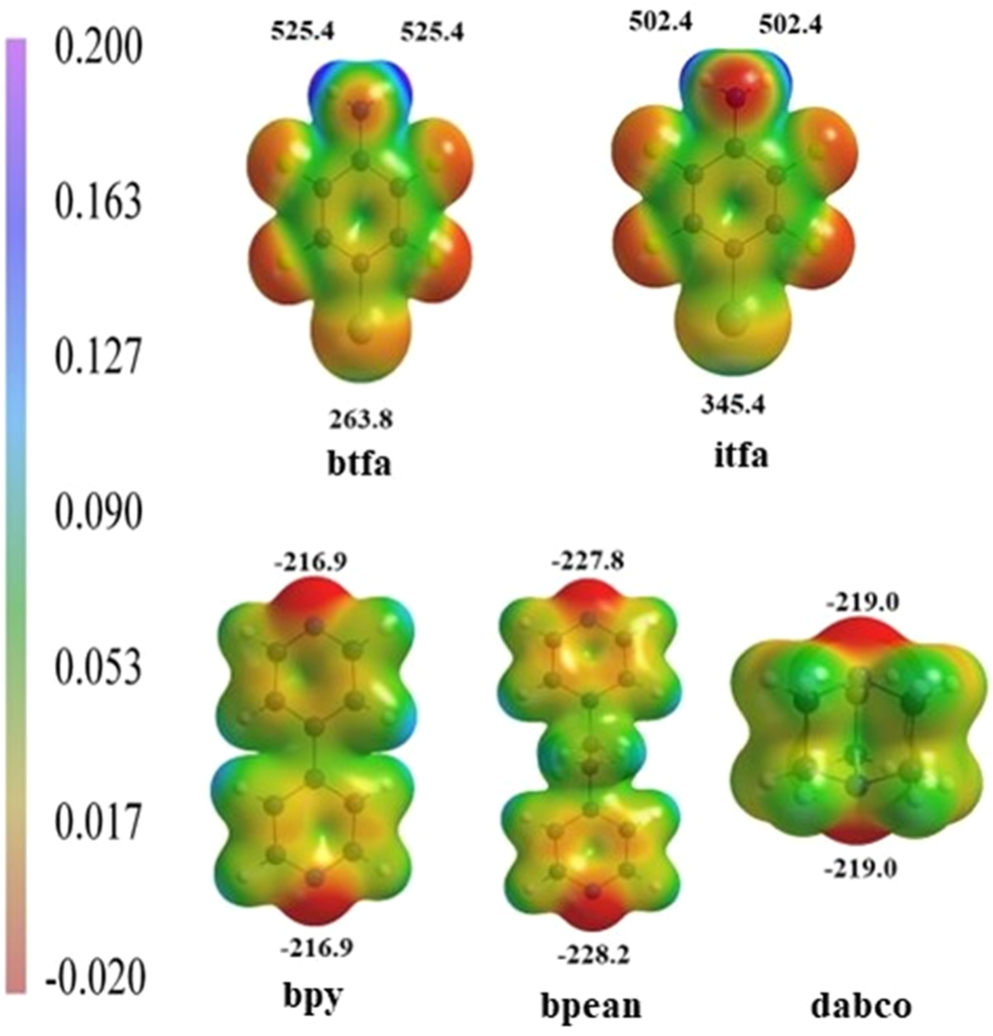
Molecular electrostatic potentials of the optimized **btfa**, **itfa**, **bpy**, **bpean**, and **dabco** molecules, plotted on the electron density isosurface of 0.01 au. All values are in kJ mol^–1^ e^–1^.

In order to investigate the potential of amino group hydrogen atoms as hydrogen bond donors, as well as bromine or iodine atoms as halogen bond donors, molecular electrostatic potentials (MEPs) of optimized geometries of **btfa** and **itfa** molecules have been calculated *in vacuo* (Figure 2). These results suggest that the most positive potential binding sites in donor molecules are amino group hydrogen atoms, while bromine and iodine are significantly less positive. When comparing **btfa** and **itfa**, it can be seen that the iodine is more positive than the bromine atom, which is in accordance with the data available in the literature on iodine being the better halogen bond donor due to a greater σ-hole. The difference between the two potential donor sites in **btfa** (261.6 kJ mol^–1^ e^–1^) is greater than the one in **itfa** (157.0 kJ mol^–1^ e^–1^). As shown in previous studies of ditopic hydrogen/halogen bond acceptors and donors, electrostatic factors are important in defining the structural environment of molecules that can engage in competing intermolecular interactions as long as the difference between potentially competing sites is large enough. Applying the same reasoning to the potential bifunctional donors, **itfa** and **btfa**, the electrostatic potential difference in **btfa** is much greater than in **itfa**. Thus, it can be expected that in most cases, only hydrogen bonds will be formed. On the other hand, for **itfa**, it is more likely that there will not be a pronounced intermolecular preference for either hydrogen or halogen bonding, leading to less selectivity. Following that, it can be expected that **itfa** would be a better bifunctional donor molecule. MEPs of optimized acceptor molecules **bpy**, **bpean**, and **dabco** were also calculated and show that the most negative binding sites in each molecule are nitrogen atoms, with values being −228.2 kJ mol^–1^ e^–1^ in **bpean**, –219.0 kJ mol^–1^ e^–1^ in **dabco**, and −216.9 kJ mol^–1^ e^–1^ in **bpy**. These results suggest that the three selected acceptor molecules have similar potentials to form intermolecular interactions.

To examine whether the two selected donors will act as bifunctional donor molecules in cocrystals with **bpy**, **bpean**, and **dabco**, cocrystallization of **itfa** and **btfa** was explored through mechanochemical synthesis via liquid-assisted grinding as well as by crystallization from solution. For both anilines, mechanochemical screening was conducted using 1:1 and 2:1 stoichiometric ratios of anilines and nitrogen-containing acceptors. Since both donors and acceptors are potential ditopic entities, these stoichiometric ratios were chosen to explore the formation of both 1:1 and 2:1 cocrystals and to determine which supramolecular arrangements would prevail (Figure 1) depending on the stoichiometry of prepared cocrystals. Mechanochemical cocrystal screening experiments began with liquid-assisted grinding of the solid reactants. Grinding experiments with a small amount of acetone led to the formation of 10 novel crystalline phases, while the variation of liquid additive, i.e., nitromethane or ethanol in case of **itfa** and **bpy** in a 2:1 ratio, enabled the formation of an additional crystalline phase, culminating in a total of 11 novel cocrystals out of 12 possible combinations. When **btfa** was ground with **bpean** in the 2:1 stoichiometric ratio, the outcome was identical to that of the 1:1 synthesis, albeit with an excess amount of **btfa**. Crystallization from solution was subsequently undertaken to prepare crystals suitable for the determination of molecular and crystal structures by single-crystal X-ray diffraction. Crystallizations were performed by simple evaporation methods, usually from acetone and, when needed, with the addition of seeds from mechanochemically prepared products. This allowed us to determine the structure of 10 cocrystals: (**btfa**)(**bpy**), (**btfa**)_2_(**bpy**), (**btfa**)(**bpean**), (**btfa**)(**dabco**), (**btfa**)_2_(**dabco**), (**itfa**)(**bpy**), (**itfa**)(**bpean**), (**itfa**)_2_(**bpean**), (**itfa**)(**dabco**), and (**itfa**)_2_(**dabco**). Solving the structure of the (**btfa**)(**dabco**) cocrystal proved challenging due to the presence of two symmetrically inequivalent **dabco** molecules in the asymmetric unit, modeled with a fixed occupancy of 50%, and one of which was disordered over two positions. Since the structure is highly disordered, placing hydrogen atoms on **dabco** molecules was not possible in the structural model. Additionally, the structure of (**itfa**)_2_(**bpy**) was refined from PXRD data, as our efforts to prepare a single crystal were unsuccessful. The calculated PXRD patterns of these 11 cocrystals were in good agreement with the results from mechanochemical syntheses (up to small differences expected for data collection at different temperatures), thus confirming that all products were obtained as pure single phases (see SI, Figures S21–S32).

**Figure 3:**
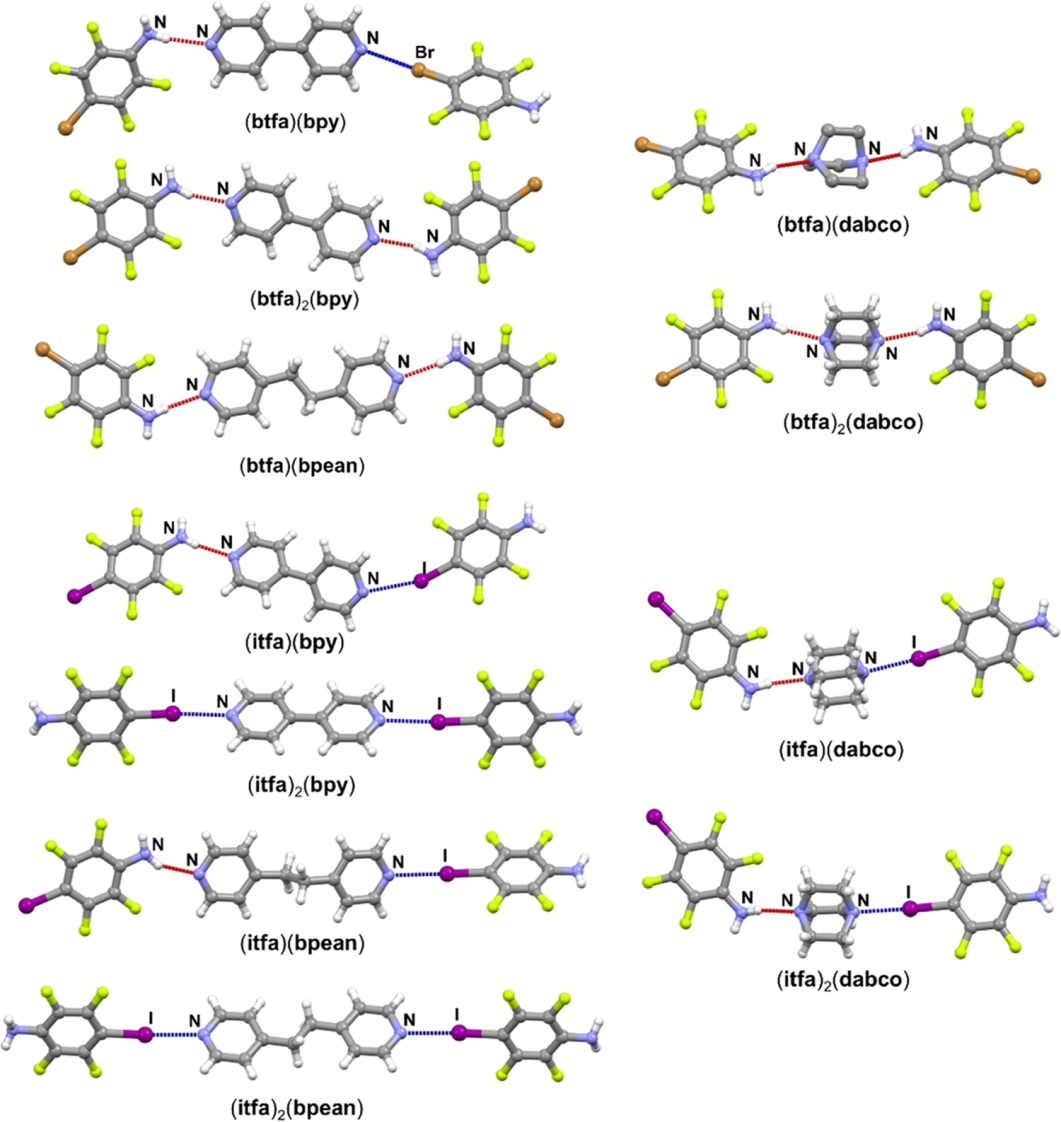
Halogen- and/or hydrogen-bonded assemblies of herein prepared cocrystals. Halogen bonds are shown as blue dotted lines, and hydrogen bonds as red dotted lines.

**Figure 4:**
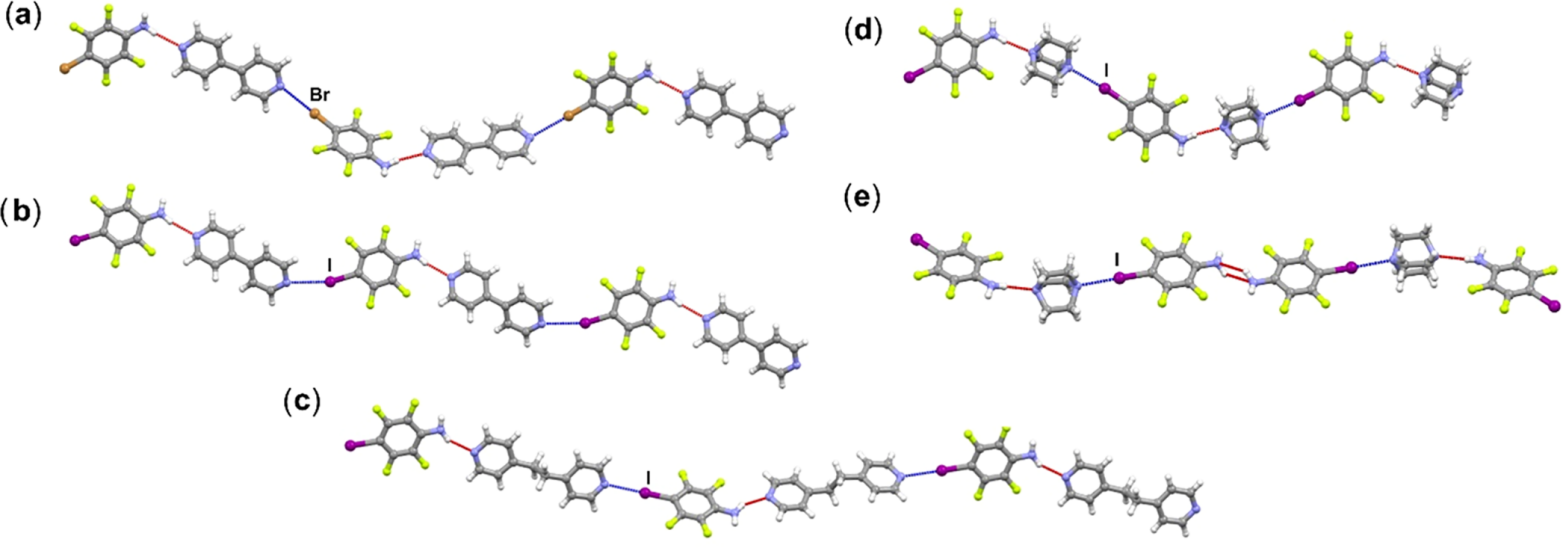
Parts of crystal structures in (a) (**btfa**)(**bpy**), (b) (**itfa**)(**bpy**), (c) (**itfa**)(**bpean**), (d) (**itfa**)(**dabco**), and (e) (**itfa**)_2_(**dabco**).

Structural analysis revealed that donor and acceptor molecules in the cocrystals prepared herein are interconnected through hydrogen and/or halogen bonds. The halogen- and/or hydrogen-bonded assemblies are shown in Figure 3, and the halogen and hydrogen bond parameters are listed in Table 1. In cocrystals (**btfa**)(**bpy**), (**itfa**)(**bpy**), (**itfa**)(**bpean**), (**itfa**)(**dabco**), and (**itfa**)_2_(**dabco**), both halogen and hydrogen bonds are formed between the donor and acceptor molecules, aligning with the XB/HB supramolecular outcome as depicted in Figure 1a. In these 1:1 cocrystals, donor and acceptor molecules form chains through alternating X···N_acceptor_ halogen and N–H···N_acceptor_ hydrogen bonds (Figure 4). In the structures of (**btfa**)(**bpy**), (**itfa**)(**bpy**), and (**itfa**)(**bpean**), halogen and hydrogen-bonded chains are further connected in the third dimension by N–H···F and C–H···F contacts, while in the structure of (**itfa**)(**dabco**), formed chains are connected into a two-dimensional (2D)-network by N–H···F and C–H···F contacts, which are further connected in the third dimension by C–H···I contacts, *d*(C7···I1) = 3.987 Å. In the structure of (**itfa**)_2_(**dabco**), discrete trimers are established between one **dabco** molecule and two **itfa** molecules. Among the two symmetrically inequivalent **itfa** molecules, one participates solely in N–H···N_acceptor_ hydrogen bonding, while the other acts as a bridge between two trimers—it is linked to one **dabco** molecule through I···N_acceptror_ halogen bonding via the iodine atom and to an **itfa** molecule of a neighboring discrete trimer through N–H···N_itfa_ hydrogen bonding between the amino groups (Figure 4e). Formed halogen and hydrogen-bonded hexamers are further connected into a chain by N–H···F contacts, *d*(N2···F1) = 3.424 Å and *d*(N1···F8) = 3.298 Å, which are further connected in the third dimension by C–H···I and C–H···F contacts, *d*(C13···I2) = 4.090 Å. This group of cocrystals gives about 46% of the total 11 cocrystals, making it the most likely supramolecular outcome of this family of cocrystals.

**Table 1:** Halogen (XB) and Hydrogen (HB) Bond Lengths (*d*) and Angles (φ)^a^

cocrystal	XB	*d* (XB) (Å)	R.S.^b^ (%)	φ (XB) (deg)	HB	*d* (HB) (Å)	φ (HB) (deg)
(**btfa**)(**bpy**)	Br1···N3	3.031(6)	10.9	176.4(2)	N1–H1B···N2	3.039(8)	164(6)
(**btfa**)_2_(**bpy**)	N/A				N1–H1A···N3	2.988(4)	163(3)
					N2–H2B···N4	2.995(4)	162(3)
					N1A–H1AB···N3A	3.000(5)	162(4)
					N2A–H2AA···N4A	3.025(6)	165(4)
(**btfa**)(**bpean**)	N/A				N1–H1A···N2	3.036(4)	172(3)
					N1–H1B···N3	3.024(4)	173(3)
(**btfa**)(**dabco**)	N/A				N1–H1A···N3	2.96(1)	159(7)
(**btfa**)_2_(**dabco**)	N/A				N1–H1B···N3	2.925(3)	168(2)
					N2–H2A···N4	2.941(2)	165(2)
(**itfa**)(**bpy**)	I1···N3	3.072(5)	13.0	162.7(1)	N1–H1A···N2	2.982(7)	163(4)
(**itfa**)_2_(**bpy**)	I1···N2	2.8687	18.7	175.66	N/A		
(**itfa**)(**bpean**)	I1···N2	2.872(5)	18.6	175.9(2)	N1–H1B···N3	2.917(8)	153(6)
(**itfa**)_2_(**bpean**)	I1···N2	2.816(4)	20.2	177.0(1)	N/A		
(**itfa**)(**dabco**)	I1···N2	2.799(2)	20.7	171.31(6)	N1–H1B···N3	2.981(3)	172(2)
(**itfa**)_2_(**dabco**)	I1···N3	2.757(2)	21.9	175.75(6)	N2–H2A···N4	3.016(2)	166(2)

a Relative shortenings (R.S.) of the XB distances in the cocrystals prepared cocrystals.

b R.S. = 1 – *d*(XB)/[*r*_vdW_(Br/I) + *r*_vdW_(N)].

**Figure 5:**
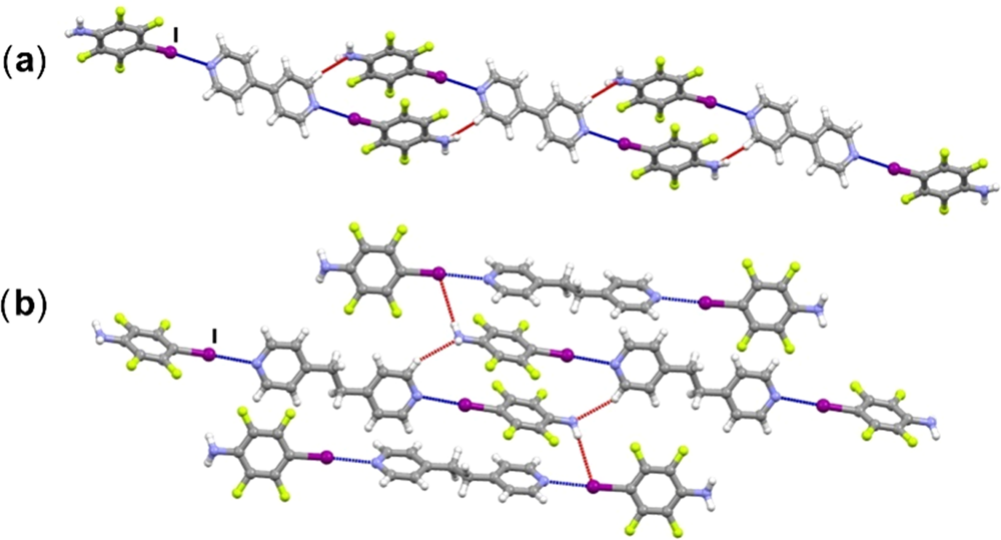
Parts of crystal structures in (a) (**itfa**)_2_(**bpy**) and (b) (**itfa**)_2_(**bpean**).

The XB/XB supramolecular outcome (Figure 1b) occurs in two cocrystals, (**itfa**)_2_(**bpy**) and (**itfa**)_2_(**bpean**), where discrete trimers are formed through I···N_acceptor_ halogen bonding. In the structure of (**itfa**)_2_(**bpy**), halogen-bonded trimers are further connected into a chain by C–H···N_itfa_ contacts, *d*(C7···N1) = 3.676 Å (Figure 5a), which are further connected in the third dimension by N–H···F and C–H···F contacts, *d*(N1···F2) = 3.186 Å. In the structure of (**itfa**)_2_(**bpean**), halogen-bonded trimers are connected into a 2D-network by N–H···I hydrogen bonds and C–H···N_itfa_ contacts, *d*(N1···I1) = 3.821 Å and *d*(C11···N1) = 3.608 Å (Figure 5b), which are further connected in the third dimension by C–H···F contacts.

**Figure 6:**
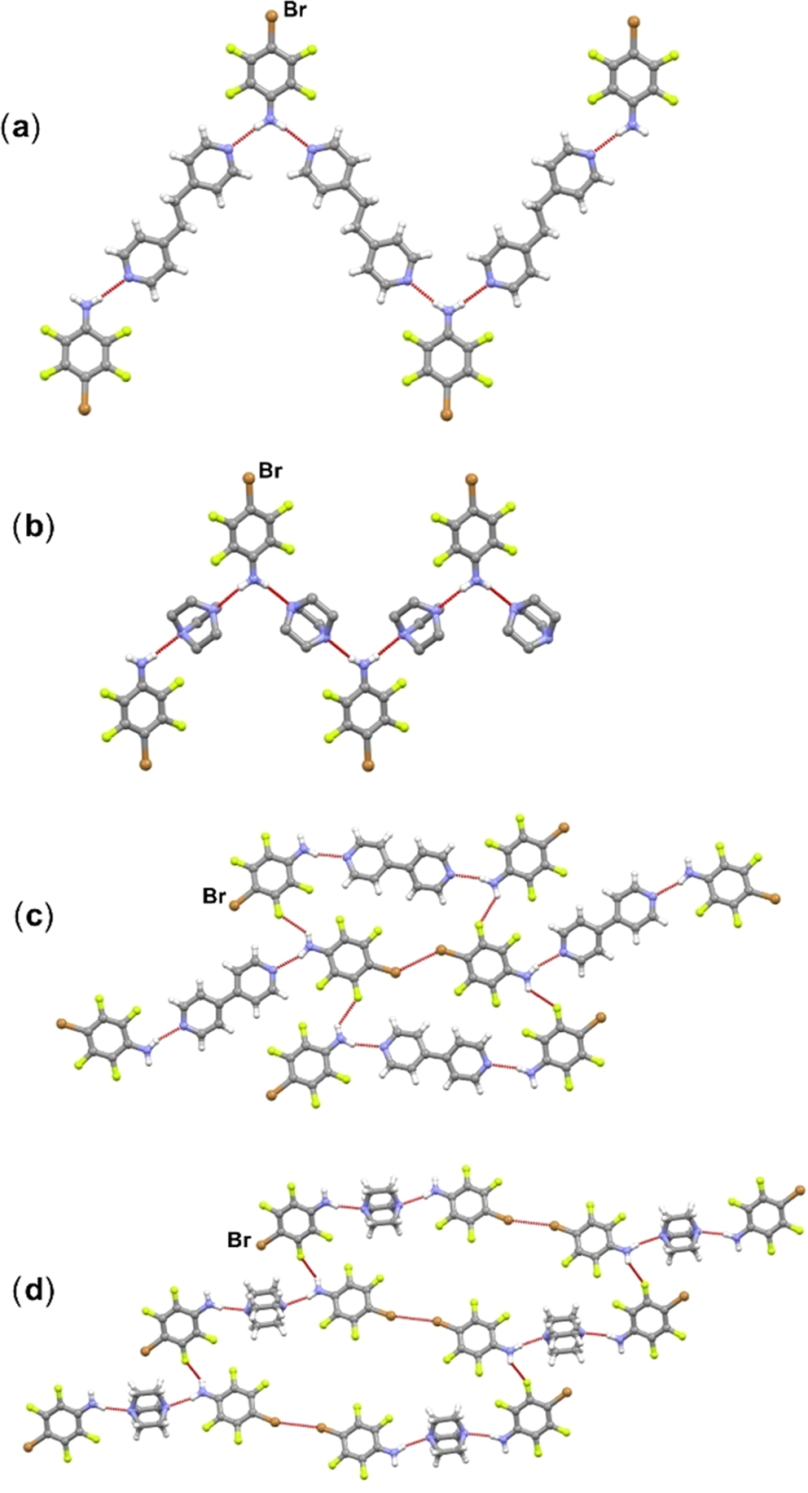
Parts of crystal structures in (a) (**btfa**)(**bpean**), (b) (**btfa**)(**dabco**), (c) (**btfa**)_2_(**bpy**), and (d) (**btfa**)_2_(**dabco**).

Finally, the HB/HB supramolecular outcome (Figure 1c) is featured in (**btfa**)(**bpean**), (**btfa**)(**dabco**), (**btfa**)_2_(**bpy**), and (**btfa**)_2_(**dabco**) cocrystals. In the structures of (**btfa**)(**bpean**) and (**btfa**)(**dabco**), each **btfa** molecule engages in N–H···N_acceptor_ hydrogen bonding via both hydrogen atoms from the amino group, leading to the formation of hydrogen-bonded zigzag chains (Figure 6a,b). In the structure of (**btfa**)(**bpean**) hydrogen-bonded chains are further connected into a 2D-network by C–H···Br and C–H···F contacts, *d*(C9···Br1) = 3.752 Å and *d*(C18···Br1) = 3.755 Å, while in the structure of (**btfa**)(**dabco**), hydrogen-bonded chains are connected into a three-dimensional (3D)-network by C–H···F and Br···F contacts. In the remaining cocrystals (**btfa**)_2_(**bpy**) and (**btfa**)_2_(**dabco**), discrete hydrogen-bonded trimers are formed. In the structure of (**btfa**)_2_(**bpy**), trimers are connected into a 2D-network by N–H···F, C–H···F, and Br···Br (type I) contacts, while in the structure of (**btfa**)_2_(**dabco**), trimers are connected into a chain by N–H···F contacts, *d*(N2···F6) = 3.186 Å and *d*(N1···F2) = 3.115 Å, which are further connected in the second and third dimension by Br···Br (type I) and C–H···F contacts (Figure 6c,d). The HB/HB outcome accounts for 36% of the 11 cocrystals. Even based on this short summary of results, it is evident that **itfa** is more likely to be a bifunctional donor molecule, involved in both halogen bonding via the iodine atom and in hydrogen bonding via the amino group.

Furthermore, the energies of formation as well as the energies of hydrogen and/or halogen bonds were calculated. Periodic DFT calculations using the PBE functional combined with either Grimme D3 or many-body dispersion (MBD*) semiempirical correction schemes were performed for the cocrystal structures as well as for the corresponding individual component structures. The energies calculated with both PBE + D3 and PBE + MBD* methods were in good agreement with each other (SI, Tables S6 and S7). All cocrystals displayed negative formation energies from the starting materials. In order to compare the stability of the pairs of cocrystals containing the same molecular components and differing in stoichiometry (2:1 and 1:1), we also reported the formation energies, normalized per number of molecules contained in the cocrystal formula unit (SI, Table S7). It was found that for the **btfa**/**bpy**, **itfa**/**bpy**, and **itfa**/**bpean** systems, the normalized formation energies for the 2:1 and 1:1 cocrystals were similar, with a difference of 2.5 kJ mol^–1^ or less. Conversely, in the case of the **btfa**/**dabco** system, the 2:1 cocrystal was found to be 7.0 kJ mol^–1^ more stable than its 1:1 counterpart based on PBE + MBD* calculated energies. On the other hand, for the **itfa**/**dabco** system, it was the 1:1 cocrystal that was found to be 7.0 kJ mol^–1^ more stable than the 2:1 form. Based on these results, we conclude that stability of the 2:1 and 1:1 cocrystals does not follow a set trend and is very system-specific.

The calculation of hydrogen and halogen bond energies gave insight into the strength of the interactions (Table 2). In cocrystals with **btfa**, only in (**btfa**)(**bpy**) were both the I···N_acceptor_ halogen and N–H···N_acceptor_ hydrogen bonds formed, with the halogen bond being much weaker than the hydrogen bond. Interestingly, of all hydrogen bonds in **btfa** cocrystals, the N–H···N_acceptor_ hydrogen bond in (**btfa**)(**bpy**), the only cocrystal in which **btfa** is a bifunctional donor, was the weakest. The interplay of intermolecular interactions in cocrystals derived from **itfa** showed greater diversity, as expected, based on the difference in electrostatic potential values of the donor sites. Cocrystals of **itfa** were prepared in both stoichiometric ratios. In (**itfa**)_2_(**bpy**) and (**itfa**)_2_(**bpean**), only I···N_accceptor_ halogen bonds were formed between donor and acceptor molecules, while in (**itfa**)(**bpy**), (**itfa**)(**bpean**), (**itfa**)(**dabco**), and (**itfa**)_2_(**dabco**) both targeted intermolecular interactions were established. In (**itfa**)(**bpy**) and (**itfa**)(**bpean**) cocrystals, the N–H···N_accceptor_ hydrogen bond strength is greater than the I···N_accceptor_ halogen bond strength, while in the (**itfa**)(**dabco**) cocrystal, the strengths of both interactions are almost the same. Surprisingly, in (**itfa**)_2_(**dabco**), the strength of the I···N_accceptor_ halogen bond is greater than that of the N–H···N_accceptor_ hydrogen bond. These results show that in **itfa** cocrystals the preferred interaction is the halogen bond, but **itfa** can also be used as a reliable bifunctional donor molecule through stoichiometry control. In the case of **itfa** cocrystals with 1:1 stoichiometry, supramolecular chains with alternating halogen and hydrogen bonds were reliably formed. Expectedly, in **btfa** cocrystals, the occurrence of halogen bonding was smaller, which is in accordance with calculated electrostatic potentials for donor sites (Figure 2) and overall, with bromine being a known weaker halogen bond donor.

**Table 2:** Halogen (XB) and Hydrogen (HB) Bond Energies of Heteromolecular Noncovalent Bonded Dimers Used in the Calculation of Interaction Energies from Optimized Geometries of Cocrystals

cocrystal	XB	*E* (XB) (kJ mol^–1^)	HB	*E* (HB) (kJ mol^–1^)
(**btfa**)(**bpy**)	Br1···N3	18.70	N1–H1B···N2	37.56
(**btfa**)_2_(**bpy**)	N/A		N1–H1A···N3	39.98
			N2–H2B···N4	39.15
			N1A–H1AB···N3A	39.77
			N2A–H2AA···N4A	39.65
(**btfa**)(**bpean**)	N/A		N1–H1A···N2	39.04
			N1–H1B···N3	40.16
(**btfa**)(**dabco**)	N/A		N–H···N	30.5–42.0
(**btfa**)_2_(**dabco**)	N/A		N1–H1B···N3	44.58
			N2–H2A···N4	44.58
(**itfa**)(**bpy**)	I1···N3	24.73	N1–H1A···N2	40.12
(**itfa**)_2_(**bpy**)	I1···N2	33.39	N/A	
(**itfa**)(**bpean**)	I1···N2	32.23	N1–H1B···N3	37.99
(**itfa**)_2_(**bpean**)	I1···N2	34.64	N/A	
(**itfa**)(**dabco**)	I1···N2	44.13	N1–H1B···N3	44.08
(**itfa**)_2_(**dabco**)	I1···N3	45.91	N2–H2A···N4	42.74

Thermal stability of the perhalogenated amines, symmetric ditopic acceptors, and their cocrystals was investigated by thermogravimetric analysis and differential scanning calorimetry. All prepared cocrystals, regardless of the stoichiometric ratio of donors and acceptors, decompose in one step and have clearly distinguishable signals corresponding to melting (Table 3, SI, Figures S33–S64). The melting points of the cocrystals derived from **btfa** and **bpy**, as well as those of **bpean**, are located between the melting points of the coformers, while the melting points of (**btfa**)(**dabco**) and (**btfa**)_2_(**dabco**) are higher than the melting points of **btfa** and **dabco**. Cocrystals derived from **itfa**, in which both hydrogen and halogen bonds are formed (**itfa**)(**bpy**), (**itfa**)(**bpean**), (**itfa**)(**dabco**), and (**itfa**)_2_(**dabco**), melt at temperatures higher than the melting points of the starting coformers. Cocrystals (**itfa**)_2_(**bpy**) and (**itfa**)_2_(**bpean**), in which halogen bonds predominantly form, have lower melting points than the analogous cocrystals with a stoichiometric ratio of 1:1 and melt at temperatures between the melting points of **itfa** and **bpy**, or **bpean**. The most thermally stable cocrystal is (**itfa**)(**dabco**), which melts at ∼150 °C. Generally, of the 11 prepared cocrystals, those in which hydrogen bonds are formed between donors and acceptors are thermally more stable compared with those in which halogen bonds predominantly form.

**Table 3:** Melting Onset Temperatures (*T*_e_) of Pure Coformers and Respective Cocrystals, as Determined by DSC Analyses

compound	*T*_e_ (°C)	compound	*T*_e_ (°C)
**btfa**	58.0	(**btfa**)(**dabco**)	98.6
**itfa**	70.7	(**btfa**)_2_(**dabco**)	94.3
**bpy**	109.4	(**itfa**)(**bpy**)	119.1
**bpean**	110.7	(**itfa**)_2_(**bpy**)	83.9
**dabco**	77.5	(**itfa**)(**bpean**)	114.3
(**btfa**)(**bpy**)	94.4	(**itfa**)_2_(**bpean**)	102.3
(**btfa**)_2_(**bpy**)	96.6	(**itfa**)(**dabco**)	149.7
(**btfa**)(**bpean**)	95.0	(**itfa**)_2_(**dabco**)	92.5

## Conclusions

The family of 11 **btfa** and **itfa** cocrystals prepared herein, with selected ditopic nitrogen-containing acceptors, demonstrates that both molecules can be bifunctional donors. Out of the six cocrystals obtained with **itfa**, which has a ΔMEP value between the competing donor sites on the molecule of 157.0 kJ mol^–1^ e^–1^, in two cases, the dominant supramolecular interaction is the I···N_accceptor_ halogen bond, while in the remaining four cocrystals, molecules are additionally connected by the N–H···N_accceptor_ hydrogen bonds. In 2:1 cocrystals, the halogen bond is the predominant interaction. On the other hand, in the five cocrystals obtained with **btfa**, which shows a ΔMEP value between the competing sites of 261.6 kJ mol^–1^ e^–1^, the dominant interaction is the hydrogen bond with the exception of the (**btfa**)(**bpy**) cocrystal, where both Br···N_accceptor_ halogen bond and N–H···N_accceptor_ hydrogen bond were found. Consistent with previous studies on HB/XB bifunctional donors, it is once more confirmed that a larger difference in electrostatic potentials at potential donor sites on the same molecule favors the formation of a singular preferred intermolecular interaction. The calculated energies of hydrogen and halogen bonds indicate that when present, hydrogen bonds are generally the stronger interactions in the cocrystals. This research significantly contributes to our understanding of the hierarchy and competition between hydrogen and halogen bonds in cocrystals of perfluorinated donor molecules containing both an amino group and a halogen atom (Br or I).

## Supporting Information Available

Experimental details, including syntheses, single-crystal diffraction data, PXRD patterns, TGA curves, DSC curves, the calculated electronic energies of all the cocrystals and their starting components and noncovalent bonded dimers used for the calculation of interaction energy from optimized geometries of cocrystals (PDF)
