# Supplementary material for: Perhalogenated Anilines as Bifunctional Donors of Hydrogen and Halogen Bonds in Cocrystals with Ditopic Nitrogen-Containing Acceptors
Source: Cryst Growth Des. 2024 Jun 6;24(12):5078–88. doi: 10.1021/acs.cgd.4c00315 (PMC11191752; doi:10.1021/acs.cgd.4c00315)
Supplement: Supplementary file 1 — cg4c00315_si_001.pdf [file cg4c00315_si_001.pdf]

SUPPORTING INFORMATION

Perhalogenated anilines as bifunctional donors of hydrogen and halogen bonds in cocrystals with ditopic nitrogen-containing acceptors

Nea Baus Topić[a], Sibananda G. Dash[b], Edi Topić[a], Mihails Arhangelskis[b]*, Dominik Cinčić[a]*

[a] Department of Chemistry, Faculty of Science, University of Zagreb, Horvatovac 102a, 10000 Zagreb, Croatia

[b] Faculty of Chemistry, University of Warsaw, 1 Pasteura Street, Warsaw 02-093, Poland

E-mail: m.arhangelskis@uw.edu.pl, dominik@chem.pmf.hr

Table of Contents

Item	Page
**Mechanochemical experiments (Table S1.)**	**3**
**Crystallization experiments (Table S2.)**	**5**
**Table S3** Crystal data and refinement details for **btfa** and cocrystals with **btfa**.	**6**
**Table S4** Crystal data and refinement details for **itfa** and cocrystals with **itfa**.	**8**
**Figures S1–S12** ORTEP representations of the formula units of the prepared compounds	**10**
**Table S5** Experimental and crystallographic data for (**itfa**)$_2$(**bpy**) (refined from PXRD data).	**16**
**Figure S13** Molecular structure of (**itfa**)$_2$(**bpy**) showing the atom-labelling scheme for the asymmetric unit.	**16**
**Figures S14-S15** Rietveld refinement data. PXRD data for (**itfa**)$_2$(**bpy**).	**17**
**Figures S16–S32** PXRD patterns of the prepared compounds.	**18**
**Figures S33–S48** TGA curves of the prepared compounds	**27**
**Figures S49–S64** DSC thermograms of the prepared compounds.	**35**
**Table S6** The calculated electronic energies (per formula unit) of all the cocrystals and their starting components	**43**
**Table S7** The calculated formation energies of all the cocrystals	**44**

**Figures S65–S75** Heteromolecular non-covalent bonded dimers used for the calculation of interaction energy (given in parentheses) from optimized geometry of cocrystals.	**45**
**Figures S76–S85** Homomolecular non-covalent bonded dimer used for the calculation of interaction energy (given in parentheses) from optimized geometry of cocrystals.	**60**

Mechanochemical experiments

Cocrystal screening was performed by liquid-assisted grinding (LAG) of mixture of corresponding donor and acceptor in stoichiometric ratios 1:1 and 2:1. All experiments were performed in a 5 mL stainless steel jar along with 20 µL of acetone and two stainless steel ball 5 mm in diameter. In the cases where new phases of 2:1 stoichiometry were not formed, additional experiments using acetonitrile, ethanol and nitromethane were performed. The reaction mixtures were then milled for 30 minutes in a Retsch MM200 Shaker Mill operating at 25 Hz. Products were characterized by PXRD and diffractograms were compared with starting components as well as with calculated PXRD patterns. Details on mechanochemical experiments are shown in **Table S1** and overlapped PXRD patterns are shown in **Figures S21–S32**.

Table S1 Mechanochemical experiments performed with donors and acceptors

donor	acceptor	stoichiometric ratio of donor : acceptor	*m*(donor)/ mg	*m*(acceptor)/ mg	liquid	PXRD
**btfa**	**bpy**	1 : 1	48.8	31.2	acetone	(**btfa**)(**bpy**)
		2:1	60.6	19.4	acetone	(**btfa**)$_2$(**bpy**)
**btfa**	**bpean**	1 : 1	45.6	34.4	acetone	(**btfa**)(**bpean**)
		2:1	58.1	21.9	acetone	(**btfa**)(**bpean**) + **btfa**
					acetonitrile	(**btfa**)(**bpean**) + **btfa**
					ethanol	(**btfa**)(**bpean**) + **btfa**
					nitromethane	(**btfa**)(**bpean**) + **btfa**
**btfa**	**dabco**	1 : 1	54.8	25.2	acetone	(**btfa**)(**dabco**)
		2:1	65.0	15.0	acetone	(**btfa**)$_2$(**dabco**)
**itfa**	**bpy**	1 : 1	52.1	27.9	acetone	(**itfa**)(**bpy**)
		2:1	63.1	16.9	acetone	(**itfa**)(**bpy**) + **itfa**
					acetonitrile	(**itfa**)(**bpy**) + **itfa**
					ethanol	(**itfa**)$_2$(**bpy**)
					nitromethane*	(**itfa**)$_2$(**bpy**)
**itfa**	**bpean**	1 : 1	49.0	31.0	acetone	(**itfa**)(**bpean**)

		2 : 1	60.8	19.2	acetone	(**itfa**)$_2$(**bpean**)
**itfa**	**dabco**	1 : 1	57.7	22.3	acetone	(**itfa**)(**dabco**)
		2 : 1	67.1	12.9	acetone	(**itfa**)$_2$(**dabco**)

*the structure of (**itfa**)$_2$(**bpy**) was refined from PXRD data of the product obtained by nitromethane-assisted grinding of **itfa** and **bpy** in 2:1 stoichiometric ratio.

Crystallization experiments

Single crystals of herein reported anilines and cocrystals were prepared by crystallization from solution. Single crystals of **btfa** were prepared by crystallization of 50 mg of commercial **btfa** from 5 mL of hot acetone, while single crystals of **itfa** were prepared by crystallization of 50 mg of synthesized **itfa** from 5 mL of hot ethanol. The 30 mg mixtures of donors and acceptors in 1:1 and 2:1 stoichiometric ratio were dissolved in 2.0 mL of hot solvent and left to crystallize at room temperature. For obtaining single crystals of (**btfa**)$_2$(**bpy**), (**itfa**)$_2$(**bpean**) and (**itfa**)$_2$(**dabco**), to the hot solution of donor and acceptor, a small amount of product obtained by liquid-assisted grinding was added (seeding). Crystal and molecular structure of prepared single crystals was determined by SCXRD. Details on crystallization experiments are given in **Table S2** Crystal data and refinement details are given in **Tables S3** and **S4** and ORTEP representations are given in **Figures S1–S12**.

Table S2 Crystallization experiments performed with donors and acceptors.

donor	acceptor	stoichiometric ratio of donor : acceptor	*m*(donor)/ mg	*m*(acceptor)/ mg	solvent	SCXRD
**btfa**	**bpy**	1 : 1	18.3	11.7	acetone	(**btfa**)(**bpy**)
		2 : 1	22.7	7.3	acetone*	(**btfa**)$_2$(**bpy**)
**btfa**	**bpean**	1 : 1	17.1	12.9	acetone	(**btfa**)(**bpean**)
		2 : 1	21.8	8.2	**	(**btfa**)(**bpean**) + **btfa**
**btfa**	**dabco**	1 : 1	20.6	9.4	nitromethane	(**btfa**)(**dabco**)
		2 : 1	24.4	5.6	acetone	(**btfa**)$_2$(**dabco**)
**itfa**	**bpy**	1 : 1	19.5	10.5	acetone	(**itfa**)(**bpy**)
		2 : 1	23.6	6.3	**	(**itfa**)(**bpy**) + **itfa**
**itfa**	**bpean**	1 : 1	18.4	11.6	acetone	(**itfa**)(**bpean**)
		2 : 1	22.8	7.2	acetone*	(**itfa**)$_2$(**bpean**)
**itfa**	**dabco**	1 : 1	21.7	8.3	acetone	(**itfa**)(**dabco**)
		2 : 1	25.2	4.8	ethanol*	(**itfa**)$_2$(**dabco**)

*seeding experiments **used solvents: acetone, acetonitrile, methanol, ethanol, nitromethane, dichloromethane, ethyl acetate, 2,2,2-trifluoroethanol, tetrahydrofuran, toluene and pentan-2-one, with and without seeding

Table S3 Crystal data and refinement details for **btfa** and cocrystals with **btfa**.

	**btfa**	(**btfa**)(**bpy**)	(**btfa**)$_2$(**bpy**)
CCDC Number	2336067	2336068	2336069
Molecular formula	$C_6H_2BrF_4N$	$C_{16}H_{10}BrF_4N_3$	$C_{22}H_{12}Br_2F_8N_4$
M_r	244.00	400.18	644.18
Crystal system	monoclinic	orthorhombic	triclinic
Space group	$C2/c$	$Pca2_1$	$P\bar{1}$
Crystal data:			
a / Å	7.5048(6)	34.2778(16)	8.3031(2)
b / Å	14.8073(8)	5.6737(3)	12.8784(3)
c / Å	6.7915(6)	7.5806(4)	22.7435(7)
α / °	90	90	99.821(2)
β / °	112.519(9)	90	97.897(2)
γ / °	90	90	107.026(2)
V / Å^3	697.17(10)	1474.29(13)	2245.56(11)
Z	4	4	4
D_{calc} / g cm^{-3}	2.325	1.803	1.905
λ(MoK_α) / Å	0.71073	0.71073	0.71073
T / K	170	170	170
μ / mm^{-1}	5.907	2.837	3.696
F(000)	464	792	1256
Refl. collected/unique	4971 / 1022	12469 / 2959	33672 / 8816
Parameters	61	223	673
$\Delta\rho_{max}$, $\Delta\rho_{min}$ / e Å^{-3}	0.972; –0.605	0.344; –0.511	0.890; –0.437
$R[F^2 > 4\sigma(F^2)]$	0.0286	0.0407	0.0494
w$R(F^2)$	0.0709	0.0927	0.1143
Goodness-of-fit, S	1.037	1.068	1.025

Table S3 Continued.

	(**btfa**)(**bpean**)	(**btfa**)(**dabco**)	(**btfa**)$_2$(**dabco**)
CCDC Number	2336070	2336071	2336072
Molecular formula	$C_{18}H_{14}BrF_4N_3$	$C_{12}H_{14}BrF_4N_3$	$C_{18}H_{16}Br_2F_8N_4$
M_r	428.23	356.16	600.17
Crystal system	monoclinic	monoclinic	monoclinic
Space group	$P2_1/n$	$C2/c$	$P2_1/c$
Crystal data:			
a / Å	7.7617(4)	19.808(4)	16.0886(4)
b / Å	12.1671(5)	16.6932(10)	12.8605(3)
c / Å	18.1685(9)	12.870(3)	10.1150(3)
α / °	90	90	90
β / °	101.622(5)	137.53(4)	91.060(2)
γ / °	90	90	90
V / Å^3	1680.61(14)	2873.2(16)	2092.50(9)
Z	4	8	4
D_{calc} / g cm^{-3}	1.692	1.591	1.905
$\lambda(MoK_\alpha)$ / Å	0.71073	0.71073	0.71073
T / K	170	170	170
μ / mm^{-1}	2.494	2.897	3.958
F(000)	856	1328	1176
Refl. collected/unique	26093 / 3304	13049 / 2680	20552 / 5048
Parameters	241	259	301
$\Delta\rho_{max}$, $\Delta\rho_{min}$ / e Å^{-3}	0.654; –0.475	0.372; –0.440	0.576; –0.383
$R[F^2 > 4\sigma(F^2)]$	0.0441	0.0456	0.0288
w$R(F^2)$	0.1175	0.1238	0.0769
Goodness-of-fit, S	1.053	1.034	1.036

Table S4 Crystal data and refinement details for **itfa** and cocrystals with **itfa**.

	**itfa**	**(itfa)(bpy)**	**(itfa)(bpean)**
CCDC Number	2336073	2336074	2336076
Molecular formula	$C_6H_2IF_4N$	$C_{16}H_{10}F_4IN_3$	$C_{18}H_{14}F_4IN_3$
M_r	290.99	447.17	475.22
Crystal system	orthorhombic	monoclinic	monoclinic
Space group	*Pnma*	$P2_1/c$	$P2_1/c$
Crystal data:			
a / Å	13.7777(7)	16.2048(9)	8.4653(3)
b / Å	10.3824(5)	12.8059(6)	5.6127(3)
c / Å	5.2495(2)	7.5273(3)	37.7384(13)
α / °	90	90	90
β / °	90	92.174(4)	90.984(3)
γ / °	90	90	90
V / Å^3	750.92(6)	1560.92(13)	1792.81(13)
Z	4	4	4
D_{calc} / g cm^{-3}	2.574	1.903	1.761
λ(MoK_α) / Å	0.71073	0.71073	0.71073
T / K	170	170	170
μ / mm^{-1}	4.275	2.097	1.832
F(000)	536	864	928
Refl. collected/unique	1328 / 1115	19546 / 3942	15123 / 4767
Parameters	65	223	242
$\Delta\rho_{max}$, $\Delta\rho_{min}$ / e Å^{-3}	0.672; –0.659	1.769; –1.346	1.852; –1.400
$R[F^2 > 4\sigma(F^2)]$	0.0282	0.0588	0.0632
w$R(F^2)$	0.0596	0.1542	0.1806
Goodness-of-fit, S	1.070	1.072	1.088

Table S4 Continued.

	(**itfa**)$_2$(**bpean**)	(**itfa**)(**dabco**)	(**itfa**)$_2$(**dabco**)
CCDC Number	2336077	2336078	2336079
Molecular formula	$C_{24}H_{16}F_8I_2N_4$	$C_{12}H_{14}F_4IN_3$	$C_{18}H_{16}F_8I_2N_4$
M_r	766.21	403.16	694.15
Crystal system	monoclinic	monoclinic	triclinic
Space group	$P2_1/c$	$P2_1/c$	$P\bar{1}$
Crystal data:			
a / Å	9.9032(5)	9.8812(3)	9.6372(2)
b / Å	7.3995(3)	12.1506(3)	10.1087(2)
c / Å	16.9831(8)	12.3154(3)	12.3837(3)
α / °	90	90	111.205(2)
β / °	95.720(4)	106.759(3)	94.697(2)
γ / °	90	90	104.164(2)
V / Å^3	1238.30(10)	1415.81(7)	1070.99(4)
Z	2	4	2
D_{calc} / g cm^{-3}	2.055	1.891	2.153
λ(MoK_α) / Å	0.71073	0.71073	0.71073
T / K	170	170	170
μ / mm^{-1}	2.622	2.300	3.019
F(000)	732	784	660
Refl. collected/unique	12586 / 3291	27939 / 4125	38736 / 6258
Parameters	178	187	301
$\Delta\rho_{max}$, $\Delta\rho_{min}$ / e Å^{-3}	2.334; –1.861	0.997; –1.017	0.418; –0.878
$R[F^2 > 4\sigma(F^2)]$	0.0508	0.0278	0.0205
w$R(F^2)$	0.1326	0.0757	0.0485
Goodness-of-fit, S	1.006	1.080	1.043

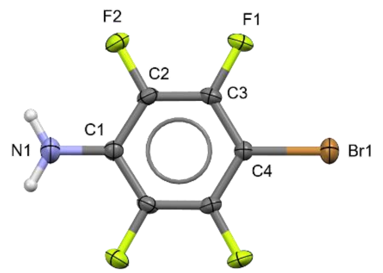

Figure S1 Molecular structure of **btfa** showing the atom-labelling scheme for the asymmetric unit. Displacement ellipsoids are drawn at the 50 % probability level, and H atoms are shown as small spheres of arbitrary radius.

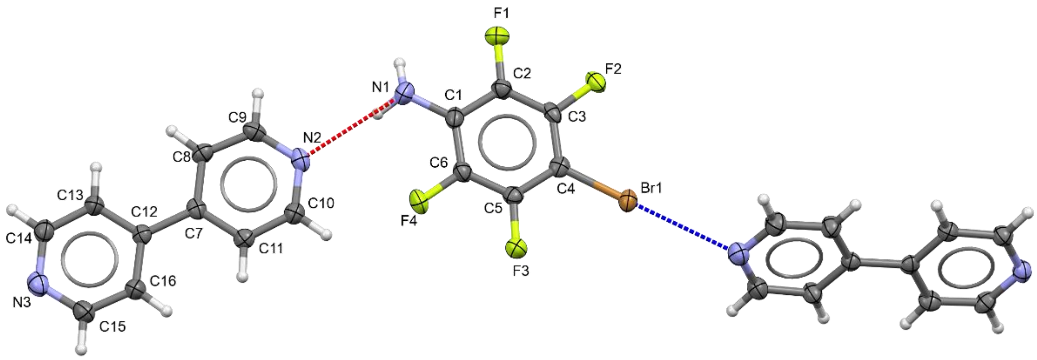

Figure S2 Molecular structure of (**btfa**)(**bpy**) showing the atom-labelling scheme for the asymmetric unit. Displacement ellipsoids are drawn at the 50 % probability level, and H atoms are shown as small spheres of arbitrary radius.

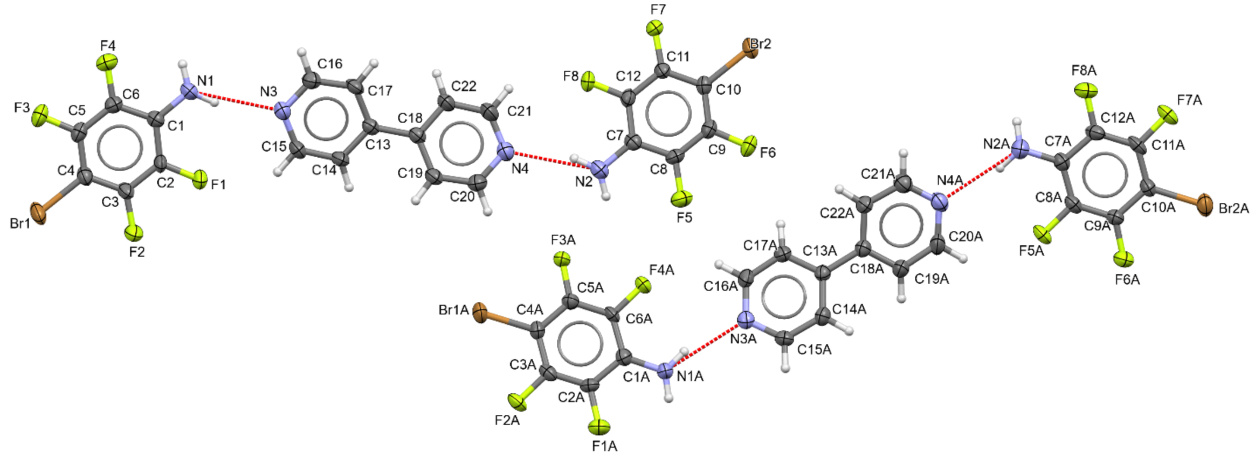

Figure S3 Molecular structure of (**btfa**)$_2$(**bpy**) showing the atom-labelling scheme for the asymmetric unit. Displacement ellipsoids are drawn at the 50 % probability level, and H atoms are shown as small spheres of arbitrary radius.

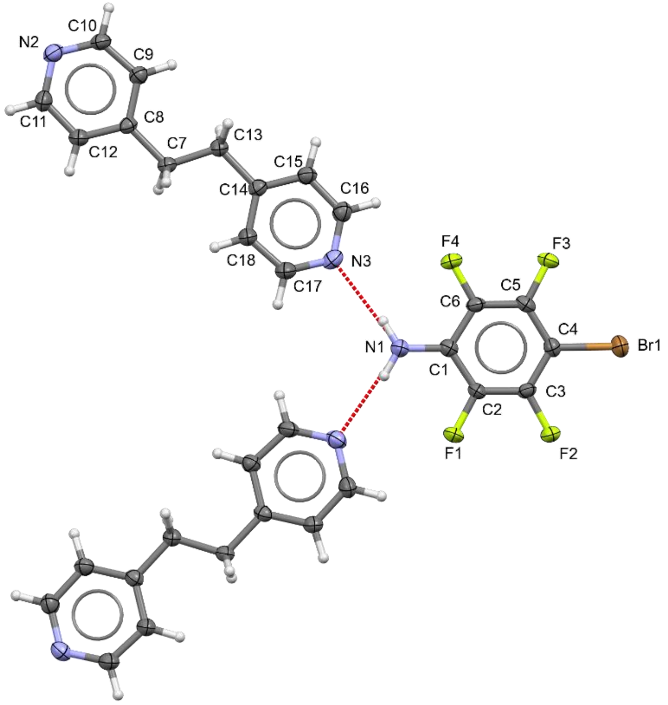

Figure S4 Molecular structure of (**btfa**)(**bpean**) showing the atom-labelling scheme for the asymmetric unit. Displacement ellipsoids are drawn at the 50 % probability level, and H atoms are shown as small spheres of arbitrary radius.

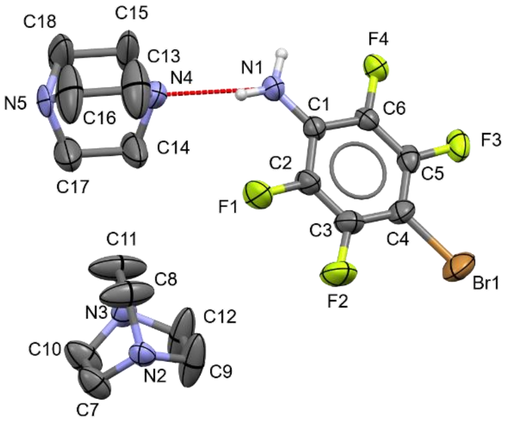

Figure S5 Molecular structure of (**btfa**)(**dabco**) showing the atom-labelling scheme for the asymmetric unit. Displacement ellipsoids are drawn at the 50 % probability level.

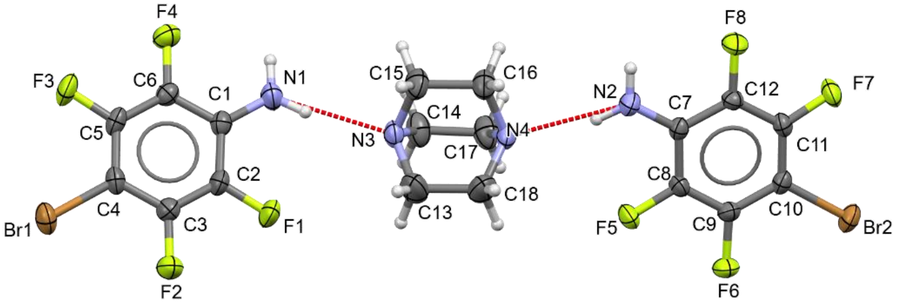

Figure S6 Molecular structure of (**btfa**)$_2$(**dabco**) showing the atom-labelling scheme for the asymmetric unit. Displacement ellipsoids are drawn at the 50 % probability level, and H atoms are shown as small spheres of arbitrary radius.

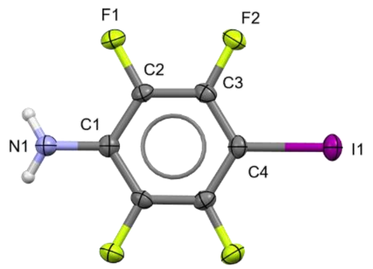

Figure S7 Molecular structure of **itfa** showing the atom-labelling scheme for the asymmetric unit. Displacement ellipsoids are drawn at the 50 % probability level, and H atoms are shown as small spheres of arbitrary radius.

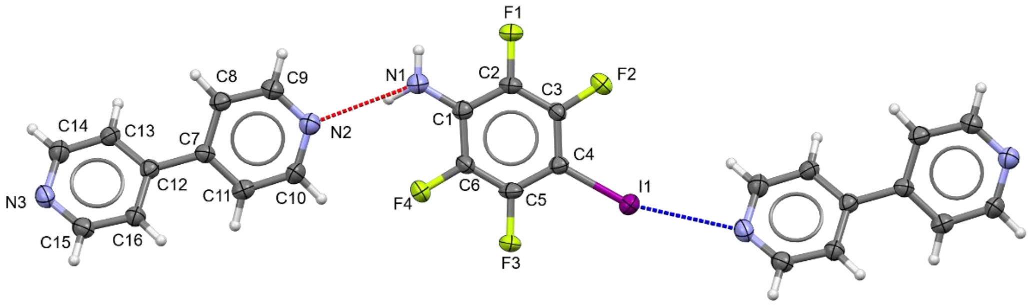

Figure S8 Molecular structure of (**itfa**)(**bpy**) showing the atom-labelling scheme for the asymmetric unit. Displacement ellipsoids are drawn at the 50 % probability level, and H atoms are shown as small spheres of arbitrary radius.

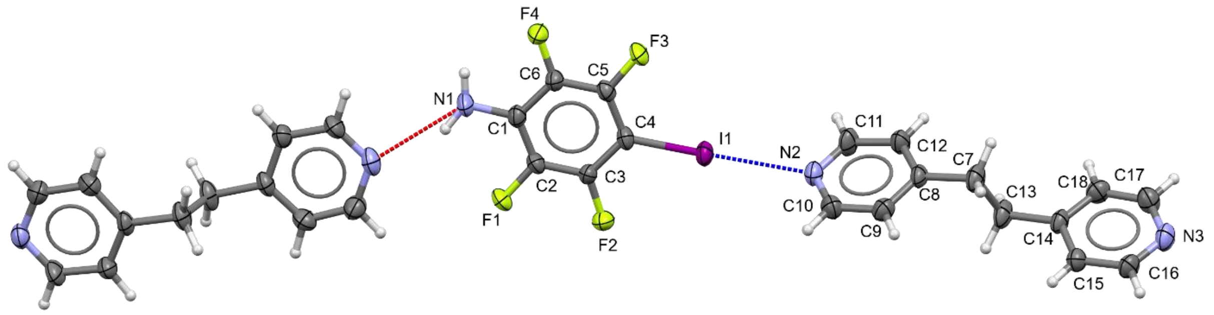

Figure S9 Molecular structure of (**itfa**)(**bpean**) showing the atom-labelling scheme for the asymmetric unit. Displacement ellipsoids are drawn at the 50 % probability level, and H atoms are shown as small spheres of arbitrary radius.

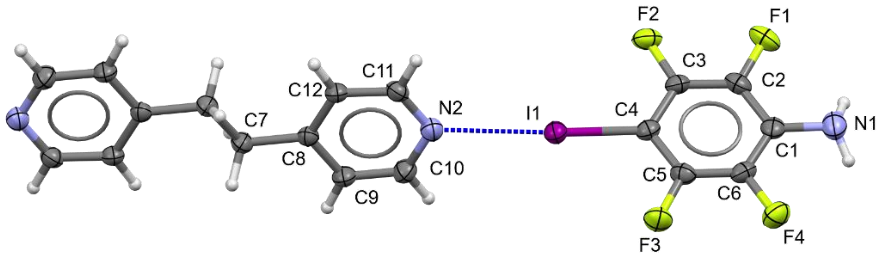

Figure S10 Molecular structure of (**itfa**)$_2$(**bpean**) showing the atom-labelling scheme for the asymmetric unit. Displacement ellipsoids are drawn at the 50 % probability level, and H atoms are shown as small spheres of arbitrary radius.

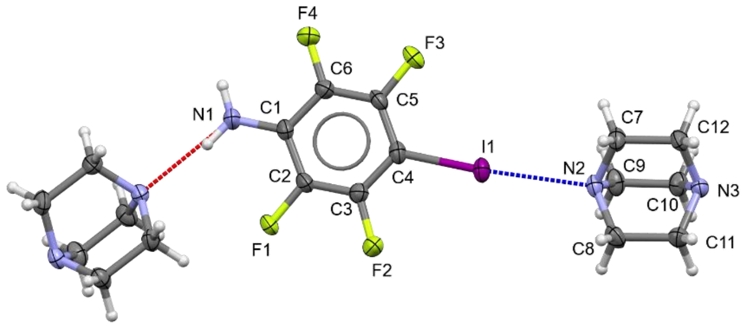

Figure S11 Molecular structure of (**itfa**)(**dabco**) showing the atom-labelling scheme for the asymmetric unit. Displacement ellipsoids are drawn at the 50 % probability level, and H atoms are shown as small spheres of arbitrary radius.

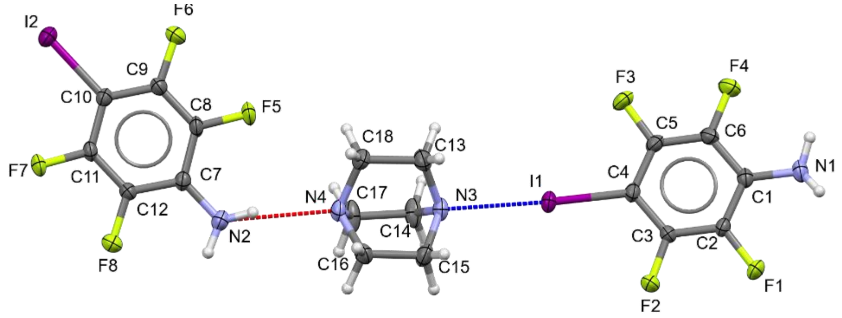

Figure S12 Molecular structure of (**itfa**)$_2$(**dabco**) showing the atom-labelling scheme for the asymmetric unit. Displacement ellipsoids are drawn at the 50 % probability level, and H atoms are shown as small spheres of arbitrary radius.

Table S5 Experimental and crystallographic data for (**itfa**)$_2$(**bpy**) (refined from PXRD data).

Compound	(**itfa**)$_2$(**bpy**)
CCDC Number	2336075
M_r	369.08
Empirical formula	$C_{11}H_6F_4IN_2$
Crystal system	monoclinic
Space group	$P2_1/a$
a / Å	15.6355(4)
b / Å	7.50526(18)
c / Å	10.0428(2)
α /°	90
β / °	89.665(2)
γ / °	90
V / Å^3	1178.51(5)
Z	4
ρ_{calc} / g cm^{-3}	2.080
μ / mm^{-1}	2.751
Radiation	Mo Kα
2Θ range / °	2.000 to 55.000
Data / parameters	7400/33
Goodness–of–fit, χ^a	4.14
Final Rp and Rwp^b values/%	2.92, 4.24

[a] $\chi = \sqrt{\frac{\sum_{i=1}^{N} w_i\left(y_{obs,i}-y_{calc,i}(\mathbf{p})\right)^2}{N-P}}$, where w_i is weight (herein equal to $\sqrt{y_{obs,i}}$), $y_{obs,i}$ is the i-th observed intensity, y_{calc_i} i-th calculated intensity, **p** parameter vector, N number of observations and P number of parameters

[b] $R_p = \frac{\sum_{i=1}^{N}|y_{obs,i}-y_{calc,i}(\mathbf{p})|}{\sum_{i=1}^{N} y_{obs,i}}$, $R_{wp} = \sqrt{\frac{\sum_{i=1}^{N} w_i\left(y_{obs,i}-y_{calc,i}(\mathbf{p})\right)^2}{\sum_{i=1}^{N} w_i y_{obs,i}^2}}$

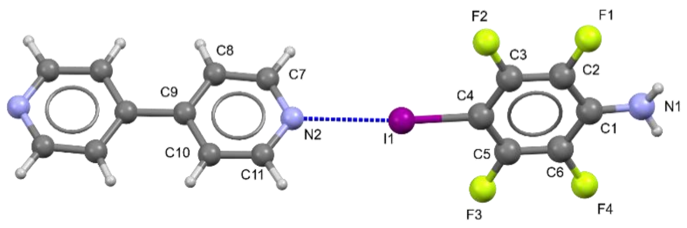

Figure S13 Molecular structure of (**itfa**)$_2$(**bpy**) showing the atom-labelling scheme for the asymmetric unit.

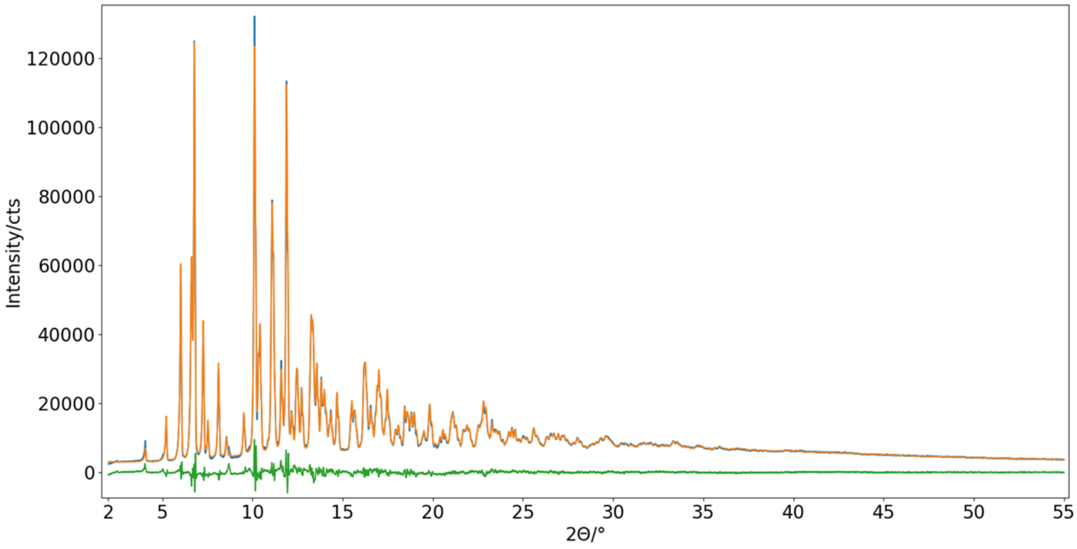

Figure S14 Whole-range final Rietveld refinement data. PXRD data for (**itfa**)$_2$(**bpy**) (blue), calculated pattern (orange) and difference between observed and calculated pattern (green).

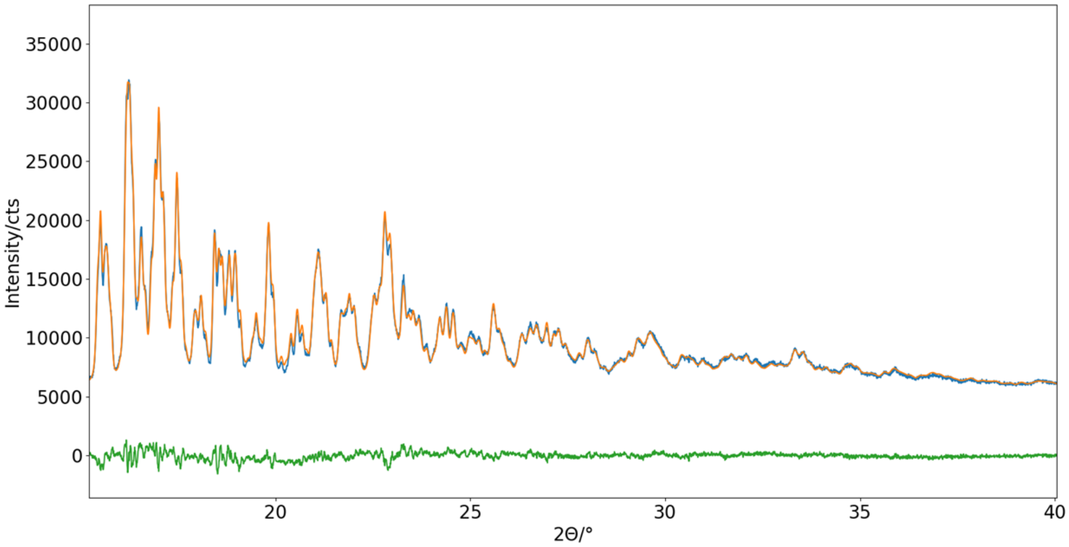

Figure S15 Medium-angle detail of final Rietveld refinement. PXRD data for (**itfa**)$_2$(**bpy**) (blue), calculated pattern (orange) and difference between observed and calculated pattern (green).

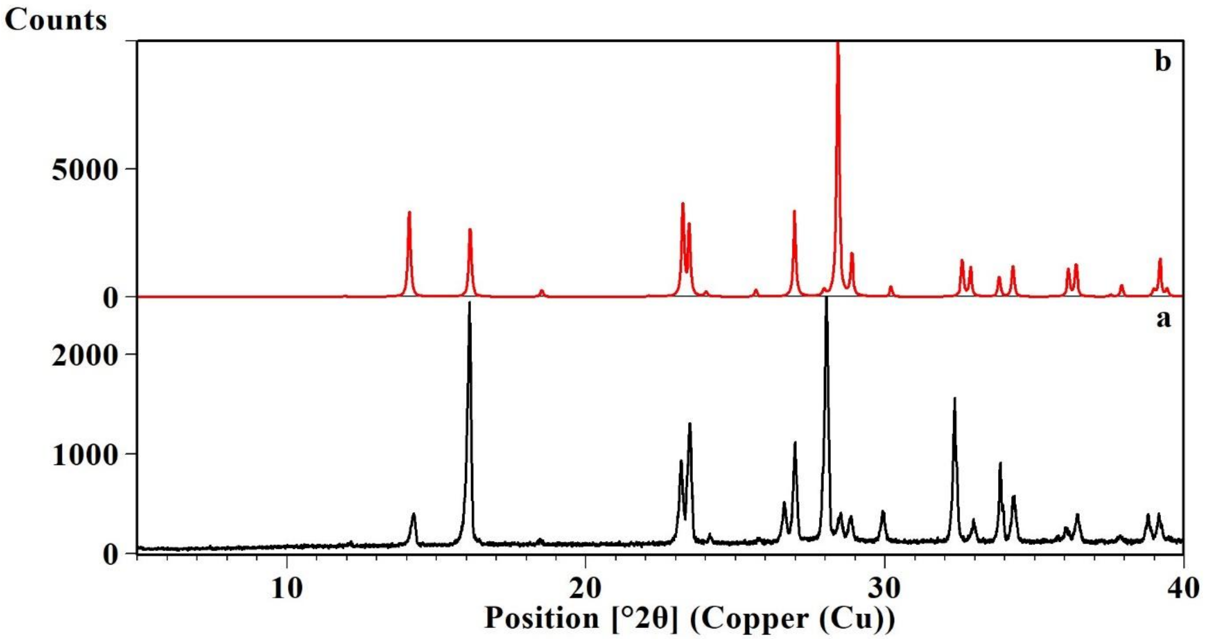

Figure S16 PXRD patterns of: a) **btfa** and b) calculated pattern from **btfa** single crystal data.

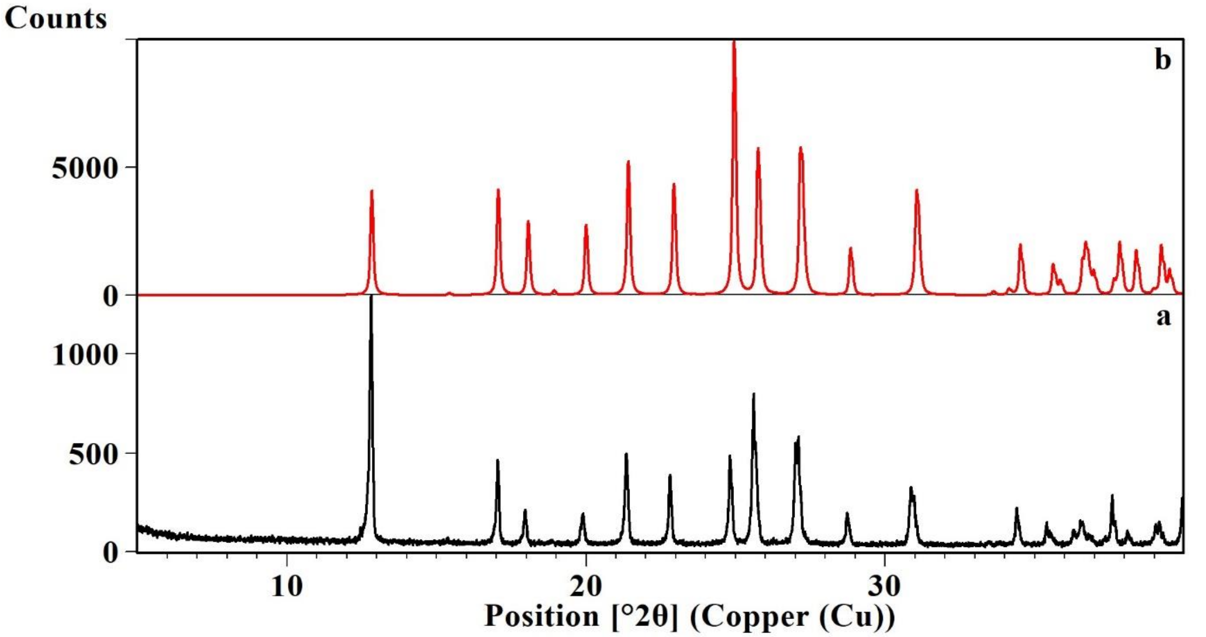

Figure S17 PXRD patterns of: a) **itfa** and b) calculated pattern from MARGUL single crystal data.

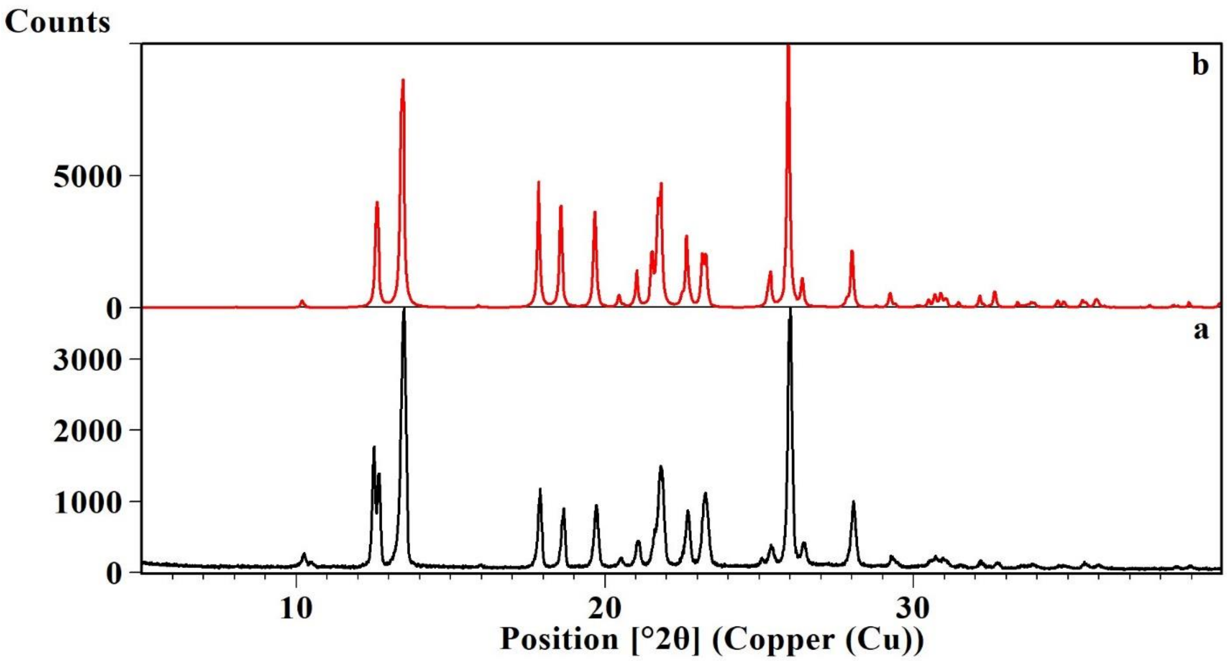

Figure S18 PXRD patterns of: a) **bpy** and b) calculated pattern from HIQWEJ01 single crystal data.

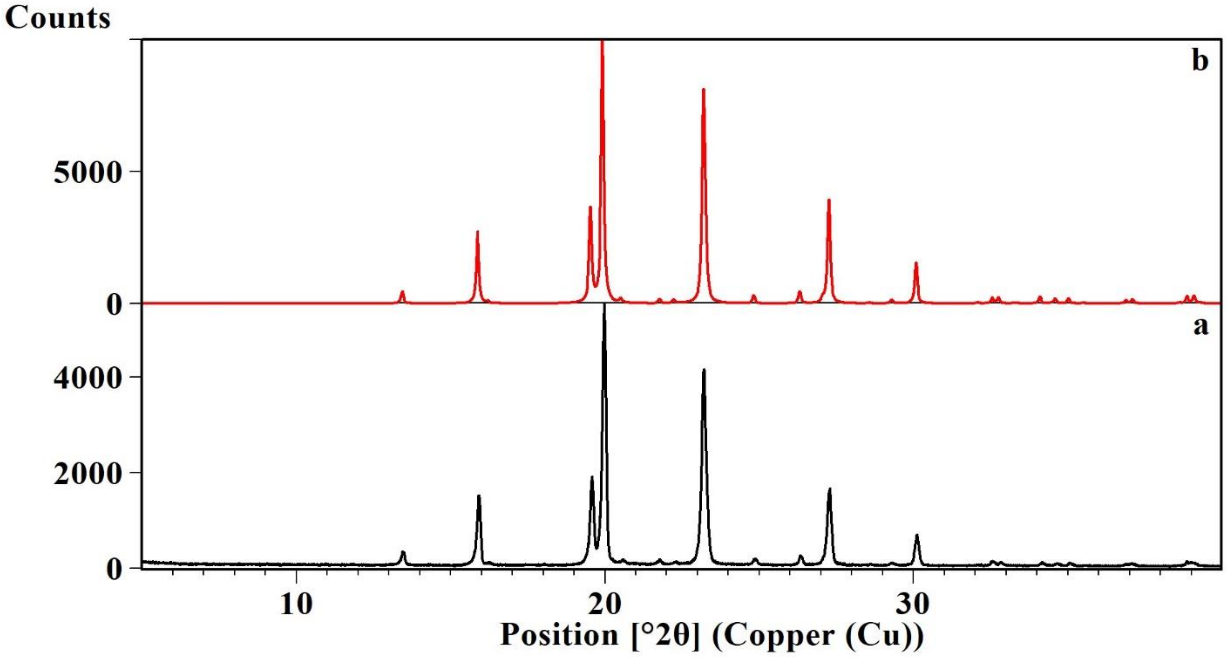

Figure S19 PXRD patterns of: a) **bpean** and b) calculated pattern from ZEXKIW single crystal data.

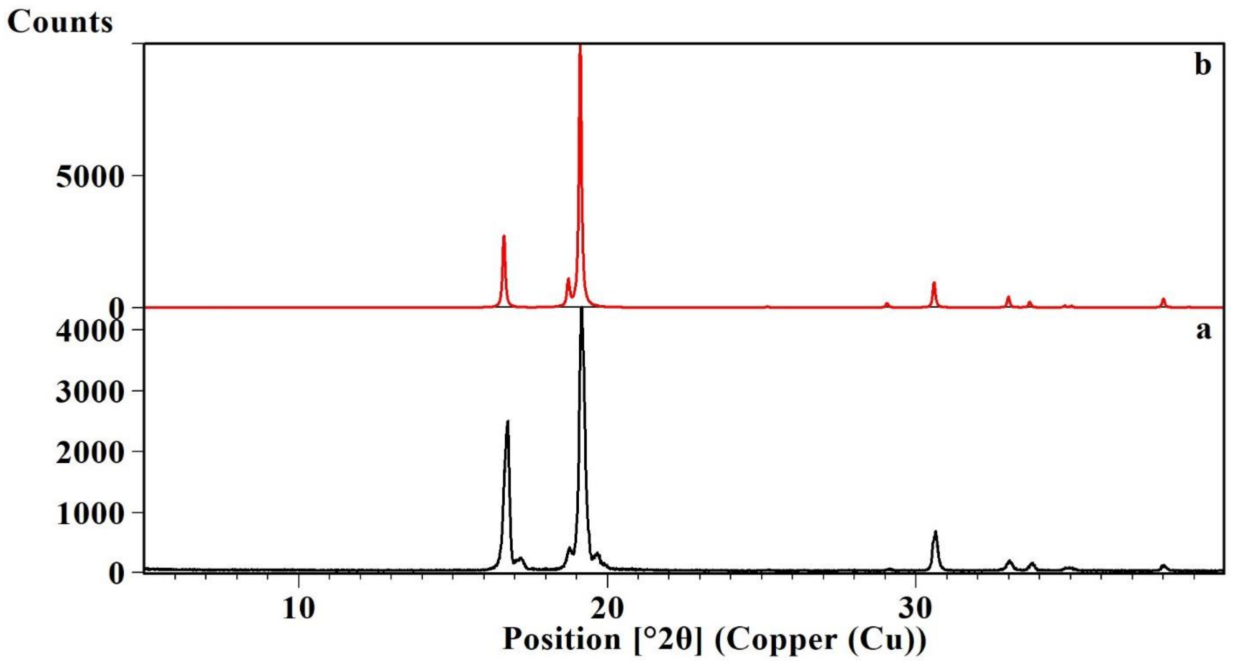

Figure S20 PXRD patterns of: a) **dabco** and b) calculated pattern from TETDAM01 single crystal data.

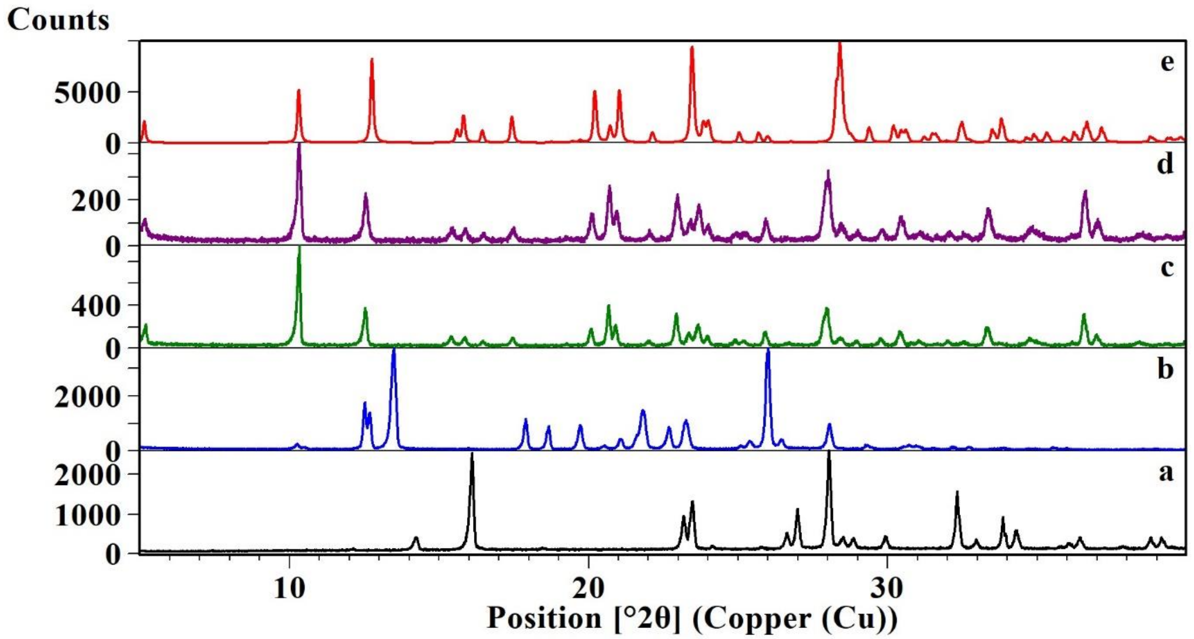

Figure S21 PXRD patterns of: a) **btfa**, b) **bpy**, c) **btfa** + **bpy** – LAG (acetone), 1:1, d) (**btfa**)(**bpy**) – crystallization from solution, e) calculated pattern from (**btfa**)(**bpy**) single crystal data.

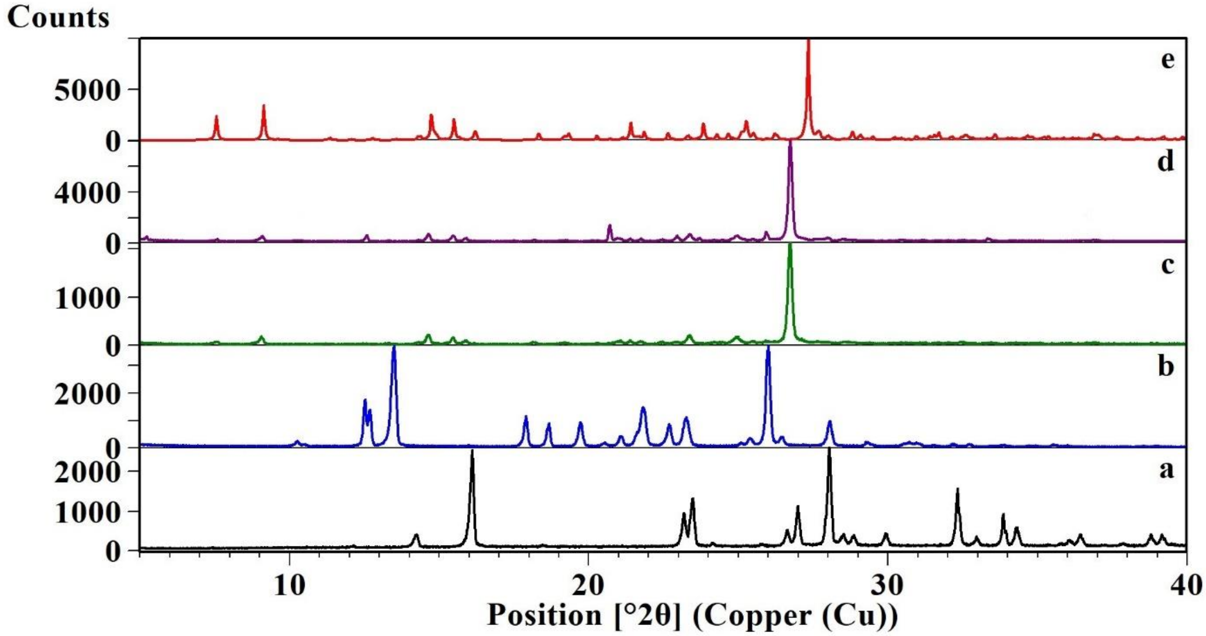

Figure S22 PXRD patterns of: a) **btfa**, b) **bpy**, c) **btfa** + **bpy** – LAG (acetone), 2:1, d) (**btfa**)$_2$(**bpy**) – crystallization from solution, e) calculated pattern from (**btfa**)$_2$(**bpy**) single crystal data.

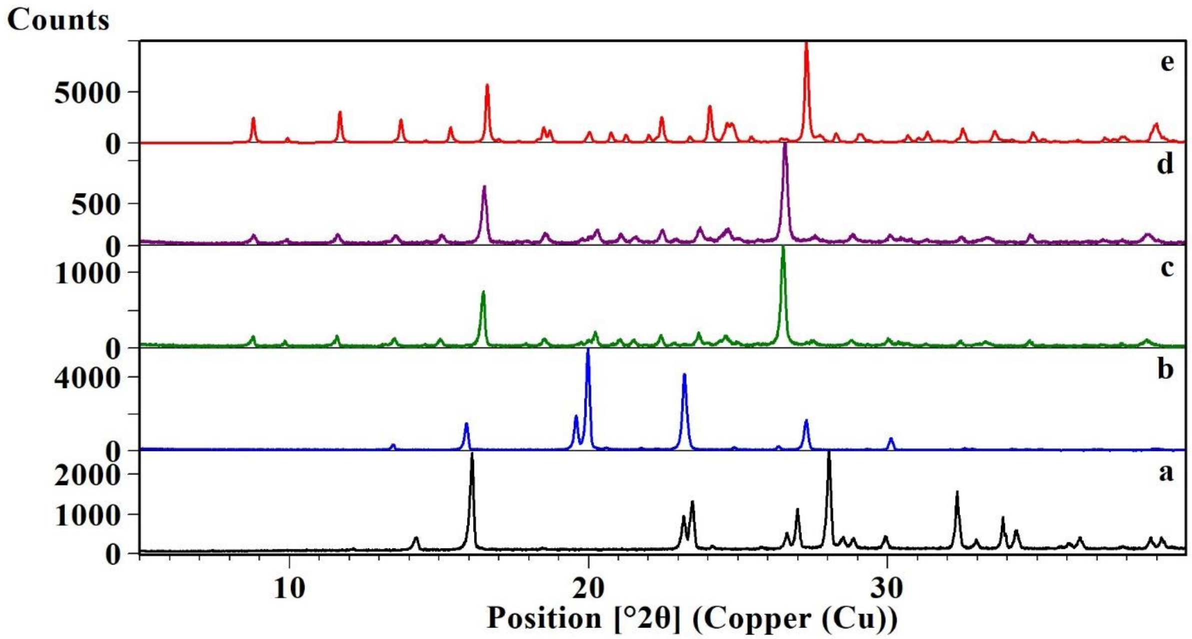

Figure S23 PXRD patterns of: a) **btfa**, b) **bpean**, c) **btfa** + **bpean** – LAG (acetone), 1:1, d) (**btfa**)(**bpean**) – crystallization from solution, e) calculated pattern from (**btfa**)(**bpean**) single crystal data.

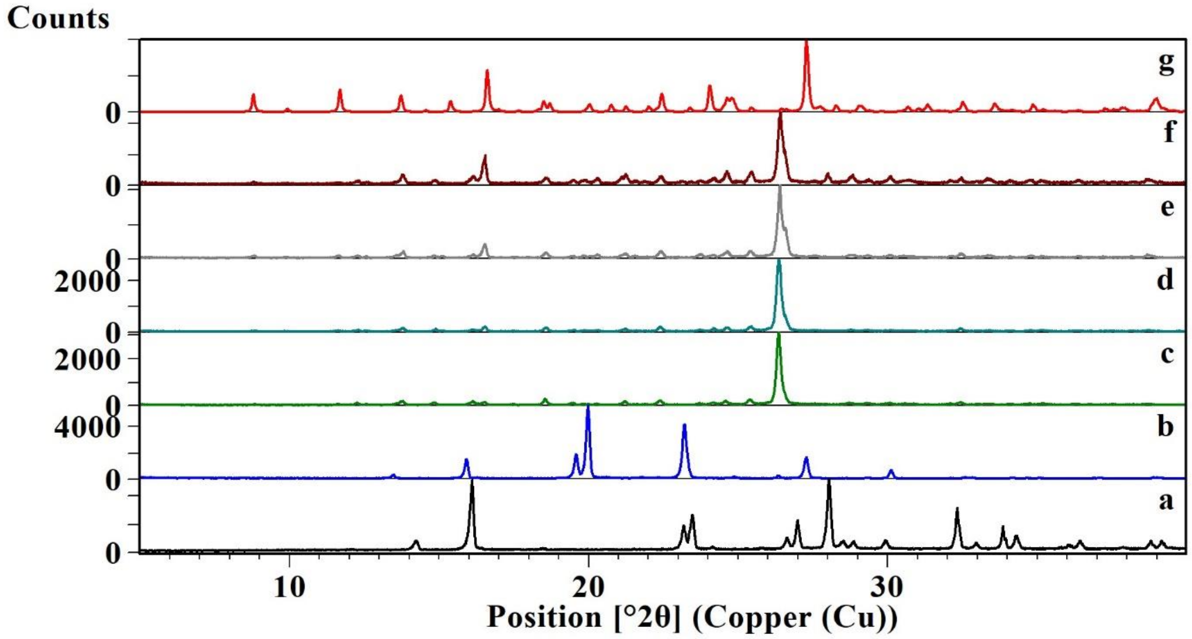

Figure S24 PXRD patterns of: a) **btfa**, b) **bpean**, c) **btfa** + **bpean** –LAG (acetone), 2:1, d) **btfa** + **bpean** – LAG (acetonitrile), 2:1, e) **btfa** + **bpean** –LAG (ethanol), 2:1, f) **btfa** + **bpean** – LAG (nitromethane), 2:1, g) calculated pattern from (**btfa**)(**bpean**) single crystal data.

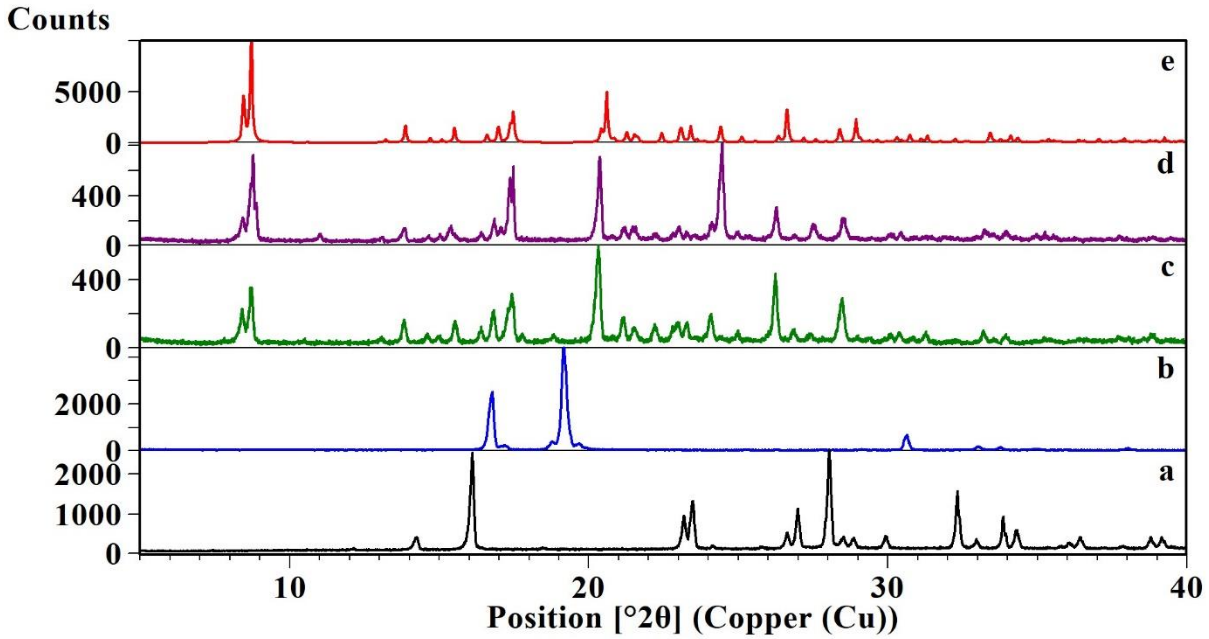

Figure S25 PXRD patterns of: a) **btfa**, b) **dabco**, c) **btfa** + **dabco** –LAG (acetone), 1:1, d) (**btfa**)(**dabco**) – crystallization from solution, e) calculated pattern from (**btfa**)(**dabco**) single crystal data.

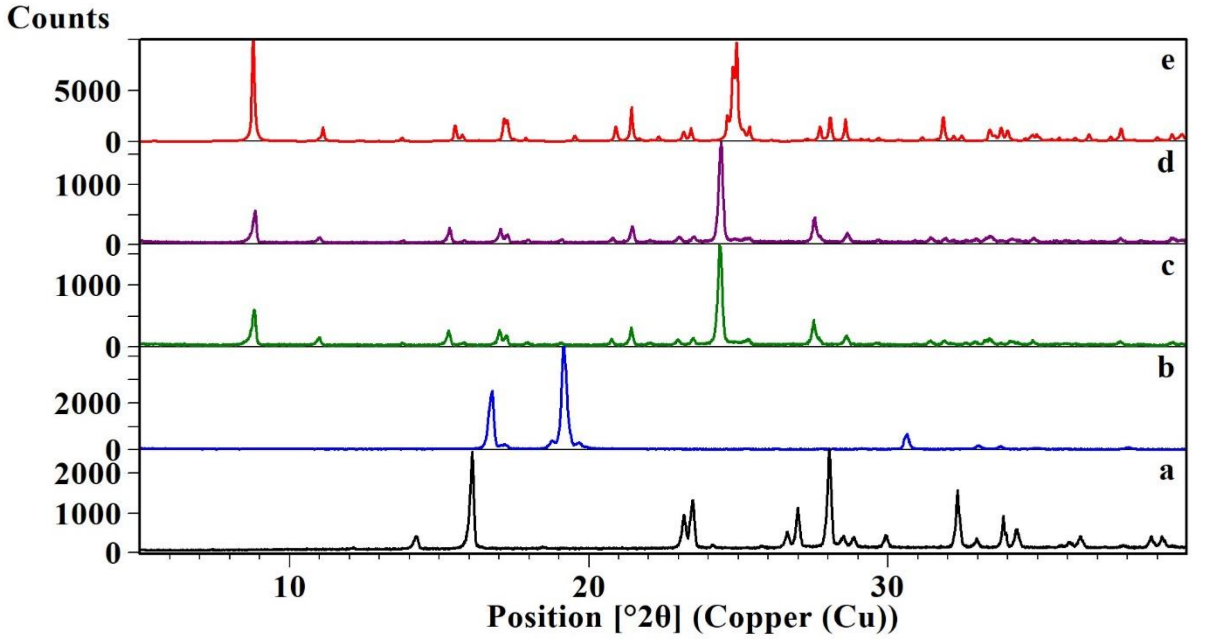

Figure S26 PXRD patterns of: a) **btfa**, b) **dabco**, c) **btfa** + **dabco** –LAG (acetone), 2:1, d) (**btfa**)$_2$(**dabco**) – crystallization from solution, e) calculated pattern from (**btfa**)$_2$(**dabco**) single crystal data.

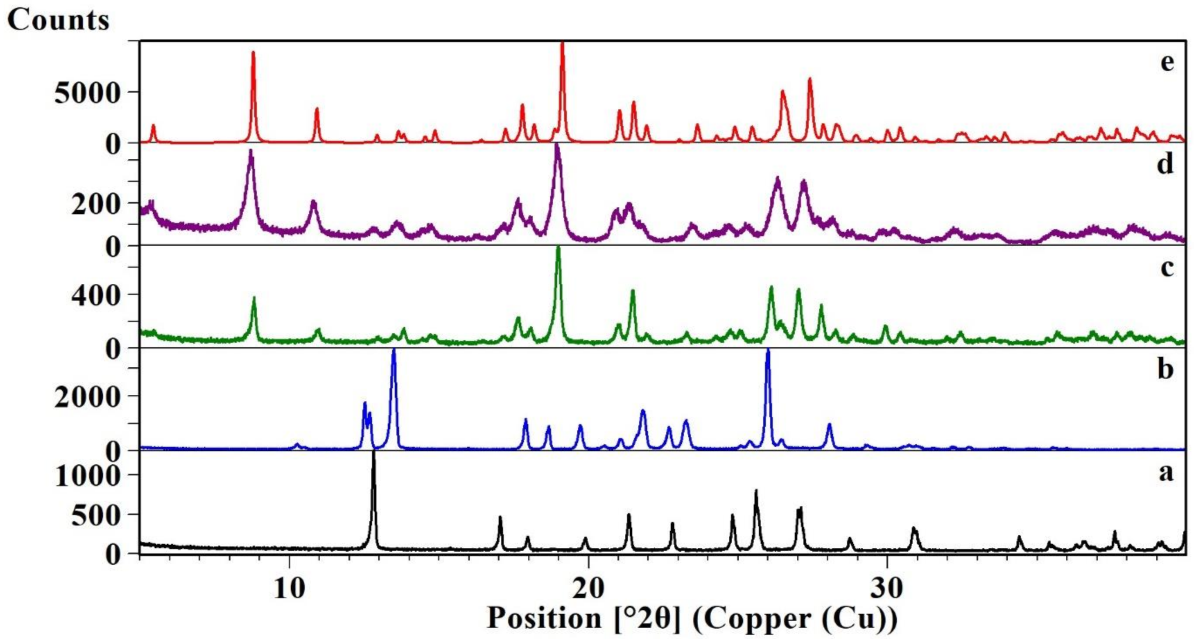

Figure S27 PXRD patterns of: a) **itfa**, b) **bpy**, c) **itfa** + **bpy** – LAG (ACT), 1:1, d) (**itfa**)(**bpy**) – crystallization from solution, e) calculated pattern from (**itfa**)(**bpy**) single crystal data.

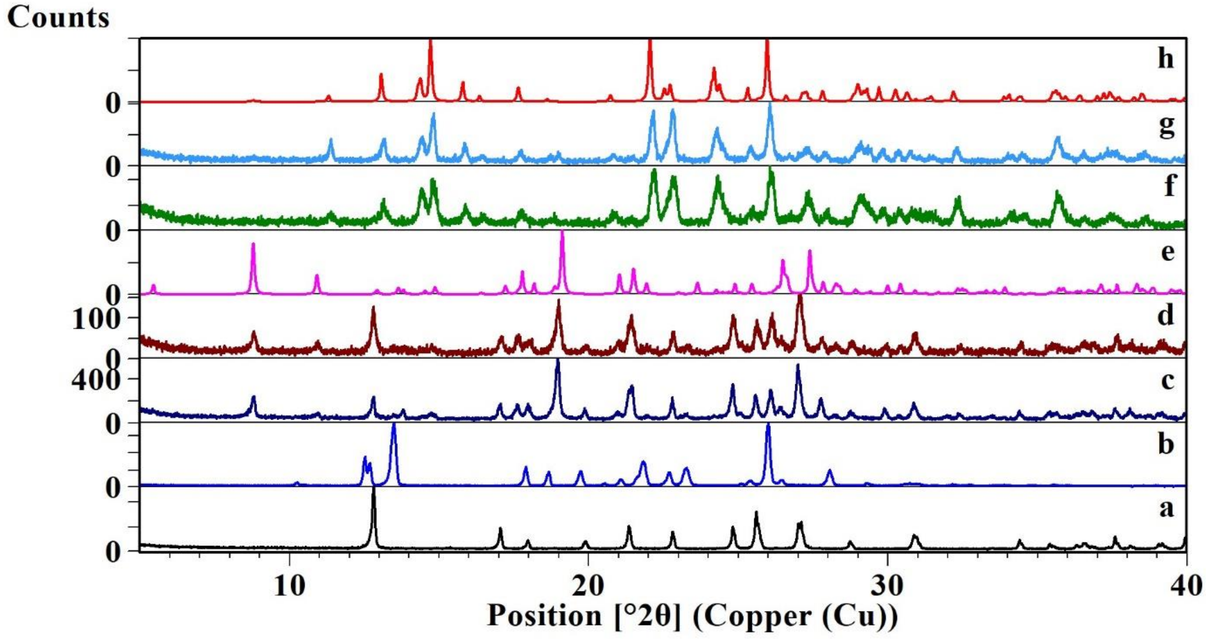

Figure S28 PXRD patterns of: a) **itfa**, b) **bpy**, c) **itfa** + **bpy** – LAG (acetone), 2:1, d) **itfa** + **bpy** – LAG (acetonitrile), 2:1, e) calculated pattern from (**itfa**)(**bpy**) single crystal data, f) **itfa** + **bpy** – LAG (ethanol), 2:1, g) **itfa** + **bpy** – LAG (nitromethane), 2:1, h) calculated pattern from (**itfa**)(**bpy**) powder data.

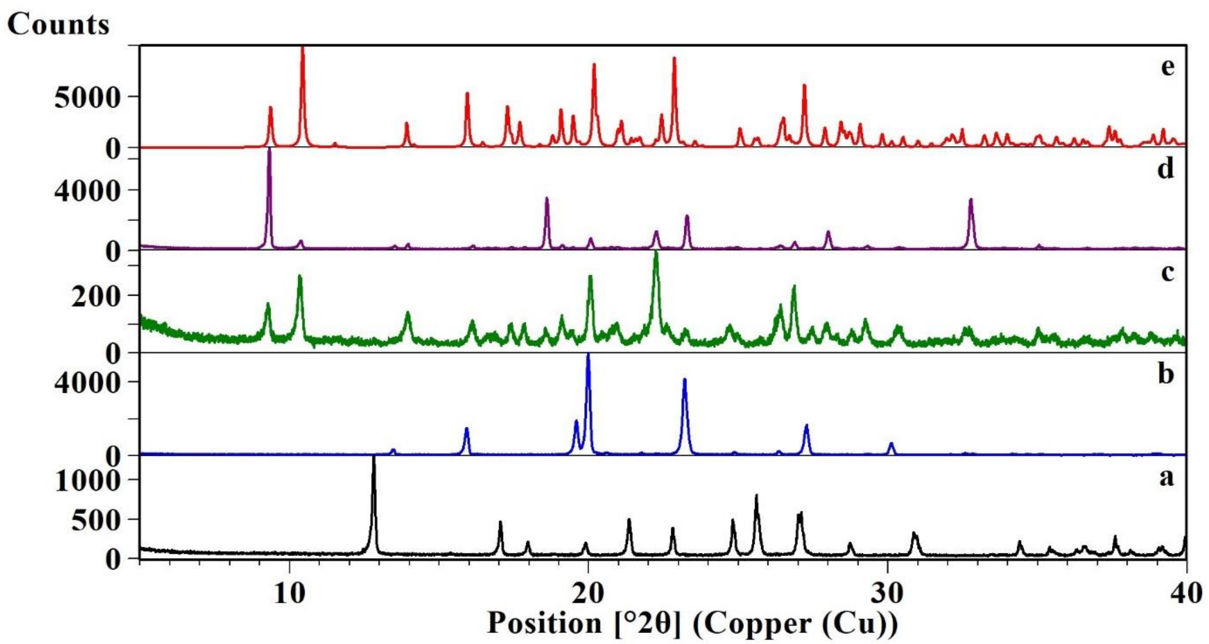

Figure S29 PXRD patterns of: a) **itfa**, b) **bpean**, c) **itfa** + **bpean** – LAG (acetone), 1:1, d) (**itfa**)(**bpean**) – crystallization from solution, e) calculated pattern from (**itfa**)(**bpean**) single crystal data.

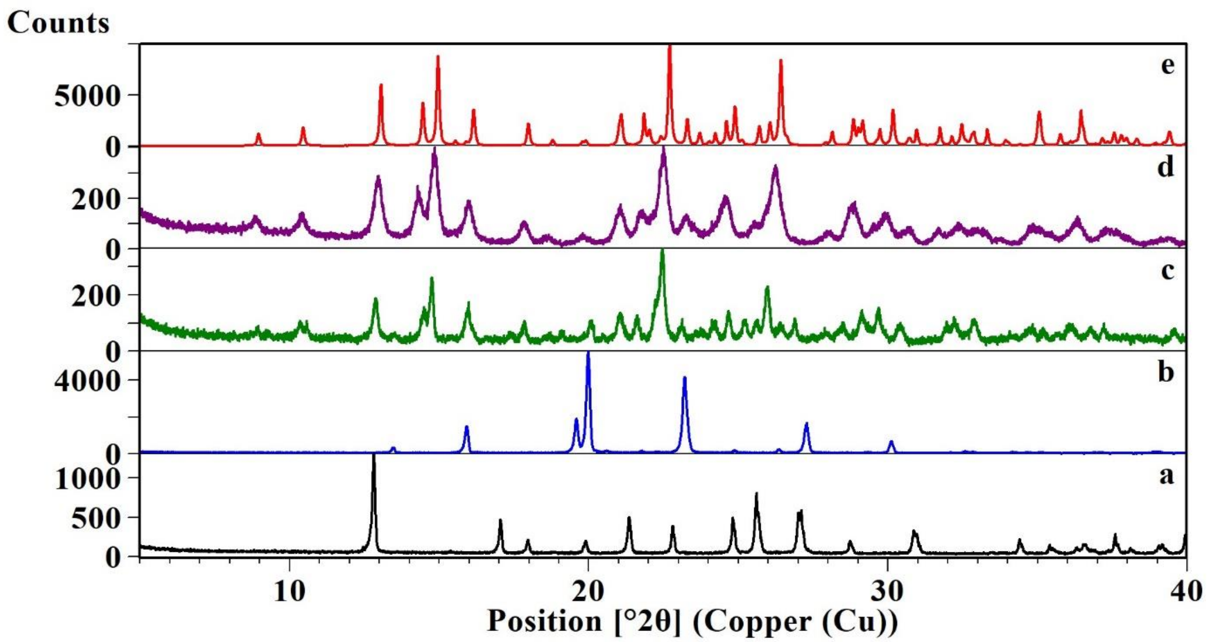

Figure S30 PXRD patterns of: a) **itfa**, b) **bpean**, c) **itfa** + **bpean** – LAG (acetone), 2:1, d) (**itfa**)$_2$(**bpean**) – crystallization from solution, e) calculated pattern from (**itfa**)$_2$(**bpean**) single crystal data.

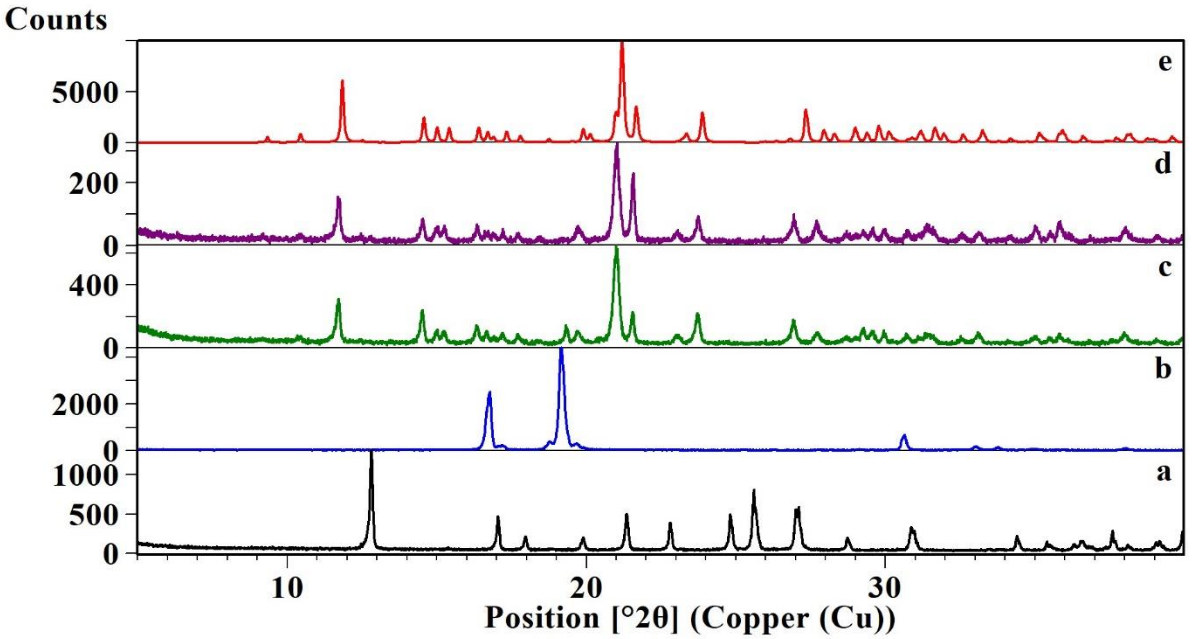

Figure S31 PXRD patterns of: a) **itfa**, b) **dabco**, c) **itfa** + **dabco** – LAG (acetone), 1:1, d) (**itfa**)(**dabco**) – crystallization from solution, e) calculated pattern from (**itfa**)(**dabco**) single crystal data.

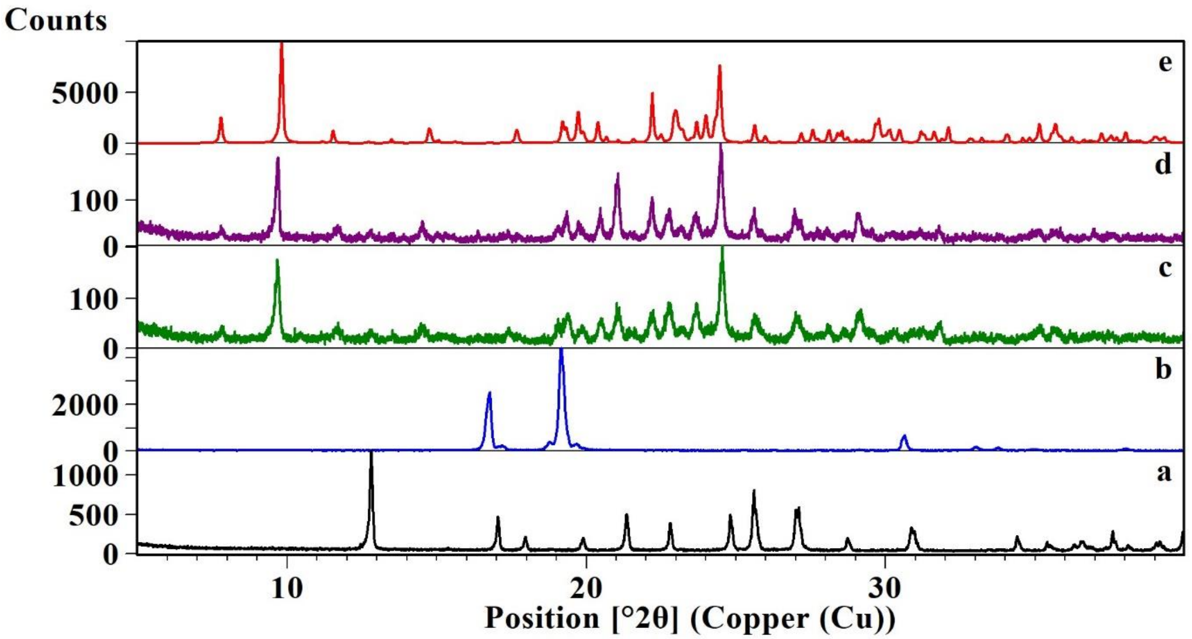

Figure S32 PXRD patterns of: a) **itfa**, b) **dabco**, c) **itfa** + **dabco** – LAG (acetone), 2:1, d) (**itfa**)$_2$(**dabco**) – crystallization from solution, e) calculated pattern from (**itfa**)$_2$(**dabco**) single crystal data.

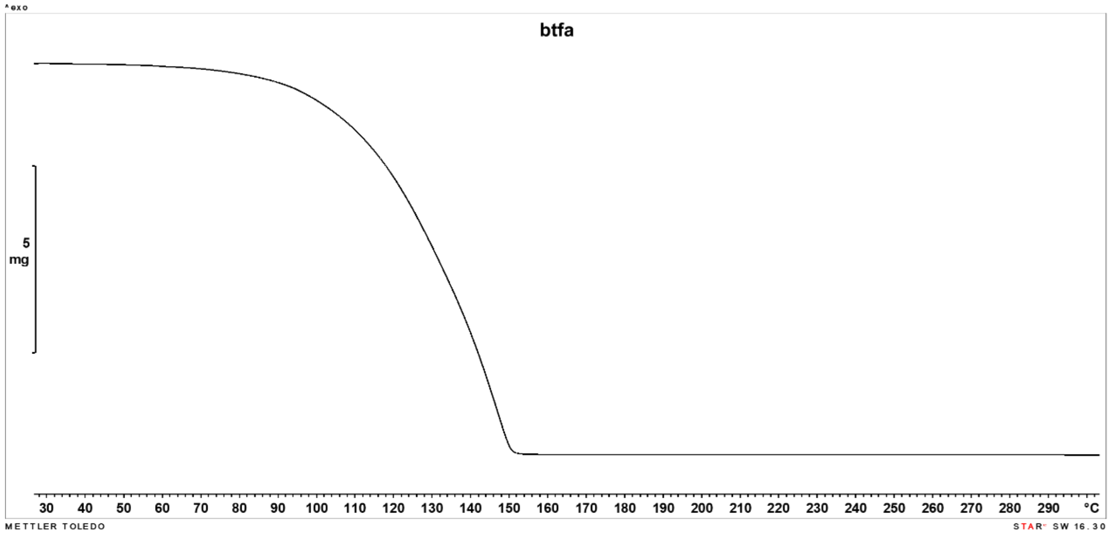

Figure S33 TGA curve of **btfa**.

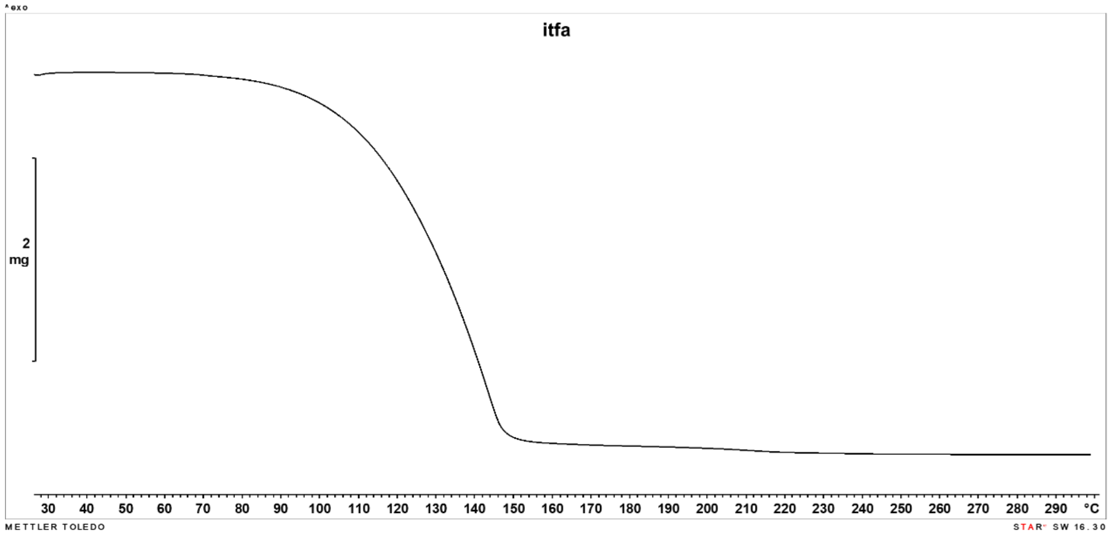

Figure S34 TGA curve of **itfa**.

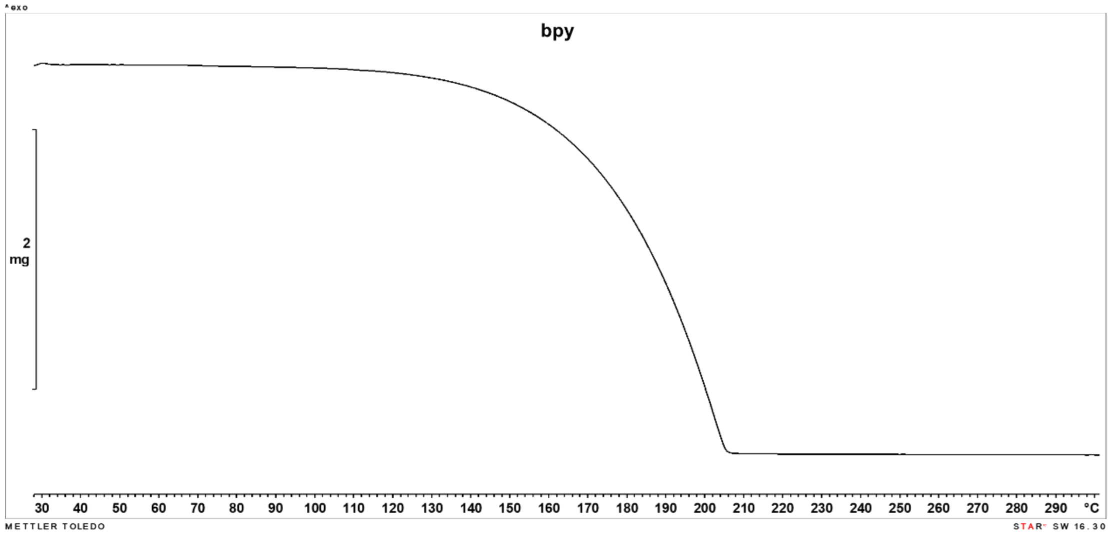

Figure S35 TGA curve of **bpy**.

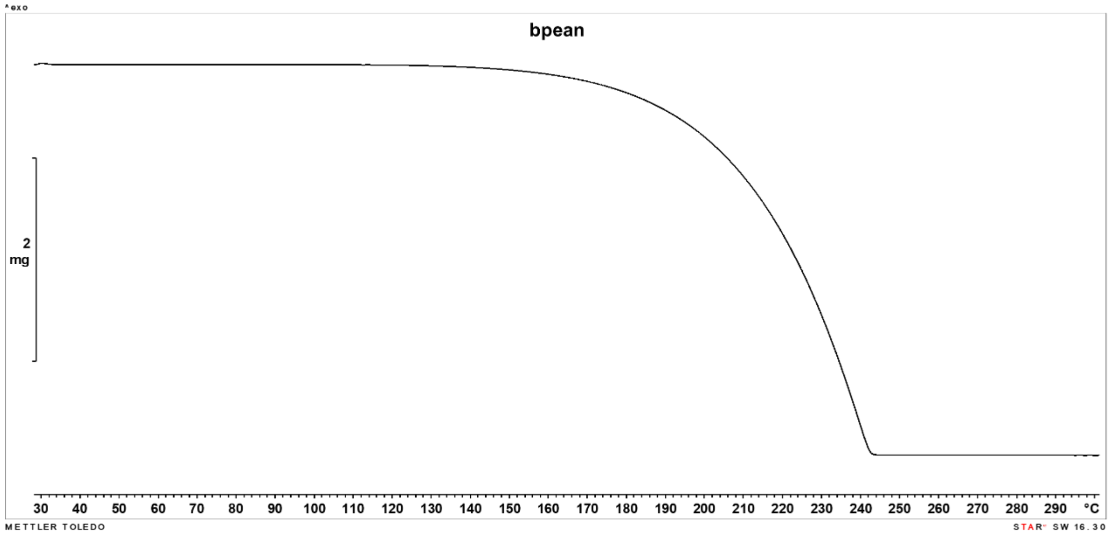

Figure S36 TGA curve of **bpean**.

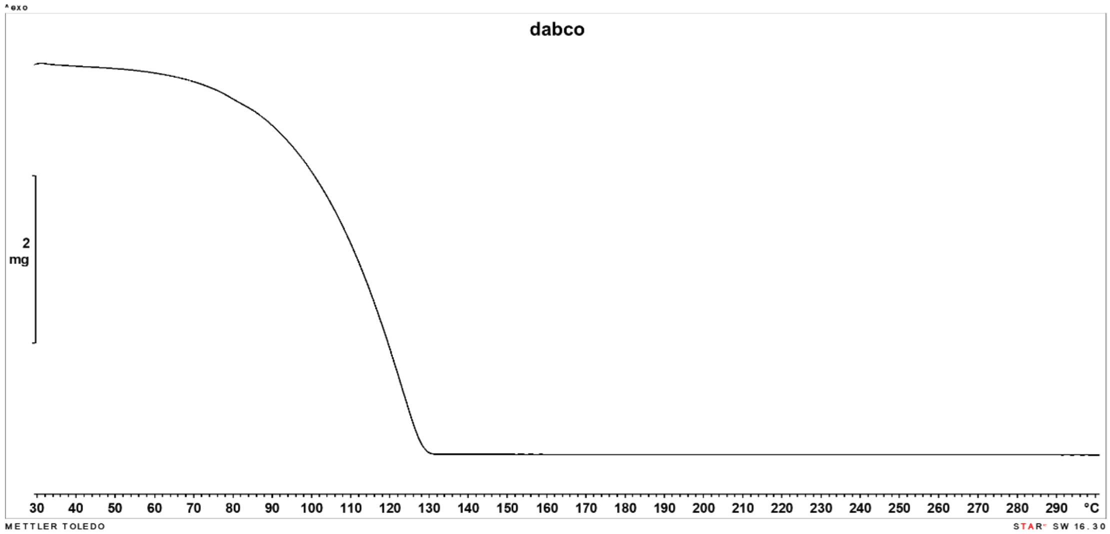

Figure S37 TGA curve of **dabco**.

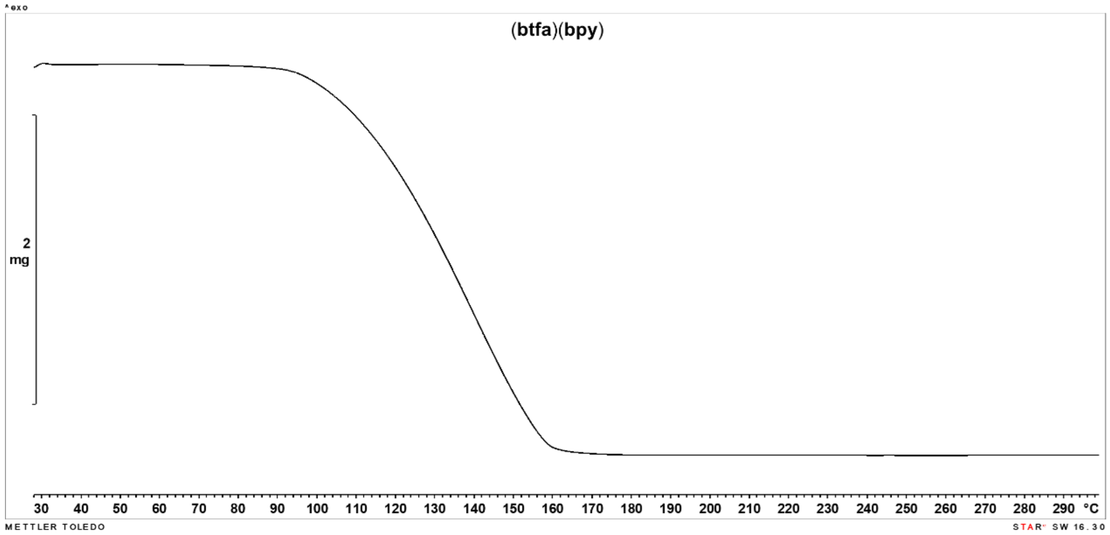

Figure S38 TGA curve of (**btfa**)(**bpy**).

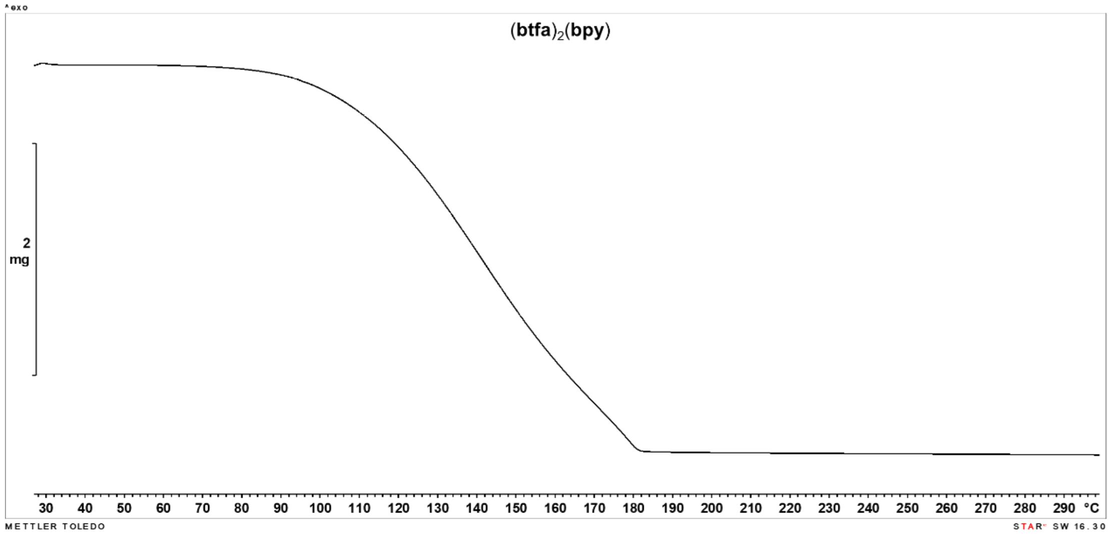

Figure S39 TGA curve of **(btfa)**$_2$**(bpy)**.

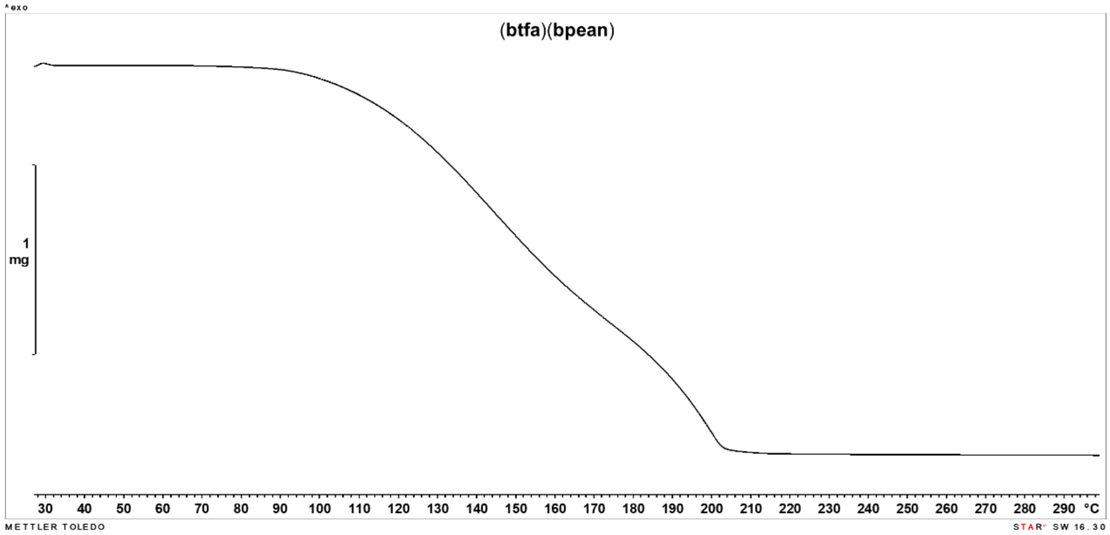

Figure S40 TGA curve of **(btfa)**(**bpean**).

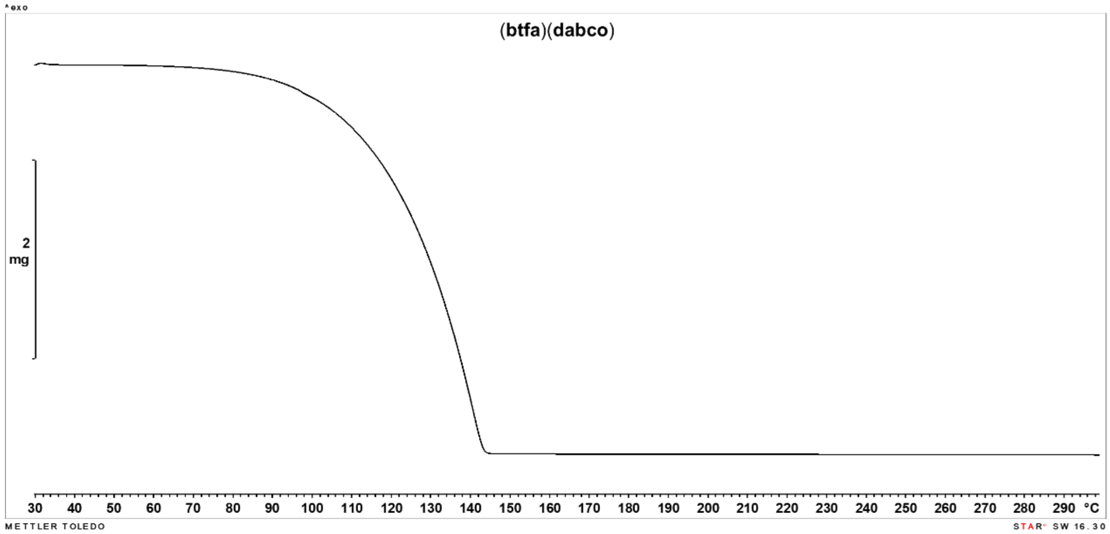

Figure S41 TGA curve of (**btfa**)(**dabco**).

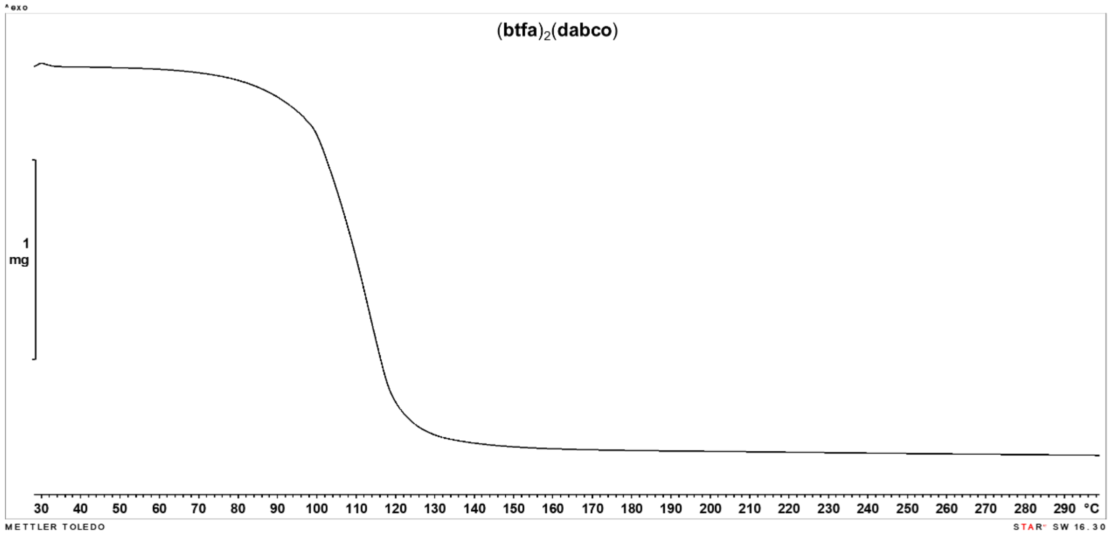

Figure S42 TGA curve of (**btfa**)$_2$(**dabco**).

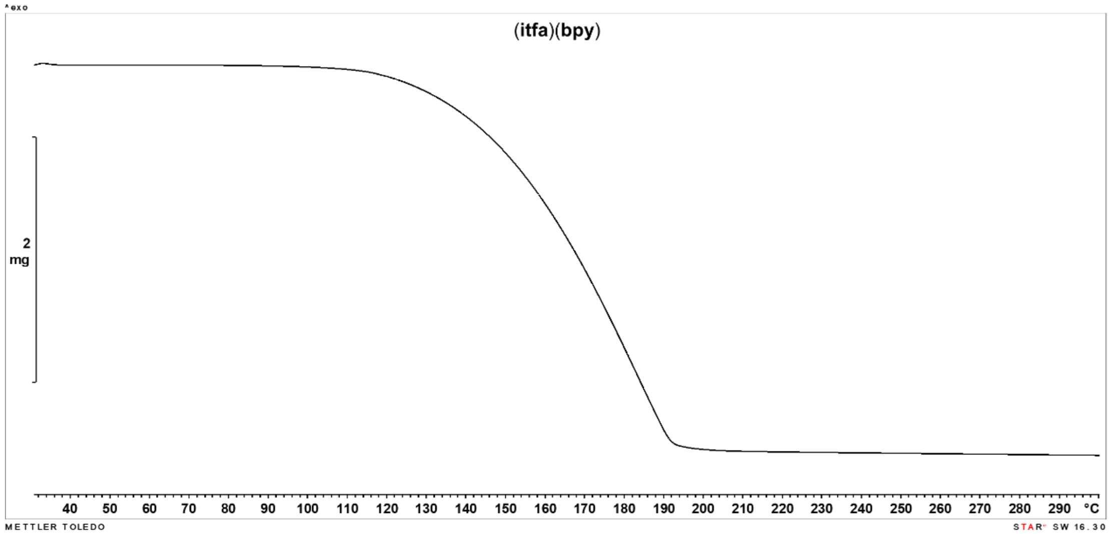

Figure S43 TGA curve of (**itfa**)(**bpy**).

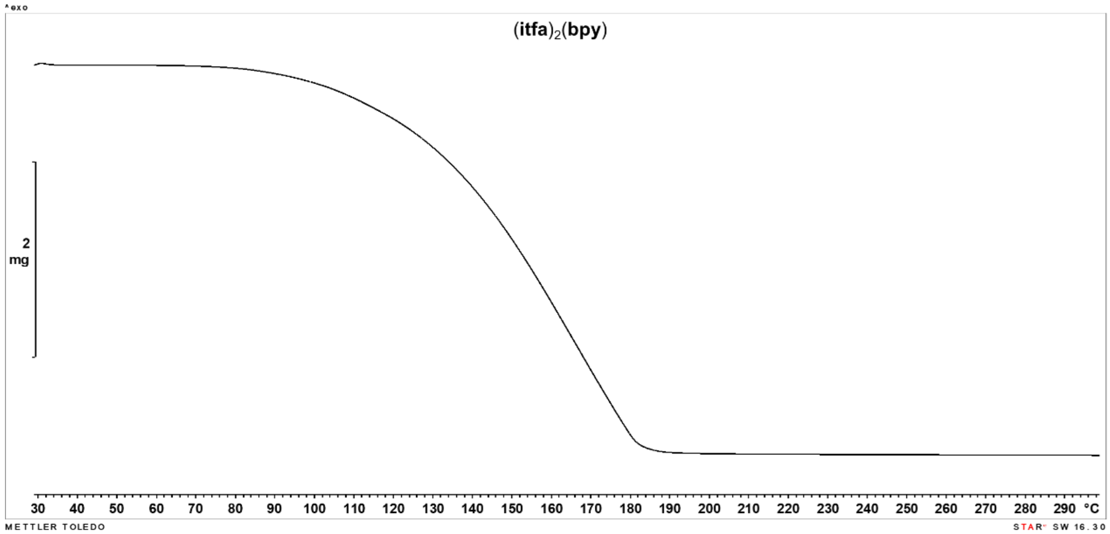

Figure S44 TGA curve of (**itfa**)$_2$(**bpy**).

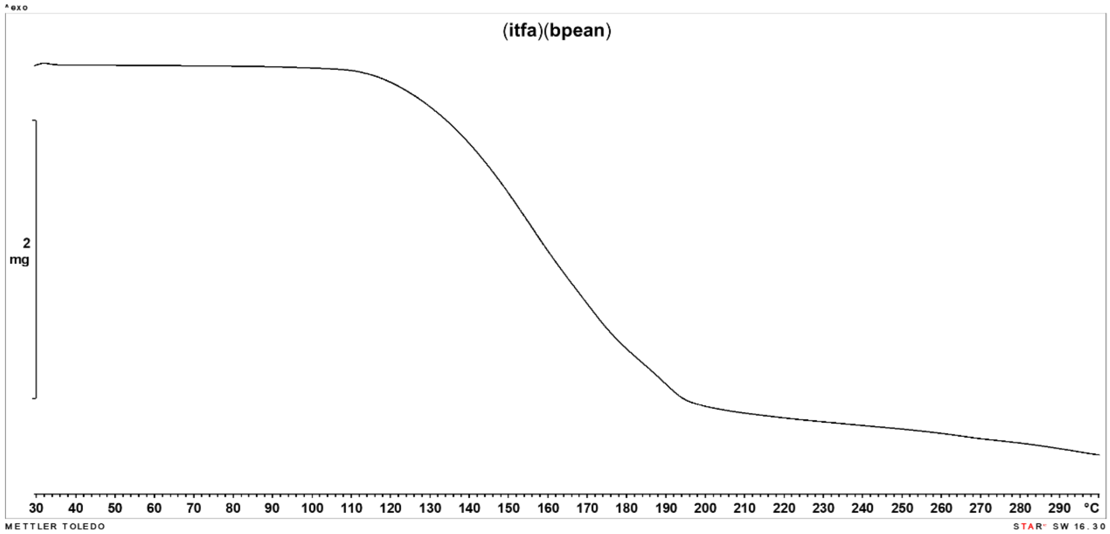

Figure S45 TGA curve of (**itfa**)(**bpean**).

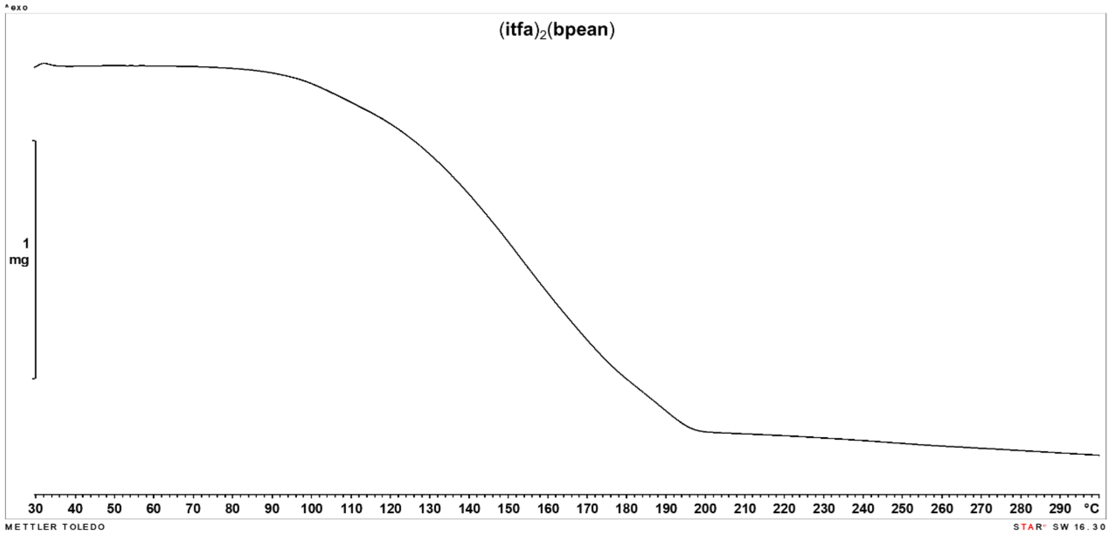

Figure S46 TGA curve of (**itfa**)$_2$(**bpean**).

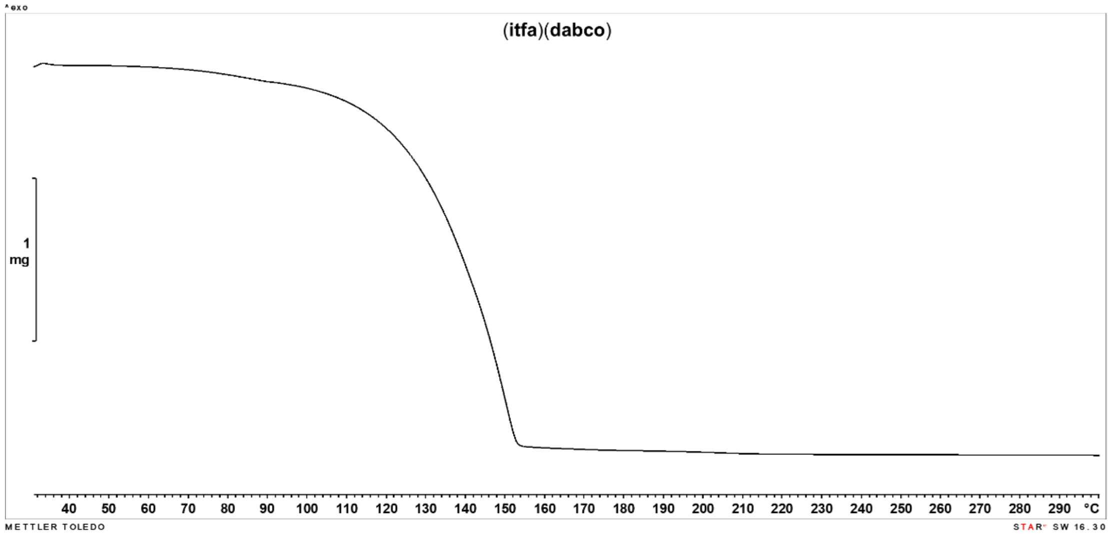

Figure S47 TGA curve of (**itfa**)(**dabco**).

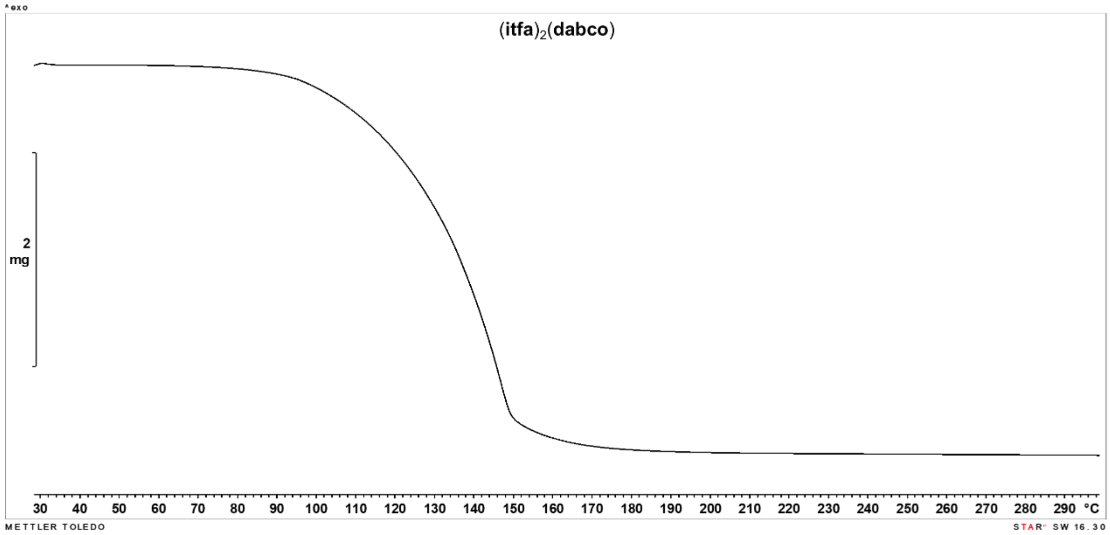

Figure S48 TGA curve of (**itfa**)$_2$(**dabco**).

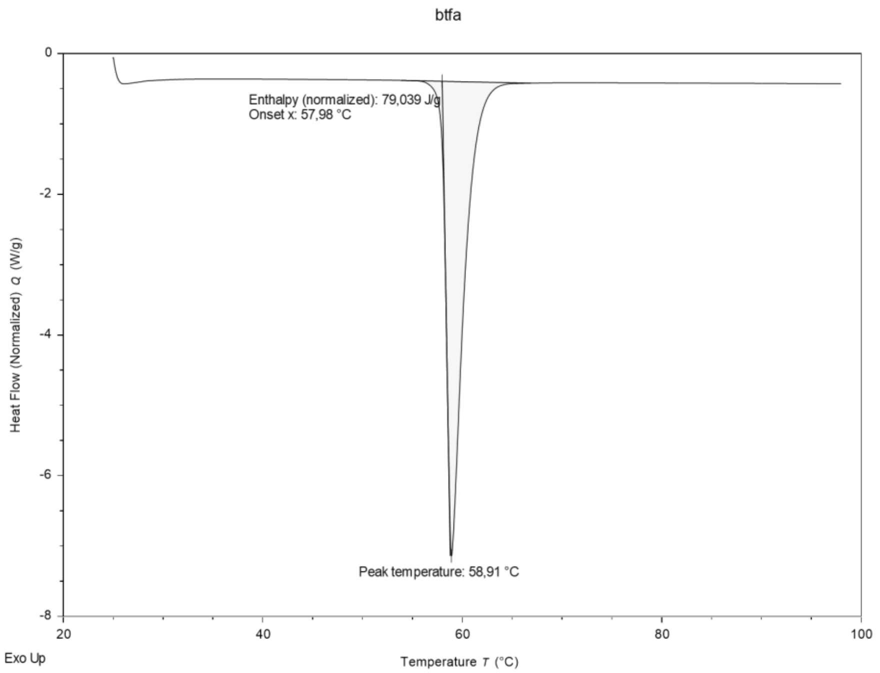

Figure S49 DSC thermogram of **btfa**.

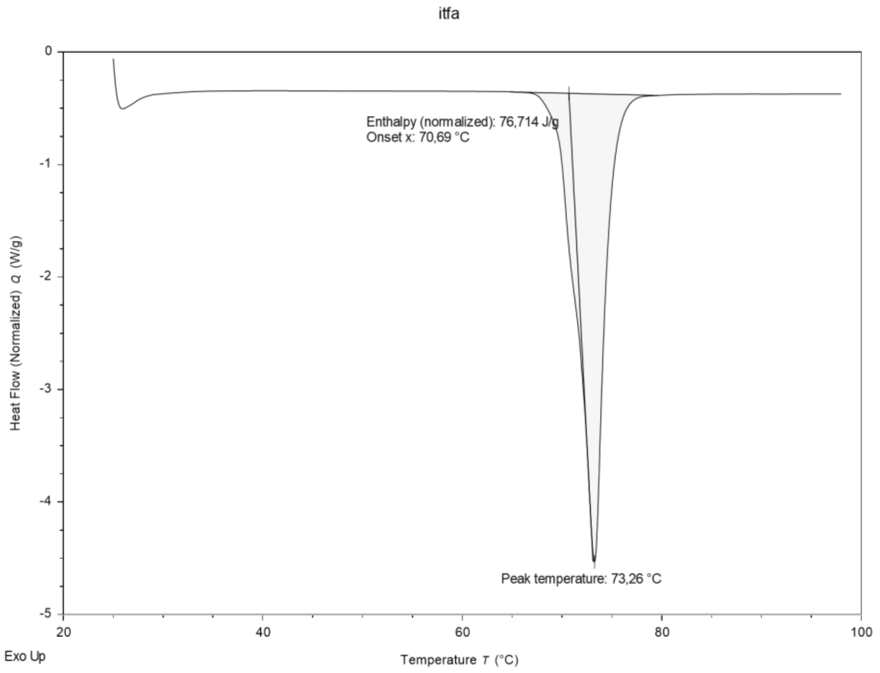

Figure S50 DSC thermogram of **itfa**.

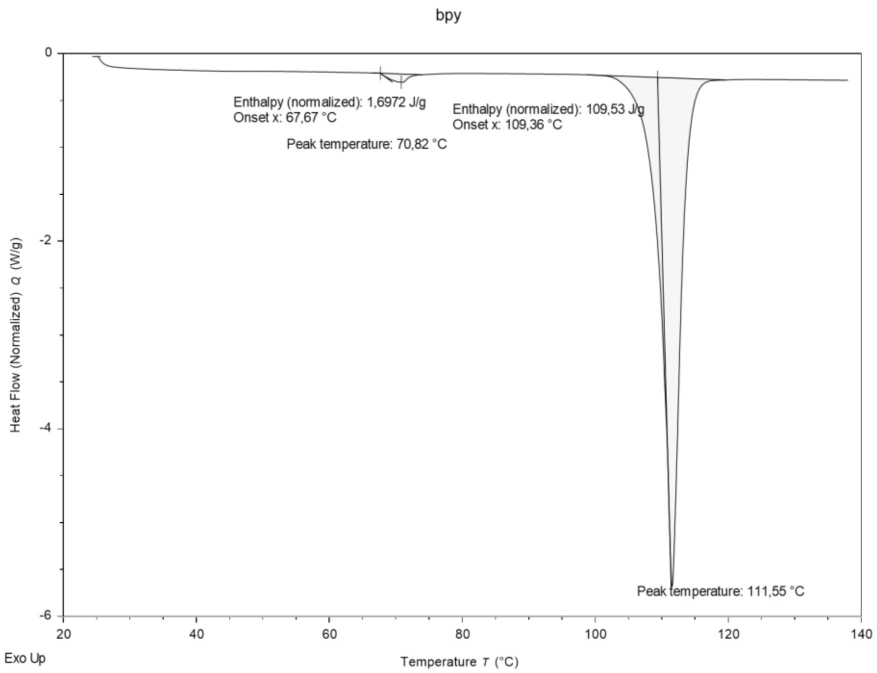

Figure S51 DSC thermogram of **bpy**.

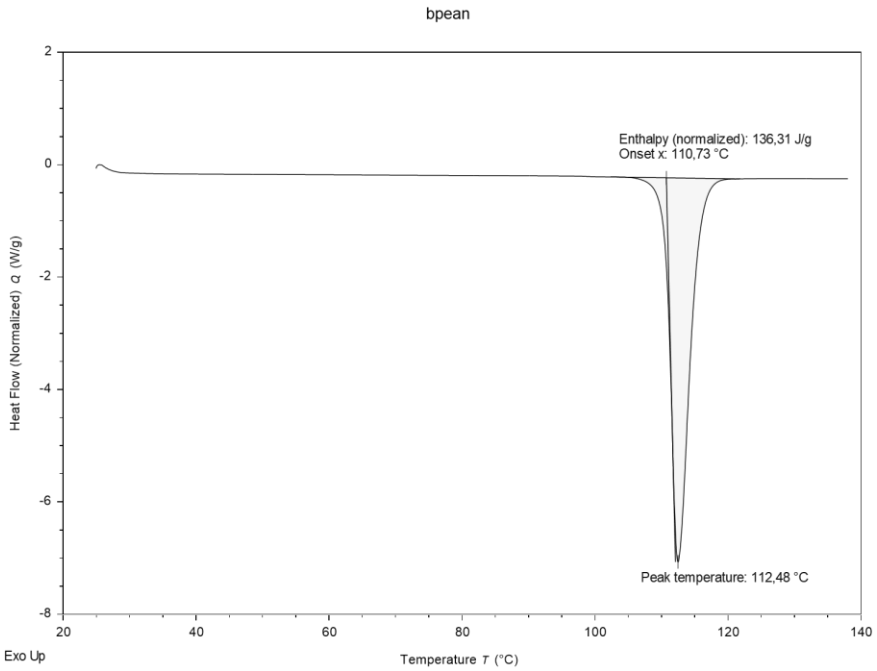

Figure S52 DSC thermogram of **bpean**.

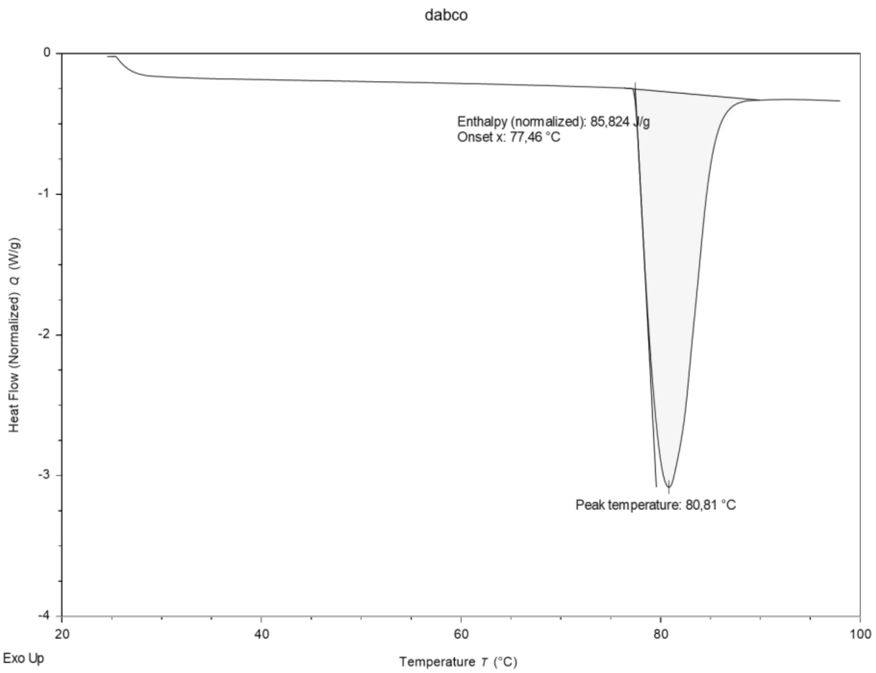

Figure S53 DSC thermogram of **dabco**.

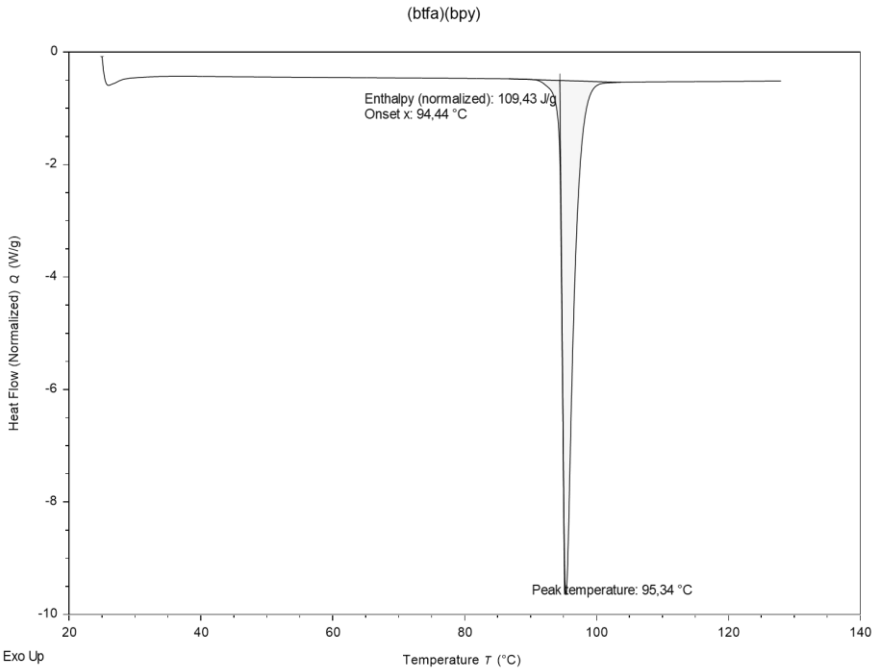

Figure S54 DSC thermogram of (**btfa**)(**bpy**).

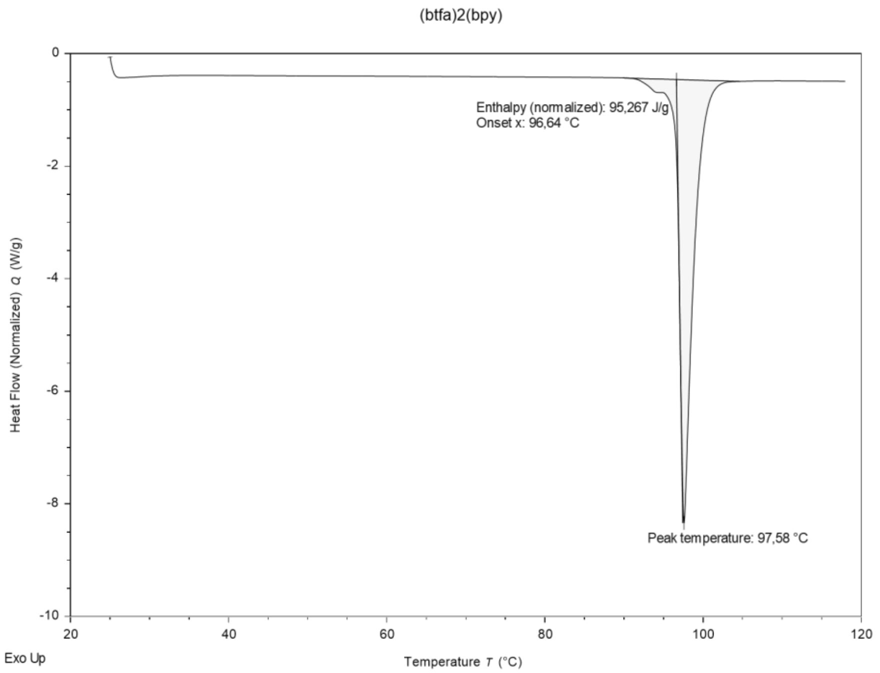

Figure S55 DSC thermogram of (**btfa**)$_2$(**bpy**).

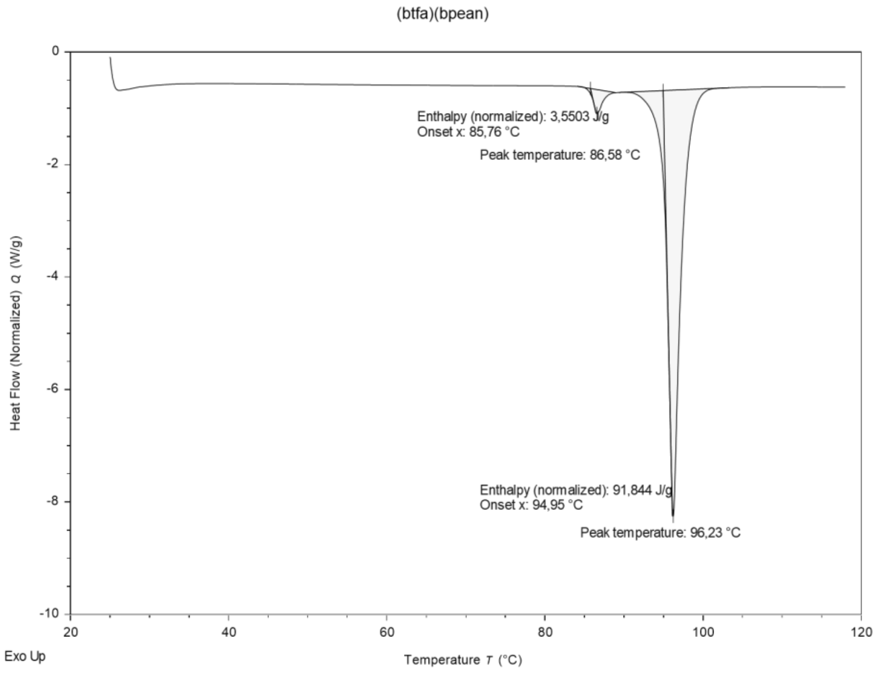

Figure S56 DSC thermogram of (**btfa**)(**bpean**).

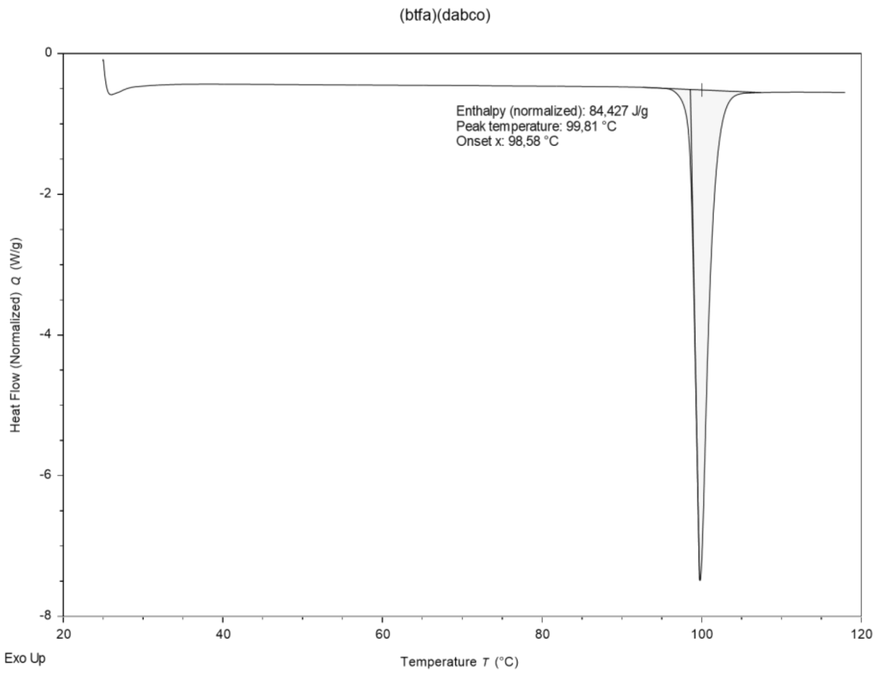

Figure S57 DSC thermogram of (**btfa**)(**dabco**).

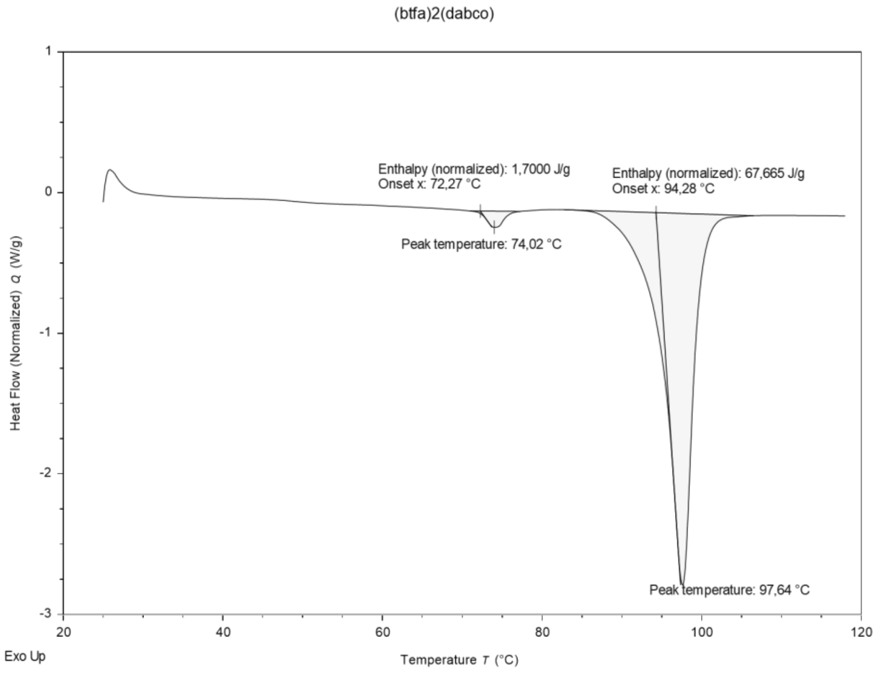

Figure S58 DSC thermogram of (**btfa**)$_2$(**dabco**).

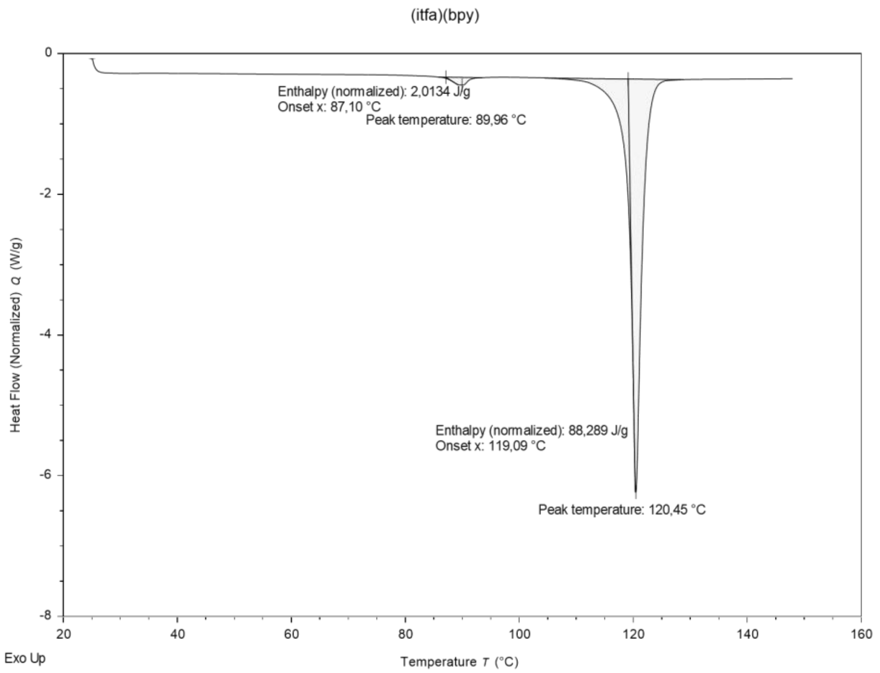

Figure S59 DSC thermogram of (**itfa**)(**bpy**).

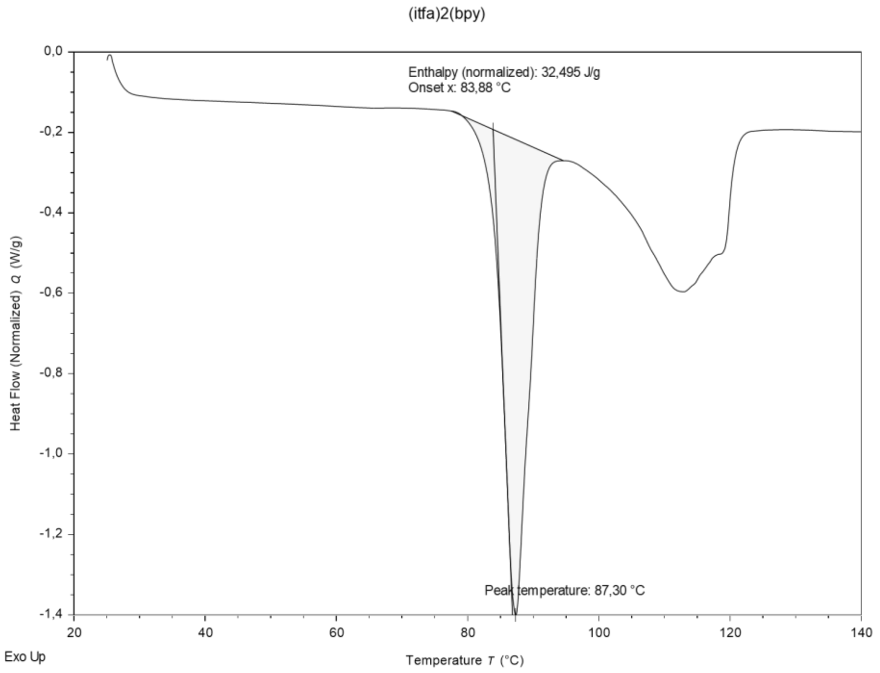

Figure S60 DSC thermogram of (**itfa**)$_2$(**bpy**).

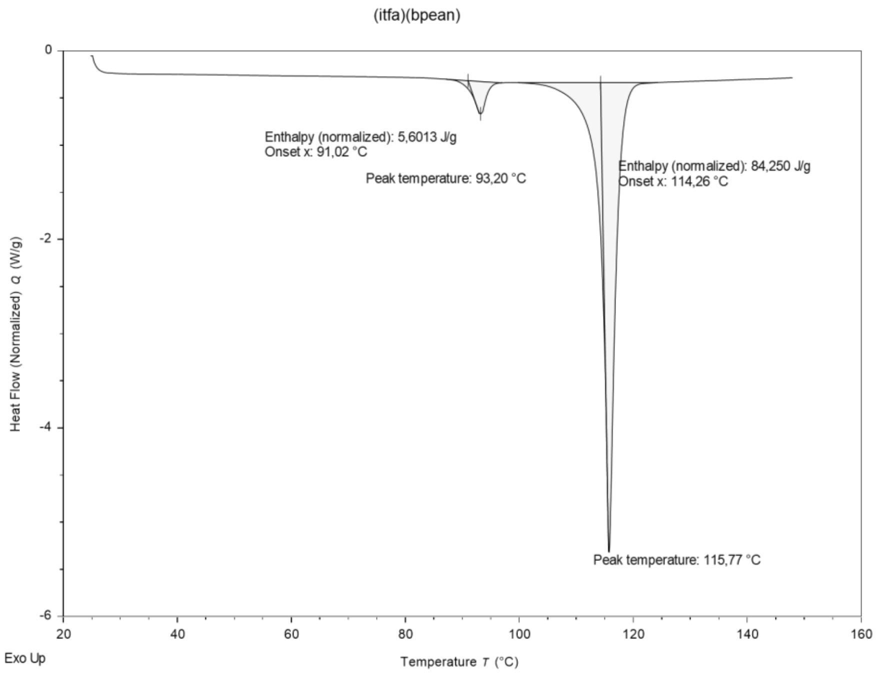

Figure S61 DSC thermogram of (**itfa**)(**bpean**).

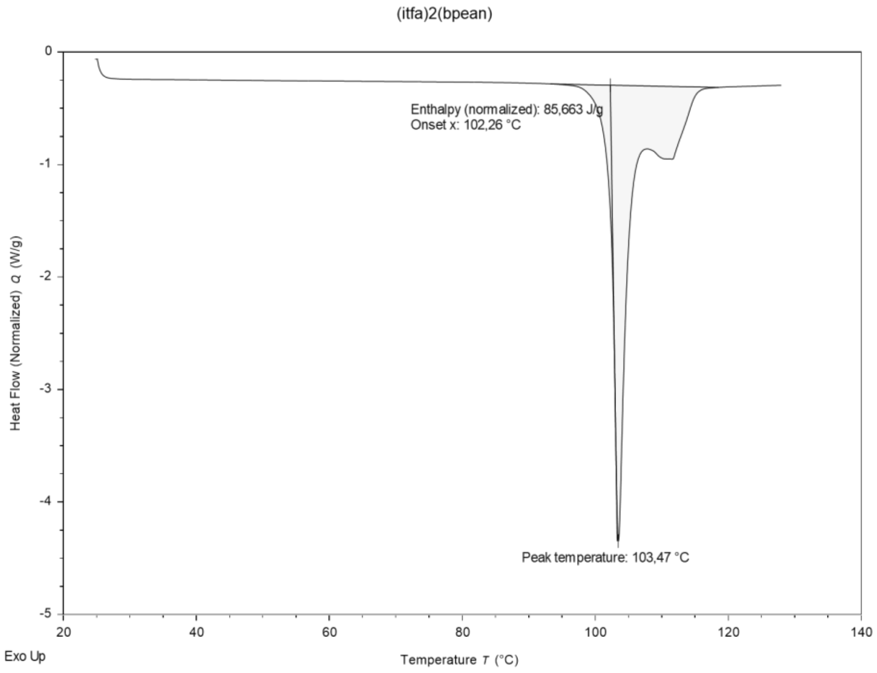

Figure S62 DSC thermogram of (**itfa**)$_2$(**bpean**).

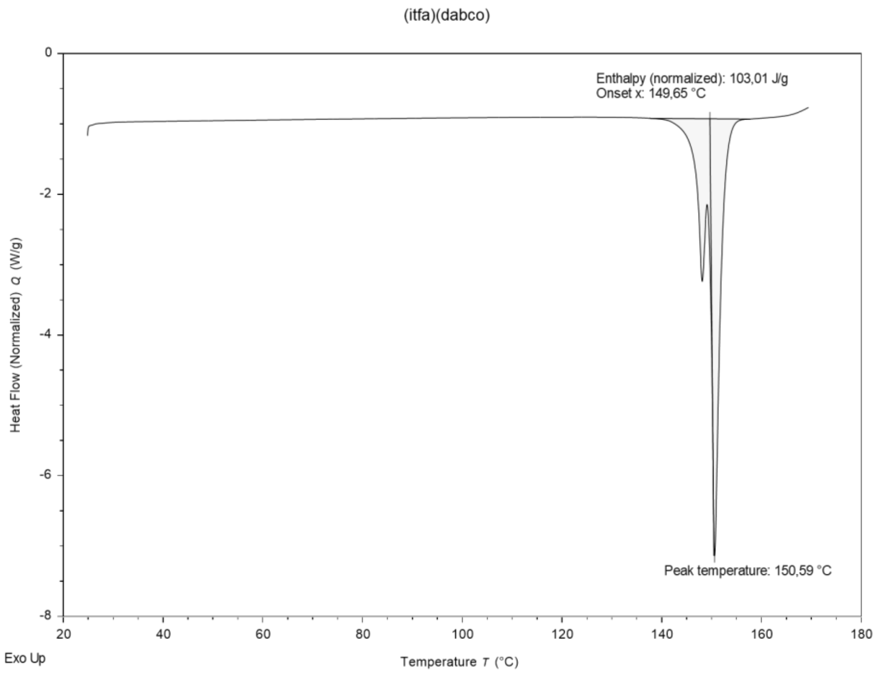

Figure S63 DSC thermogram of (**itfa**)(**dabco**).

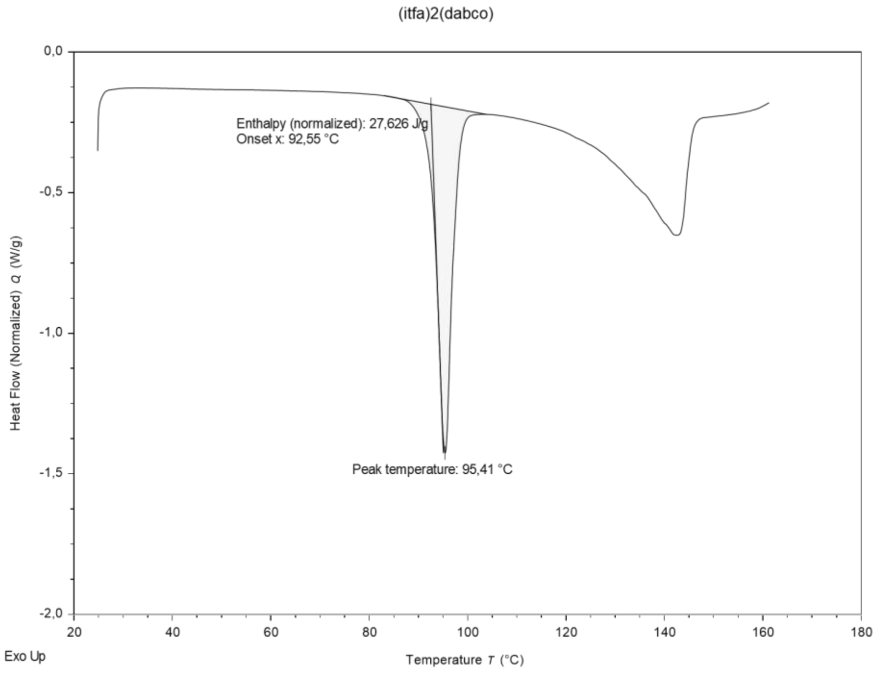

Figure S64 DSC thermogram of (**itfa**)$_2$(**dabco**).

Table S6 The calculated electronic energies (per formula unit) of all the cocrystals and their starting components

Compound	Refcode	Energy per formula unit (eV)	
		PBE + MBD*	PBE + D3
**btfa**	This work	-4364.30189	-4364.21631
**itfa**	This work	-4696.63073	-4696.54305
**bpy**	HIQWEJ01	-2258.60846	-2258.40990
**bpean**	ZEXKIW	-2639.04576	-2638.79256
**dabco**	TETDAM01	-1692.85312	-1692.60142
(**btfa**)(**bpy**)	This work	-6623.14788	-6622.89140
(**btfa**)$_2$(**bpy**)	This work	-10987.49285	-10987.18876
(**btfa**)(**bpean**)	This work	-7003.60820	-7003.27410
(**btfa**)(**dabco**)	This work	-6057.20350	-6056.95891
(**btfa**)$_2$(**dabco**)	This work	-10421.74625	-10421.38030
(**itfa**)(**bpy**)	This work	-6955.45248	-6955.16075
(**itfa**)$_2$(**bpy**)	This work	-11652.18385	-11651.79326
(**itfa**)(**bpean**)	This work	-7335.88805	-7335.54228
(**itfa**)$_2$(**bpean**)	This work	-12032.69045	-12032.22010
(**itfa**)(**dabco**)	This work	-6389.79393	-6389.49833
(**itfa**)$_2$(**dabco**)	This work	-11086.35650	-11085.98510

Table S7 The calculated formation energies of all the cocrystals. In addition to the formation energy per formula unit, the normalized formation energies** of the 1:1 and 2:1 cocrystals with similar constituents were calculated.

	Formation energy per cocrystal formula unit (kJ/mol)		Normalized formation energy (kJ/mol)	
**Cocrystal**	**PBE + MBD***	**PBE + D3**	**PBE + MBD***	**PBE + D3**
(**btfa**)(**bpy**)	-22.92	-25.59	-11.46	-12.80
(**btfa**)$_2$(**bpy**)	-27.08	-33.40	-9.03	-11.13
(**btfa**)(**bpean**)	-25.14	-25.59	-12.57	-12.80
(**btfa**)(**dabco**)	-4.68	-13.62	-2.34	-6.81
(**btfa**)$_2$(**dabco**)	-27.92	-33.41	-9.31	-11.14
(**itfa**)(**bpy**)	-20.58	-20.05	-10.29	-10.03
(**itfa**)$_2$(**bpy**)	-30.30	-28.68	-10.10	-9.56
(**itfa**)(**bpean**)	-20.41	-19.94	-10.21	-9.97
(**itfa**)$_2$(**bpean**)	-36.98	-32.94	-12.33	-10.98
(**itfa**)(**dabco**)	-29.92	-34.14	-14.96	-17.07
(**itfa**)$_2$(**dabco**)	-23.34	-28.71	-7.78	-9.57

** Normalized formation energies were calculated by dividing the total formation energy with total number of molecules (2 for 1:1 and 3 for 2:1 cocrystals). These normalized formation energies were used to compare the stability of the cocrystals with similar constituents and different stoichiometry.

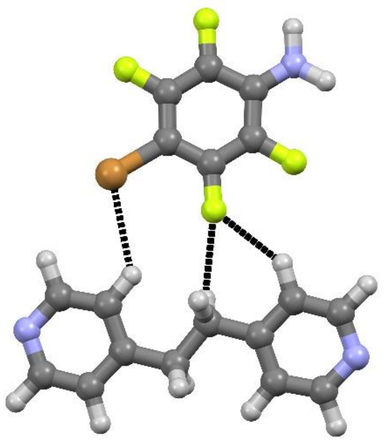

C9-H9···Br1/C13-H13A···F2/C15-H15···F2
(-14.88 kJ/mol)
(a)

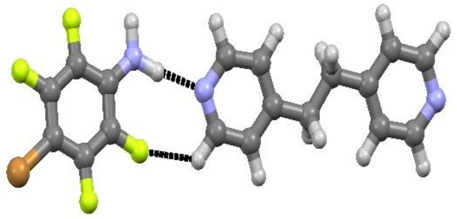

N1-H1A···N2/C11-H11···F1 (-39.04 kJ/mol)
(b)

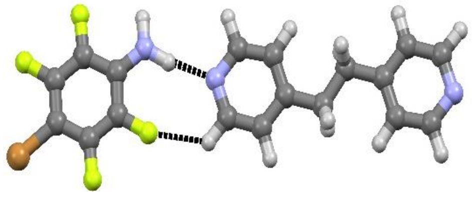

N1-H1B···N3/C16-H16···F4 (-40.16 kJ/mol)
(c)

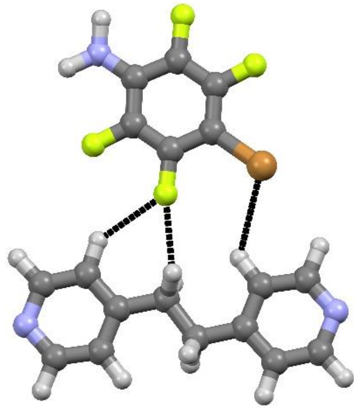

C18-H18···Br1/C7-H7A···F3/C12-H12···F3
(-15.34 kJ/mol)
(d)

Figure S65 Heteromolecular non-covalent bonded dimers used for the calculation of interaction energy (given in parentheses) from optimized geometry of (**btfa**)(**bpean**) cocrystal.

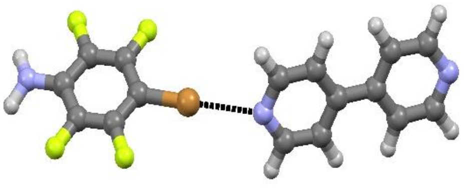

C1-Br1···N3 (-18.70 kJ/mol)
(a)

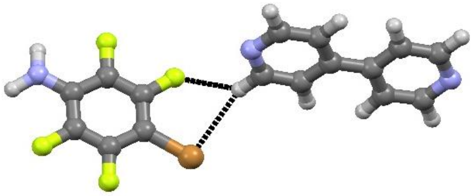

C14-H14···Br1/C14-H14···F2 (-6.64 kJ/mol)
(b)

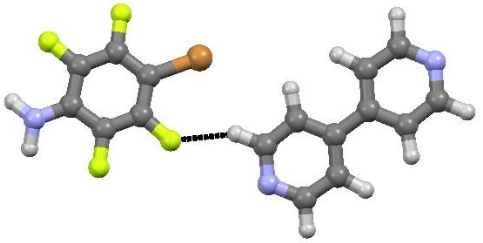

C9-H9···F3 (-6.63 kJ/mol)
(c)

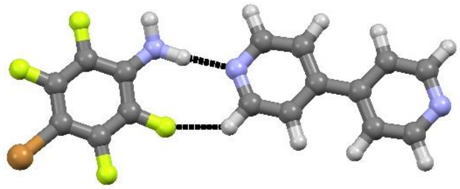

N1-H1B···N2/C10-H10···F4 (-37.56 kJ/mol)
(d)

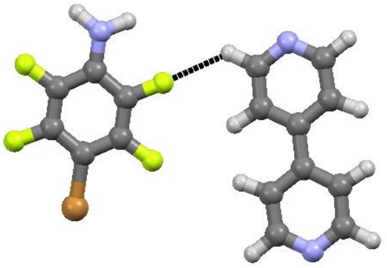

C10-H10···F1 (-10.84 kJ/mol)
(e)

Figure S66 Heteromolecular non-covalent bonded dimers used for the calculation of interaction energy (given in parentheses) from optimized geometry of (**btfa**)(**bpy**) cocrystal.

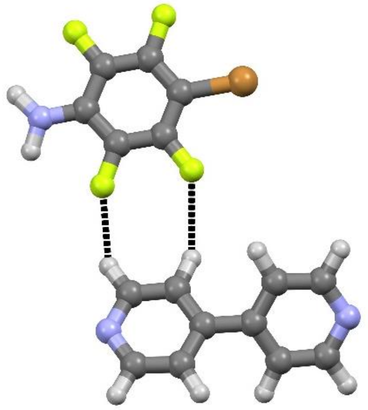

C16A-H16A···F5/C17A-H17A···F6 (-10.48 kJ/mol)
(a)

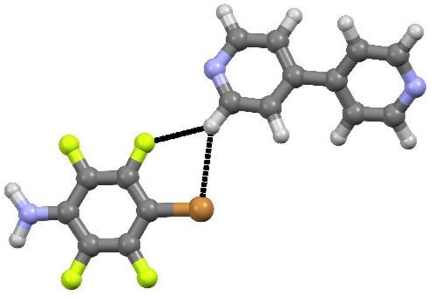

C15A-H15A···Br1/C15A-H15A···F7 (-5.95 kJ/mol)
(b)

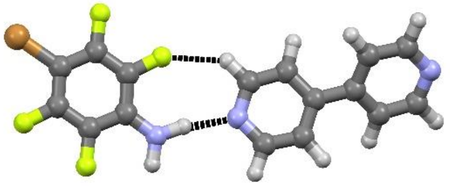

N1A-H1AB···N3A/C16A-H16A···F4A (-39.77 kJ/mol)
(c)

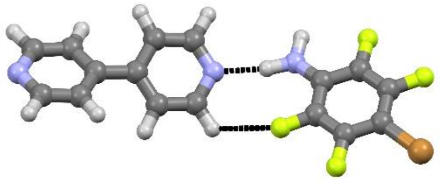

N2-H2B··· N4/C21-H21···F8 (-39.15 kJ/mol)
(d)

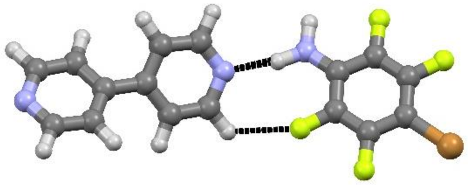

N2A-H2AA···N4A/C20A-H20A···F5A (-39.65 kJ/mol)
(e)

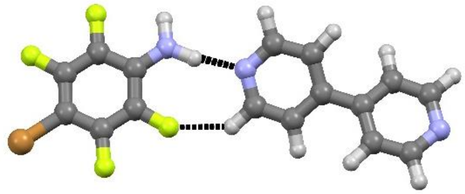

N1-H1A··· N3/C15-H15···F1 (-39.98 kJ/mol)
(f)

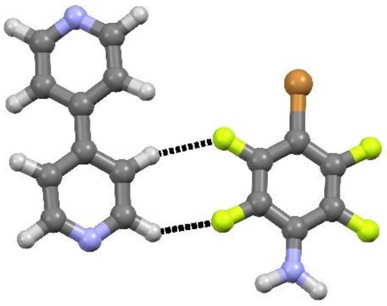

C22-H22···F2A/C21-H21···F1A (-10.71 kJ/mol)
(g)

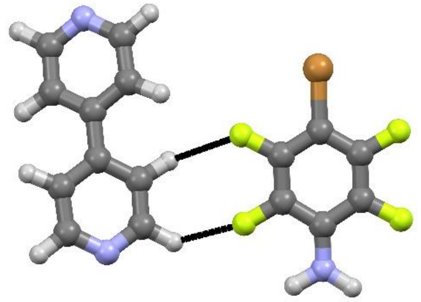

C15-H15···F8A/C14-H14···F7A (-10.92 kJ/mol)
(h)

Figure S67 Heteromolecular non-covalent bonded dimers used for the calculation of interaction energy (given in parentheses) from optimized geometry of (**btfa**)$_2$(**bpy**) cocrystal.

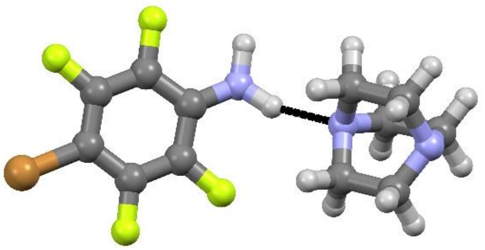

N1-H1···N2 (-41.01 kJ/mol)
(a)

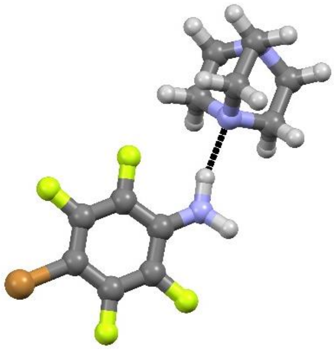

N1-H2···N16 (-40.57 kJ/mol)
(b)

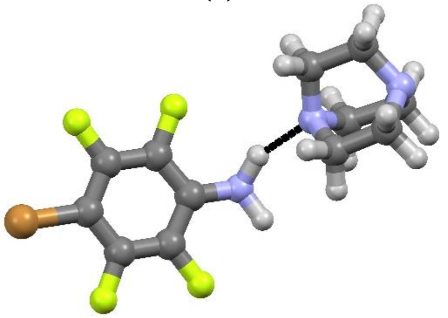

N4-H15···N6 (-38.20 kJ/mol)
(c)

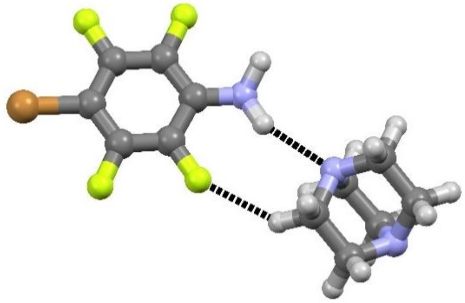

N4-H16···N15/C60-H73···F7 (-30.58 kJ/mol)
(d)

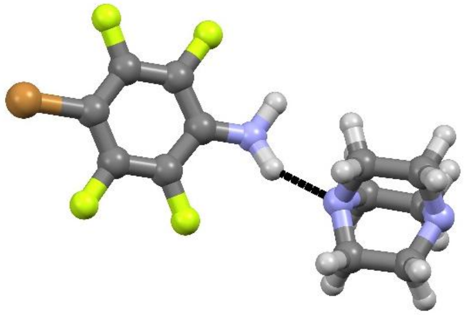

N9-H41···N10 (-33.88 kJ/mol)
(e)

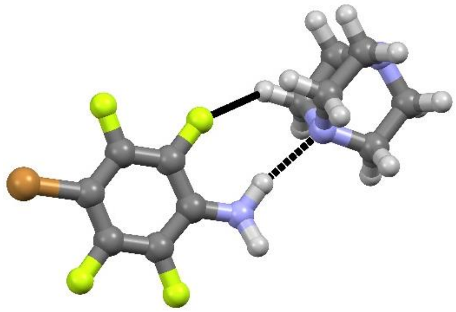

N9-H42···N7/C25-H30···F11 (-36.10 kJ/mol)
(f)

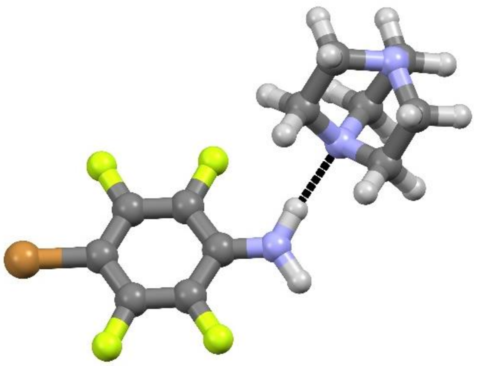

N12-H55···N13 (-42.04 kJ/mol)
(g)

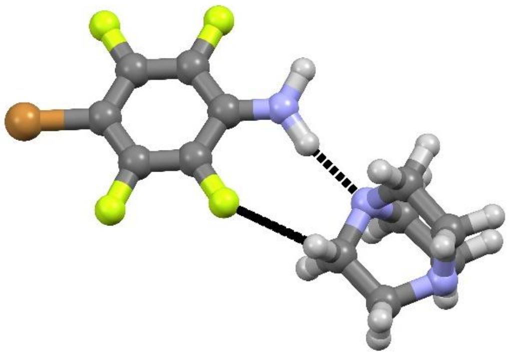

N12-H56···N8/C30-H36···F15 (-41.17 kJ/mol)
(h)

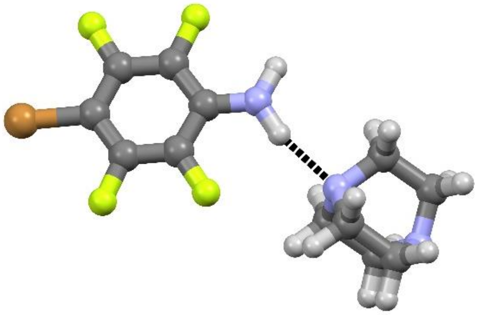

N17-H81···N3 (-40.42 kJ/mol)
(i)

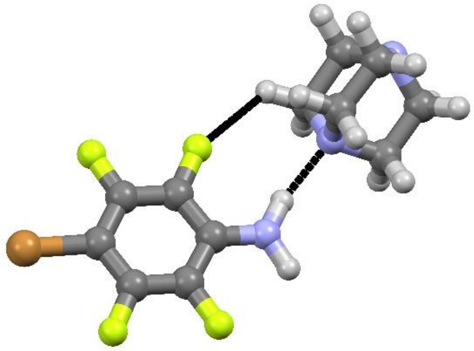

N17-H82···N23/C96-H108···F19 (-41.15 kJ/mol)
(j)

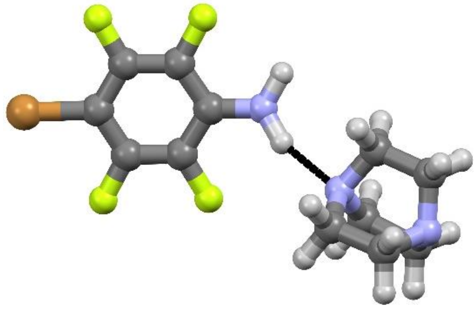

N18-H83···N5 (-37.92 kJ/mol)
(k)

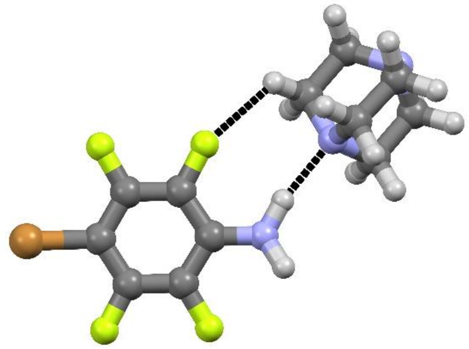

N18-H84···N24/C91-H112···F23 (-39.32 kJ/mol)
(l)

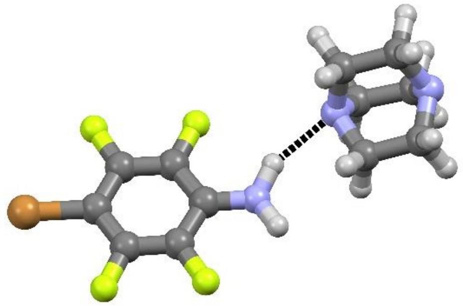

N21-H97···N11 (-30.53 kJ/mol)
(m)

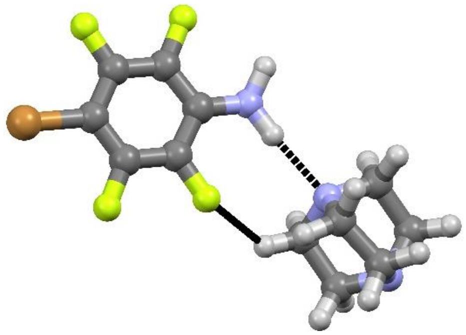

N21-H98···N20/C73-H92···F27 (-38.80 kJ/mol)
(n)

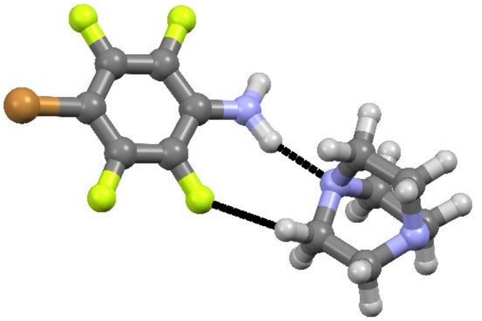

N22-H99···N14/C54-H66···F30 (-39.96 kJ/mol)
(o)

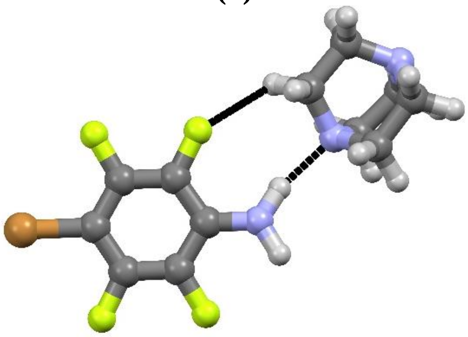

N22-H100···N19/C78-H94···F31 (-41.58 kJ/mol)
(p)

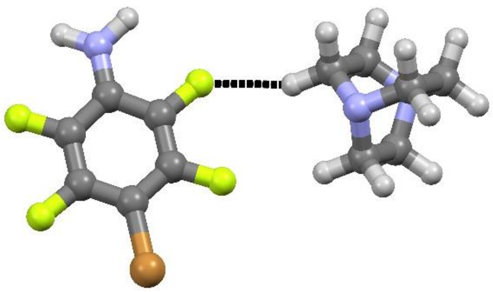

C7-H8···F30 (-6.89 kJ/mol)
(q)

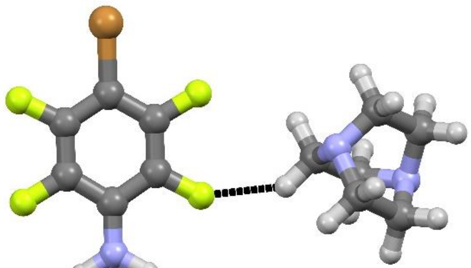

C12-H14···F14 (-4.16 kJ/mol)
(r)

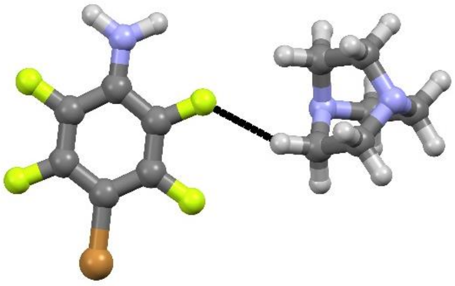

C21-H22···F26 (-6.13 kJ/mol)
(s)

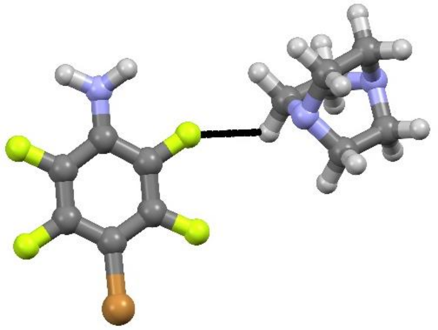

C24-H26···F10 (-3.22 kJ/mol)
(t)

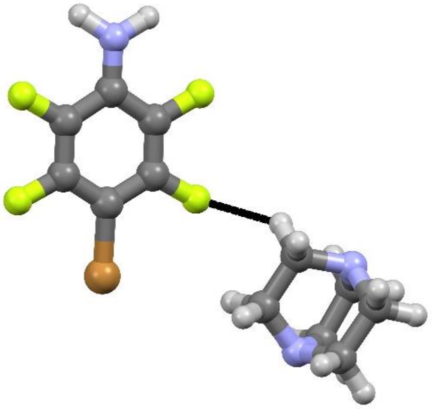

C29-H33···F4 (-8.79 kJ/mol)
(u)

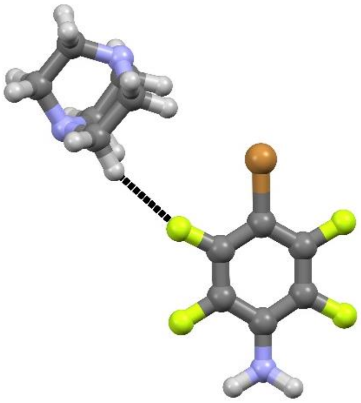

C28-H37···F8 (-9.14 kJ/mol)
(v)

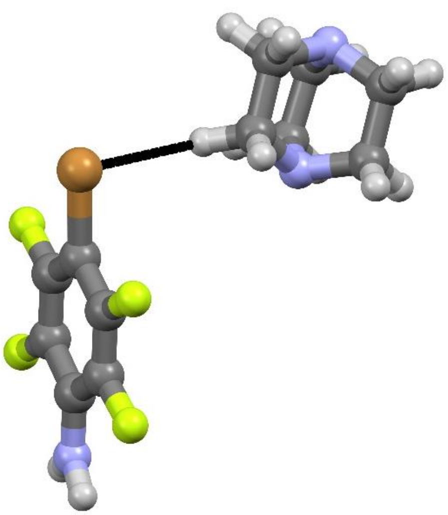

C41-H44···Br4 (-5.92 kJ/mol)
(w)

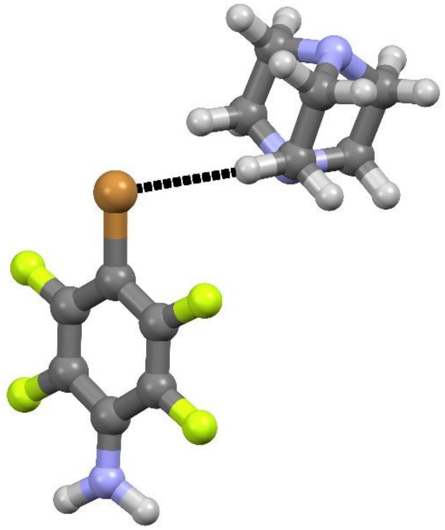

C40-H45···Br8 (-7.88 kJ/mol)
(x)

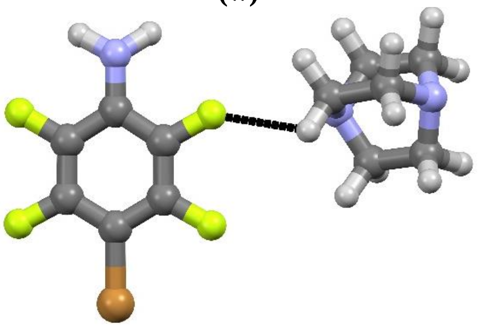

C42-H47···F6 (-4.51 kJ/mol)
(y)

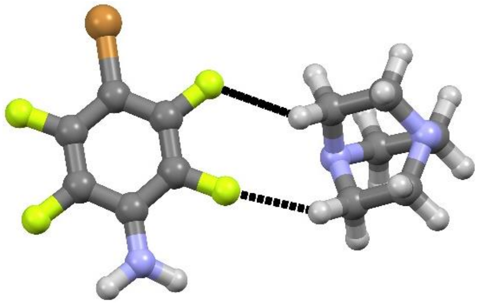

C37-H50···F22/C39-H53···F21 (-4.62 kJ/mol)
(z)

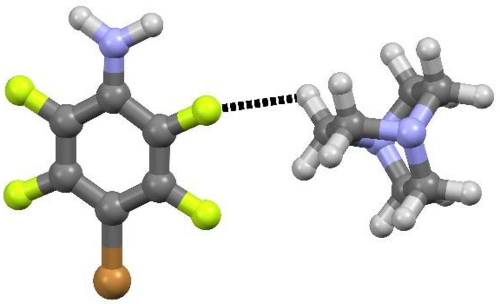

C51-H57···F18 (-4.70 kJ/mol)
(aa)

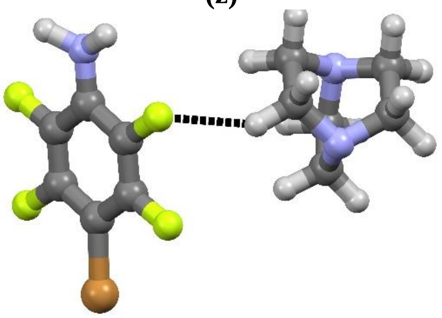

C49-H61···F7 (-8.21 kJ/mol)
(ab)

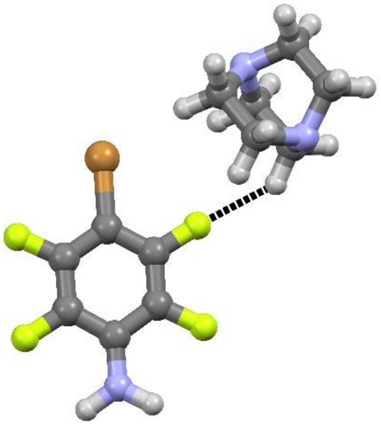

C58-H72···F16 (-10.91 kJ/mol)
(ac)

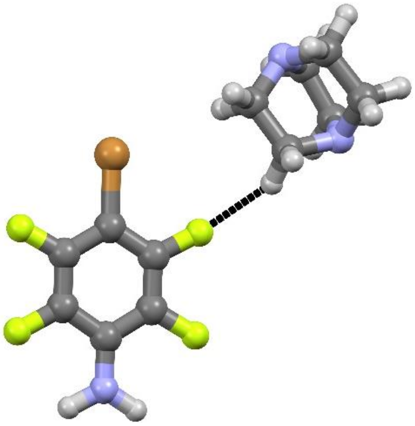

C77-H96···F20 (-7.52 kJ/mol)
(ad)

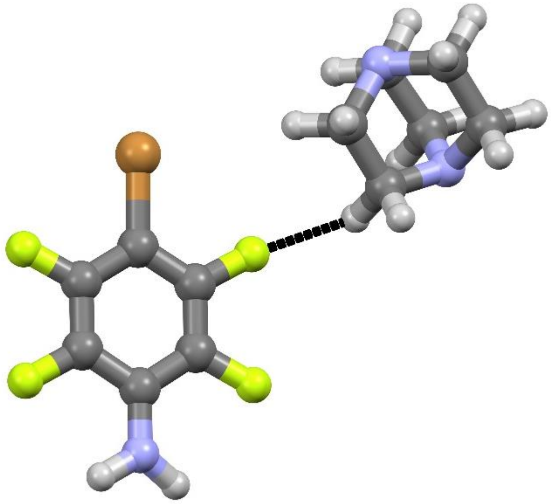

C95-H105···F32 (-6.92 kJ/mol)
(ae)

Figure S68 Heteromolecular non-covalent bonded dimers used for the calculation of interaction energy (given in parentheses) from optimized geometry of (**btfa**)(**dabco**) cocrystal (The labels are not consistent with the experimental).

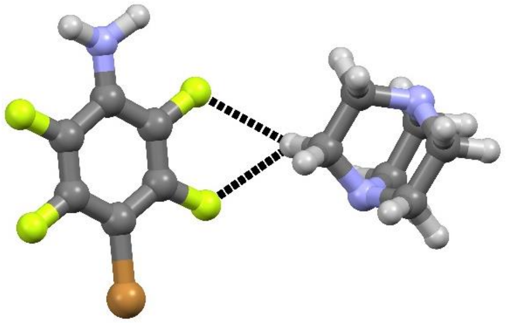

C18-H18A···F6/C18-H18A···F5 (-5.98 kJ/mol)
(a)

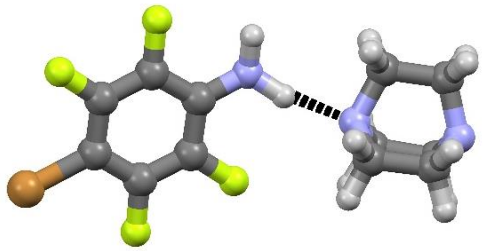

N2-H2A···N4 (-44.58 kJ/mol)
(b)

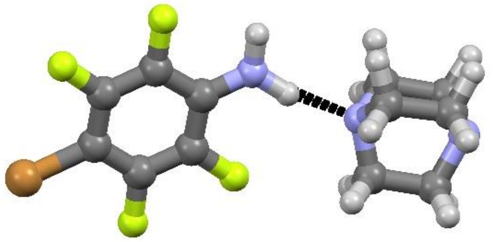

N1-H1B···N3 (-44.58 kJ/mol)
(c)

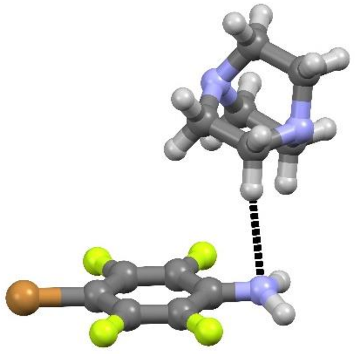

C16-H16B···N2 (-13.41 kJ/mol)
(d)

Figure S69 Heteromolecular non-covalent bonded dimers used for the calculation of interaction energy (given in parentheses) from optimized geometry of (**btfa**)$_2$(**dabco**) cocrystal.

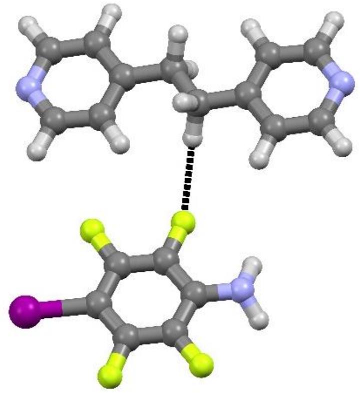

C7-H17A···F1 (-13.33 kJ/mol)
(a)

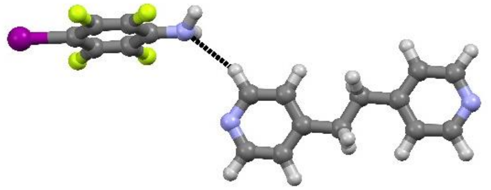

C11- H11··· N1 (-3.64 kJ/mol)
(b)

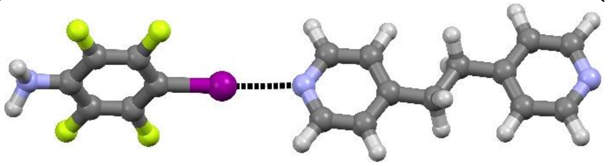

C4-I1··· N2 (-34.64 kJ/mol)
(c)

Figure S70 Heteromolecular non-covalent bonded dimers used for the calculation of interaction energy (given in parentheses) from optimized geometry of (**itfa**)$_2$(**bpean**) cocrystal.

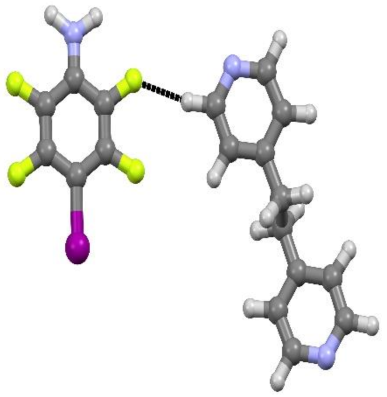

C16-H16···F4 (-8.84 kJ/mol)
(a)

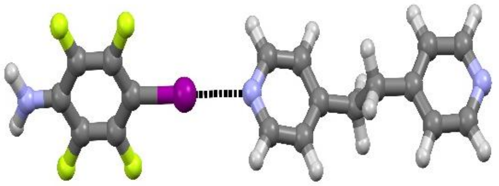

C4-I1···N2 (-32.23 kJ/mol)
(b)

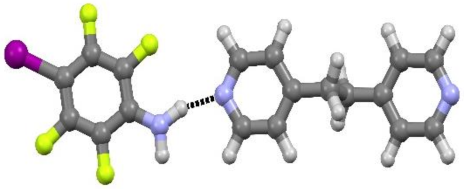

N1-H1B···N3 (-37.99 kJ/mol)
(c)

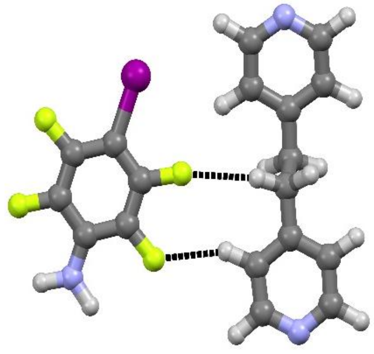

C18-H18···F1/C13-H13A···F2 (-17.57 kJ/mol)
(d)

Figure S71 Heteromolecular non-covalent bonded dimers used for the calculation of interaction energy (given in parentheses) from optimized geometry of (**itfa**)(**bpean**) cocrystal.

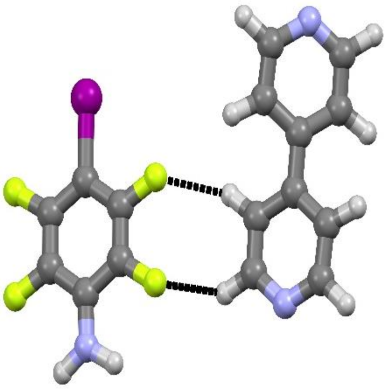

C10-H10···F1/C11-H11···F2 (-10.14 kJ/mol)
(a)

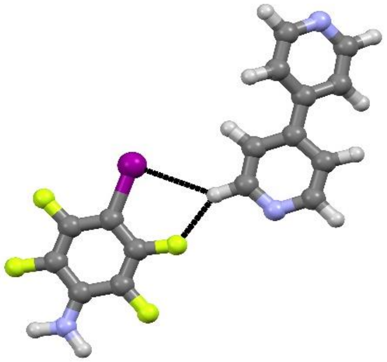

C9-H9···F3/C9-H9···I1 (-6.29 kJ/mol)
(b)

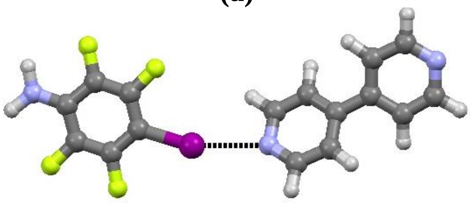

C4-I1···N3 (-24.73 kJ/mol)
(c)

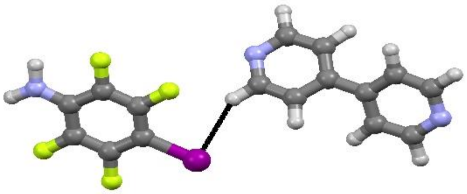

C14-H14···I1 (-7.11 kJ/mol)
(d)

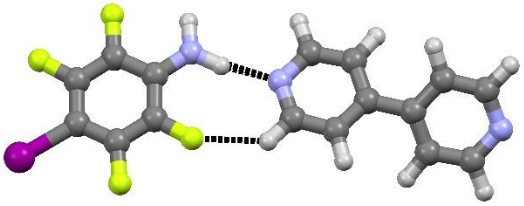

N1- H1A···N2/C10-H10···F4 (-40.12 kJ/mol)
(e)

Figure S72 Heteromolecular non-covalent bonded dimers used for the calculation of interaction energy (given in parentheses) from optimized geometry of (**itfa**)(**bpy**) cocrystal.

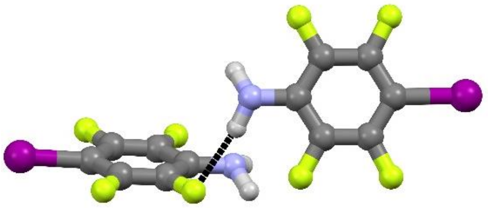

N1-H1···F4 (-14.49 kJ/mol)
(a)

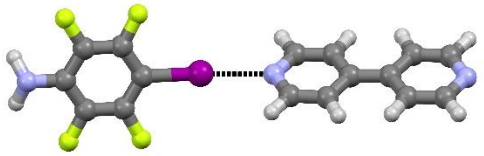

C4-I1···N2 (-33.39 kJ/mol)
(b)

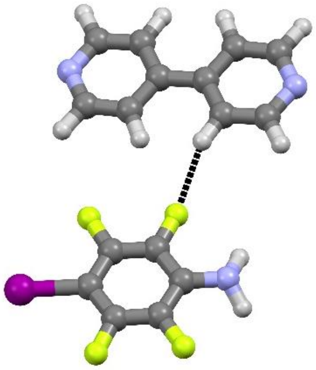

C10-H10···F1 (-11.77 kJ/mol)
(c)

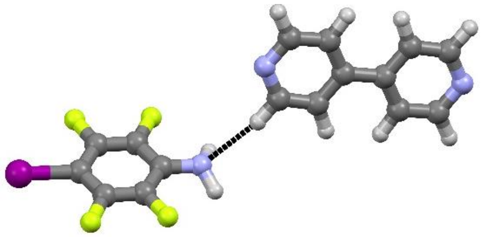

C7-H7···N1 (-2.54 kJ/mol)
(d)

Figure S73 Heteromolecular non-covalent bonded dimers used for the calculation of interaction energy (given in parentheses) from optimized geometry of (**itfa**)$_2$(**bpy**) cocrystal.

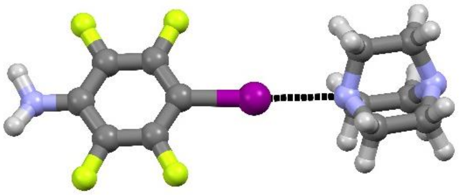

C4-I1···N2 (-44.13 kJ/mol)
(a)

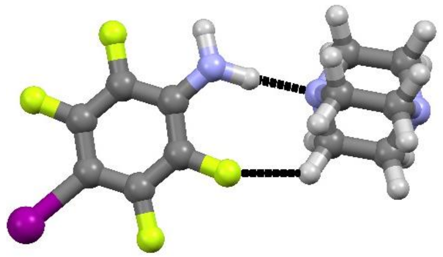

N1-H1B···N3/C12- H12A···F1 (-44.08 kJ/mol)
(b)

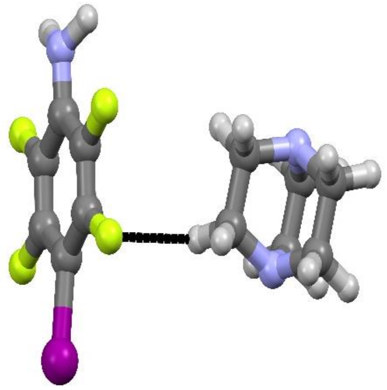

C10-H10B···F2 (-8.26 kJ/mol)
(c)

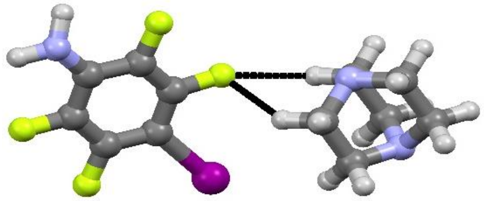

C11-H11B···F2/C10-H10A···F2 (-9.68 kJ/mol)
(d)

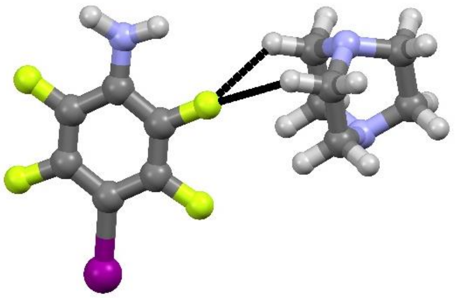

C12-H12A···F4/C10-H10B···F4 (-9.79 kJ/mol)
(e)

Figure S74 Heteromolecular non-covalent bonded dimers used for the calculation of interaction energy (given in parentheses) from optimized geometry of (**itfa**)(**dabco**) cocrystal.

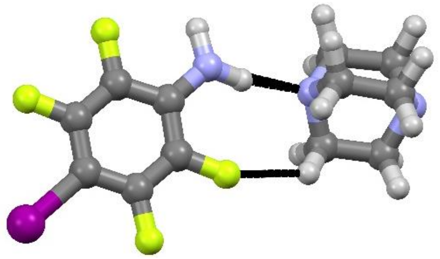

N2-H2A···N4/C18-H18B··· F5 (-42.74 kJ/mol)
(a)

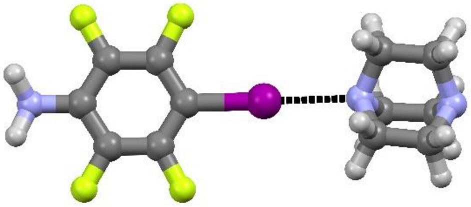

C4-I1···N3 (-45.91 kJ/mol)
(b)

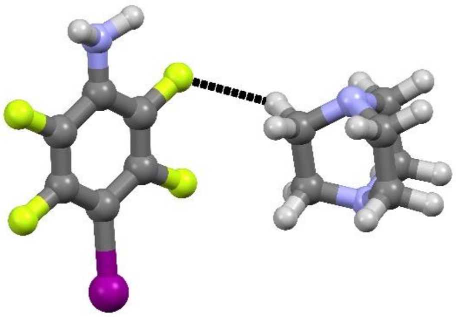

C18-H18A···F4 (-8.86 kJ/mol)
(c)

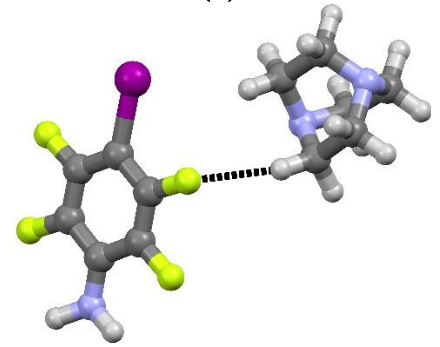

C15-H15A···F7 (-8.32 kJ/mol)
(d)

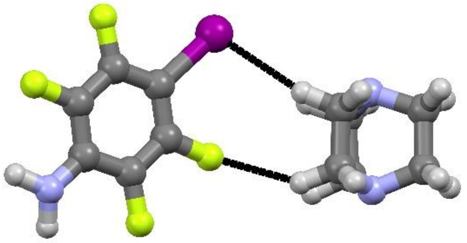

C13-H13A···I2/C18-H18B···F6 (-10.55 kJ/mol)
(e)

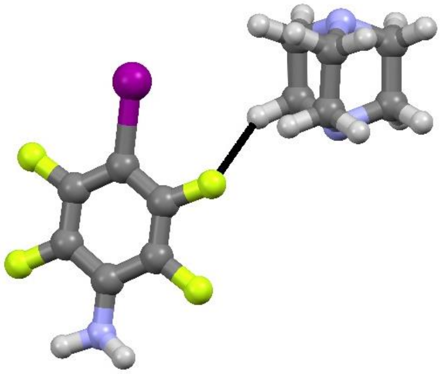

C16-H16A···F2 (-8.72 kJ/mol)
(f)

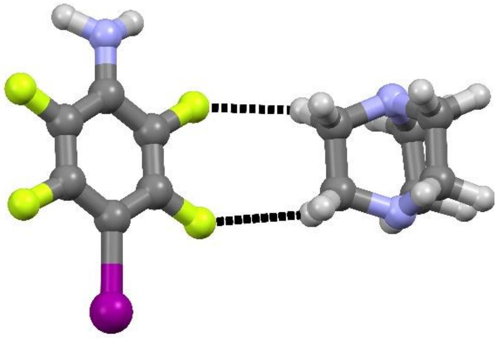

C15-H15A···F4/C18-H18A···F7 (-7.61 kJ/mol)
(g)

Figure S75 Heteromolecular non-covalent bonded dimers used for the calculation of interaction energy (given in parentheses) from optimized geometry of (**itfa**)$_2$(**dabco**) cocrystal.

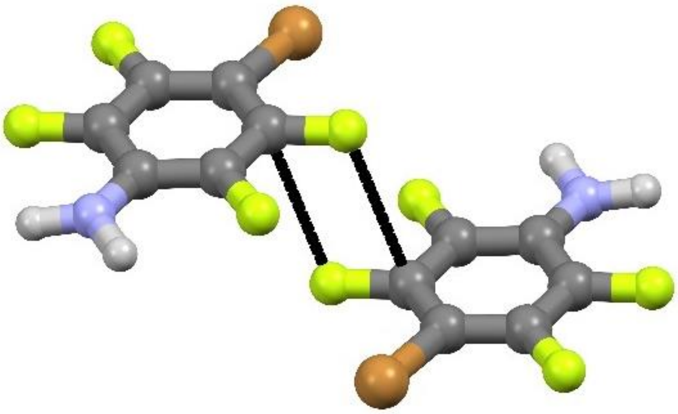

C5-F3···C5-F3 (-12.12 kJ/mol)

Figure S76 Homomolecular non-covalent bonded dimer used for the calculation of interaction energy (given in parentheses) from optimized geometry of (**btfa**)(**bpean**) cocrystal.

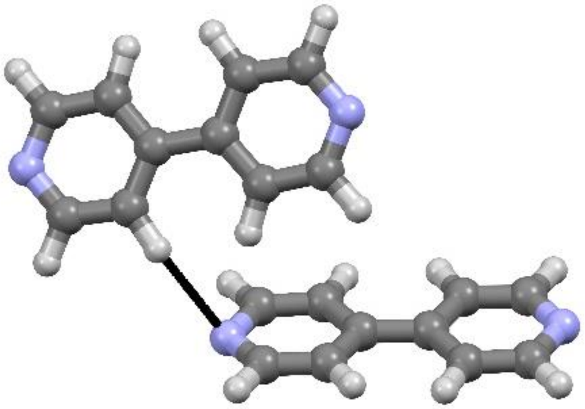

C8-H8···N3 (-20.48 kJ/mol)

Figure S77 Homomolecular non-covalent bonded dimer used for the calculation of interaction energy (given in parentheses) from optimized geometry of (**btfa**)(**bpy**) cocrystal.

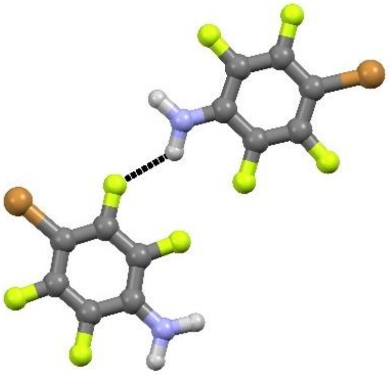

N2-H2A···F3A (-13.22 kJ/mol)
(a)

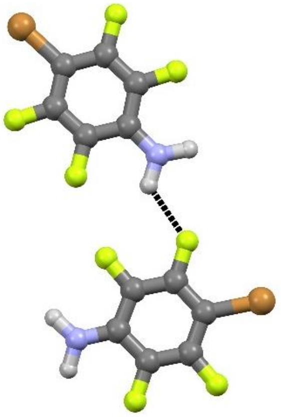

N1A-H1AA···F7 (-13.51 kJ/mol)
(b)

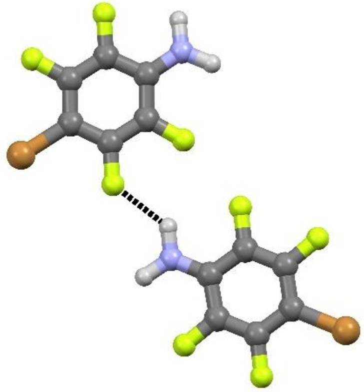

N2A-H2AB···F2 (-13.70 kJ/mol)
(c)

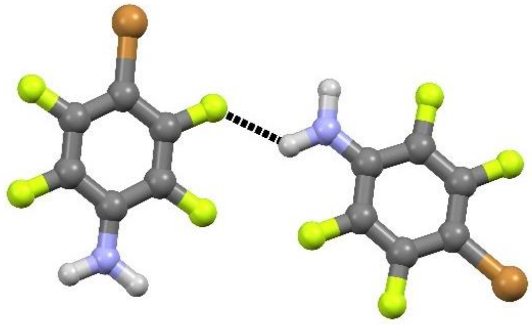

N1-H1B···F6A (-13.59 kJ/mol)
(d)

Figure S78 Homomolecular non-covalent bonded dimers used for the calculation of interaction energy (given in parentheses) from optimized geometry of (**btfa**)$_2$(**bpy**) cocrystal.

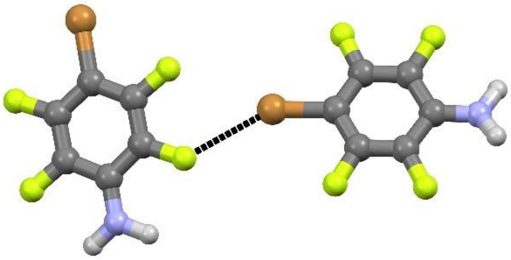

C88-Br8···F19 (-4.20 kJ/mol)
(a)

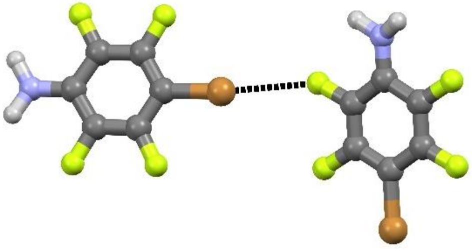

C82-Br7···F23 (-4.97 kJ/mol)
(b)

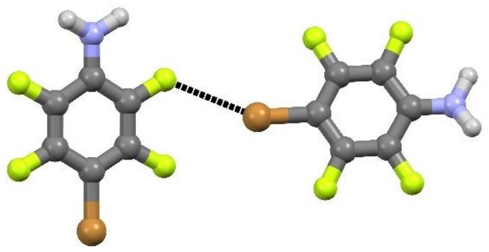

C70-Br6···F27 (-4.79 kJ/mol)
(c)

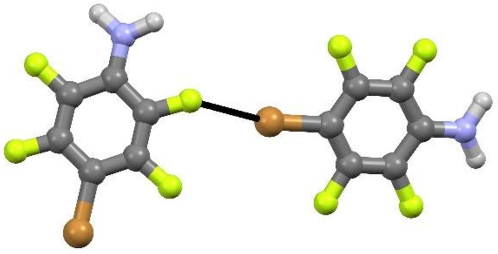

C64-Br5···F31 (-4.61 kJ/mol)
(d)

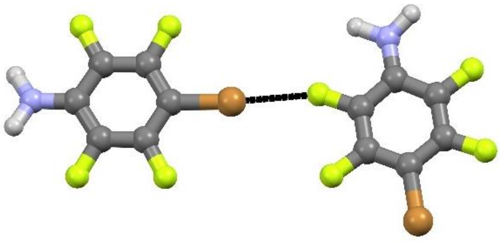

C46-Br4···F3 (-1.87 kJ/mol)
(e)

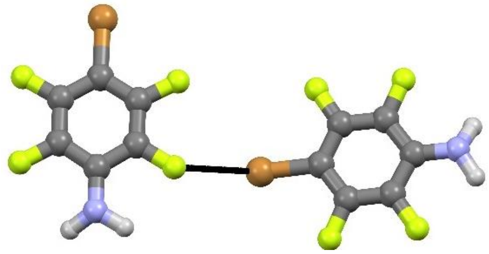

C34-Br3···F7 (-2.48 kJ/mol)
(f)

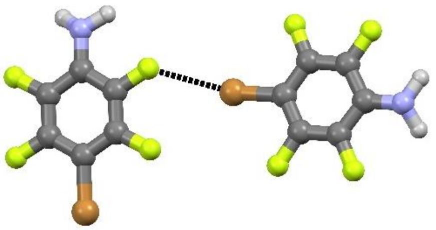

C16-Br2···F11 (-1.68 kJ/mol)
(g)

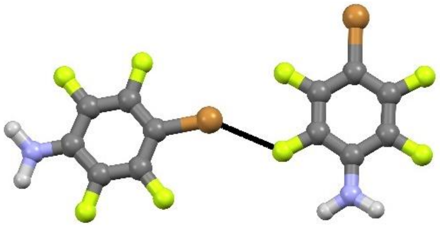

C4-Br1···F15 (-0.46 kJ/mol)
(h)

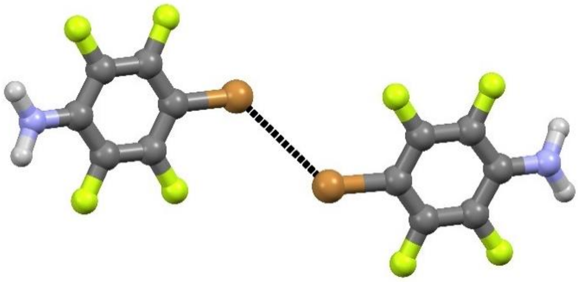

C82-Br7···Br3-C34 (-2.94 kJ/mol)
(i)

Figure S79 Homomolecular non-covalent bonded dimers used for the calculation of interaction energy (given in parentheses) from optimized geometry of (**btfa**)(**dabco**) cocrystal (*The labels are not consistent with the experimental*).

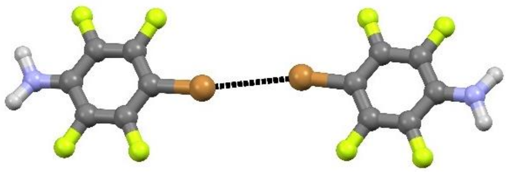

C10-Br2···Br1-C4 (-3.46 kJ/mol)
(a)

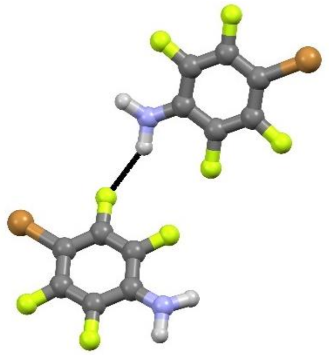

N2-H2B···F6 (-11.26 kJ/mol)
(b)

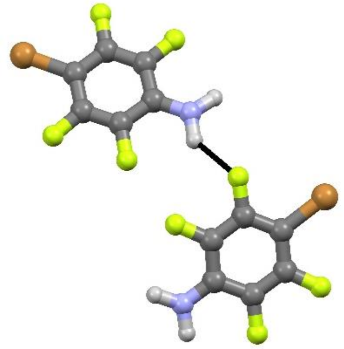

N1-H1A···F2 (-12.49 kJ/mol)
(c)

Figure S80 Homomolecular non-covalent bonded dimers used for the calculation of interaction energy (given in parentheses) from optimized geometry of (**btfa**)$_2$(**dabco**) cocrystal.

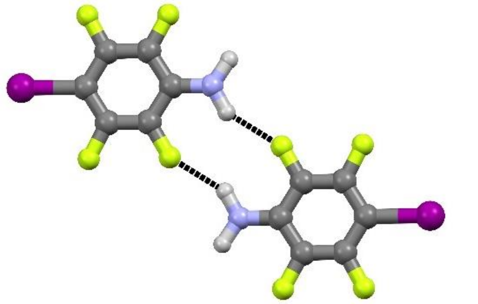

N1-H1A···F4 (-15.67 kJ/mol)
(a)

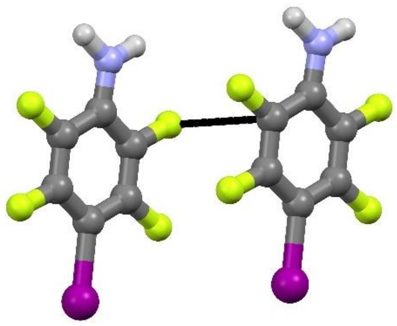

C6-F4···Pi (-12.43 kJ/mol)
(b)

Figure S81 Homomolecular non-covalent bonded dimers used for the calculation of interaction energy (given in parentheses) from optimized geometry of (**itfa**)(**bpean**) cocrystal.

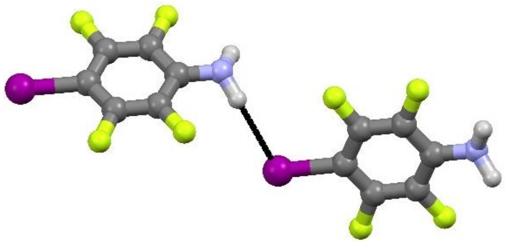

N1-H1A···I1 (-38.96 kJ/mol)
(a)

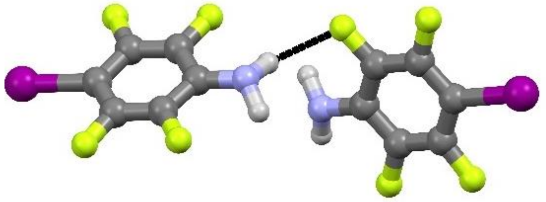

N1-H1B···F4 (-12.29 kJ/mol)
(b)

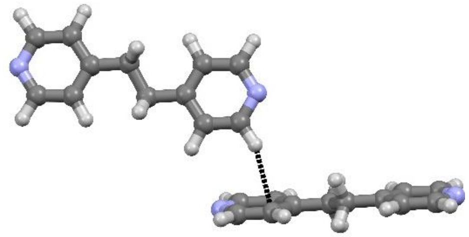

C10-H10···Pi (-16.72 kJ/mol)
(c)

Figure S82 Homomolecular non-covalent bonded dimers used for the calculation of interaction energy (given in parentheses) from optimized geometry of (**itfa**)$_2$(**bpean**) cocrystal.

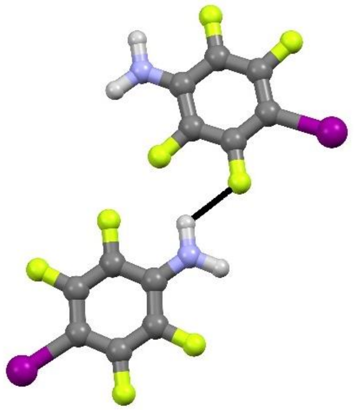

N1-H1B···F3 (-13.50 kJ/mol)
(a)

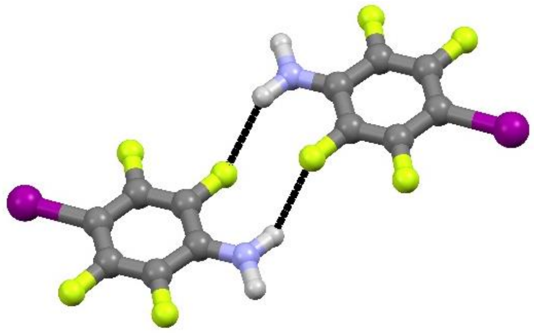

N1-H1B···F9 (-14.89 kJ/mol)
(b)

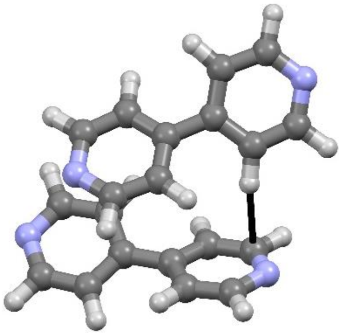

C13-H13···Pi (-24.70 kJ/mol)
(c)

Figure S83 Homomolecular non-covalent bonded dimers used for the calculation of interaction energy (given in parentheses) from optimized geometry of (**itfa**)(**bpy**) cocrystal.

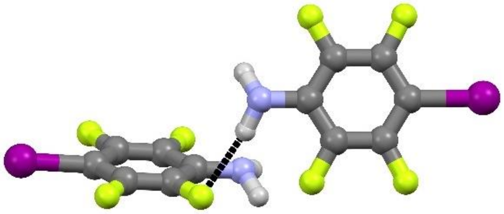

N1-H1···F4 (-14.49 kJ/mol)
(a)

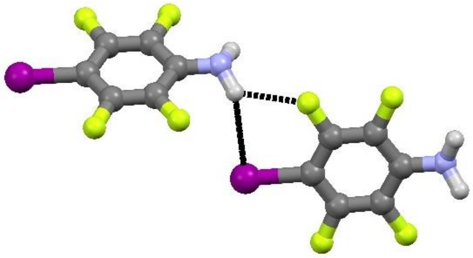

N1-H2···I1/ N1-H2···F2 (-15.06 kJ/mol)
(b)

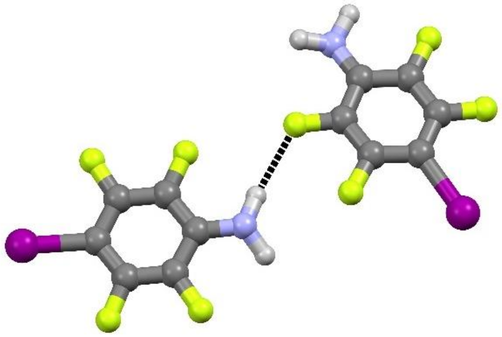

N1-H1A···F1 (-13.61 kJ/mol)
(c)

Figure S84 Homomolecular non-covalent bonded dimers used for the calculation of interaction energy (given in parentheses) from optimized geometry of (**itfa**)(**dabco**) cocrystal.

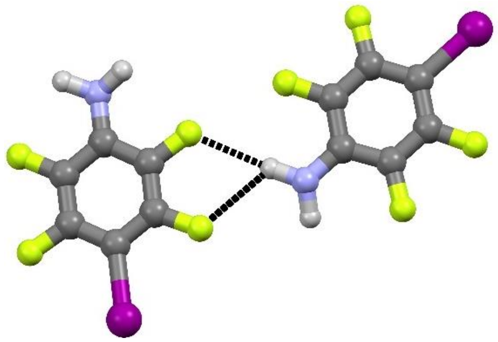

N2-H2B···F1/ N2-H2B···F2 (-14.10 kJ/mol)
(a)

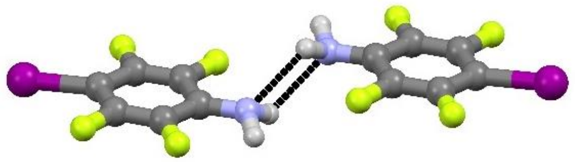

N1-H1A···N1 (-16.82 kJ/mol)
(b)

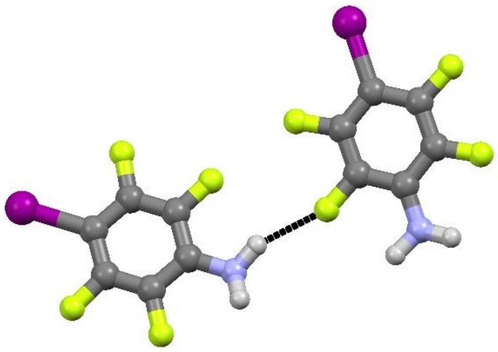

N1-H1B···F8 (-6.77 kJ/mol)
(c)

Figure S85 Homomolecular non-covalent bonded dimers used for the calculation of interaction energy (given in parentheses) from optimized geometry of (**itfa**)$_2$(**dabco**) cocrystal.
